# Supramolecular Click Chemistry for Surface Modification of Quantum Dots Mediated by Cucurbit[7]uril

**DOI:** 10.1021/acsnano.3c06601

**Published:** 2023-11-03

**Authors:** Katie McGuire, Suhang He, Jennifer Gracie, Charlotte Bryson, Dazhong Zheng, Alasdair W. Clark, Jesko Koehnke, David J. France, Werner M. Nau, Tung-Chun Lee, William J. Peveler

**Affiliations:** † School of Chemistry, Joseph Black Building, University of Glasgow, Glasgow, G12 8QQ, United Kingdom; ‡ School of Science, Constructor University, Campus Ring 1, 28759 Bremen, Germany; § James Watt School of Engineering, Advanced Research Centre, University of Glasgow, Glasgow, G11 6EW, United Kingdom; ∥ Institut für Lebensmittelchemie, Leibniz Universität Hannover, Callinstr 5, 30167 Hannover, Germany; ⊥ Institute for Materials Discovery, University College London, London, WC1H 0AJ, United Kingdom; # Department of Chemistry, University College London, London, WC1H 0AJ, United Kingdom

**Keywords:** quantum dots, cucurbiturils, host−guest complexes, click-chemistry, nanoparticles

## Abstract

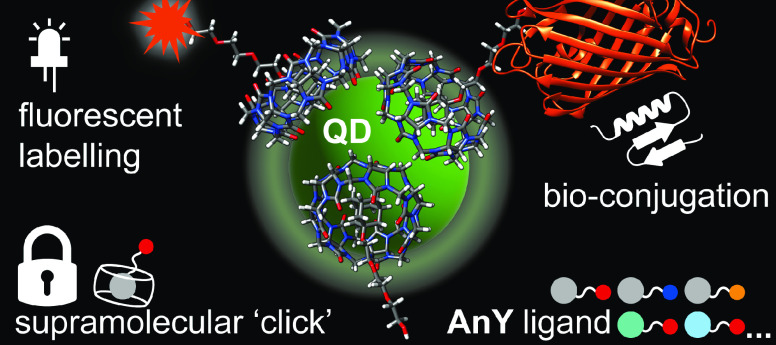

Cucurbiturils (CBs), barrel-shaped macrocyclic molecules, are capable of self-assembling at the surface of nanomaterials in their native state, via their carbonyl-ringed portals. However, the symmetrical two-portal structure typically leads to aggregated nanomaterials. We demonstrate that fluorescent quantum dot (QD) aggregates linked with CBs can be broken-up, retaining CBs adsorbed at their surface, via inclusion of guests in the CB cavity. Simultaneously, the QD surface is modified by a functional tail on the guest, thus the high affinity host–guest binding (log*K*_a_ > 9) enables a non-covalent, click-like modification of the nanoparticles in aqueous solution. We achieved excellent modification efficiency in several functional QD conjugates as protein labels. Inclusion of weaker-binding guests (log*K*_a_ = 4–6) enables subsequent displacement with stronger binders, realising modular switchable surface chemistries. Our general “hook-and-eye” approach to host–guest chemistry at nanomaterial interfaces will lead to divergent routes for nano-architectures with rich functionalities for theranostics and photonics in aqueous systems.

The functionalization of nanoparticles or nanostructured surfaces with chemical ligands is crucial for creating stable nanoscale materials or mesoscopically ordered architectures^1−4^ as well as interfacing the unique optoelectronic properties of nanoscale materials with the physicochemical environment for applications in switches^5−8^ or sensors.^9−12^ Key to these applications is the ability to efficiently modify nanoparticle surface chemistry at will, through strong chemical ligation, ideally, independently of the underlying core nanomaterial chemistry.^13^ “Click” chemical modification of nanoparticle surfaces, with one-step, reliable synthetic routes, is therefore a hugely attractive research goal.^14^ Furthermore, the ability to subsequently modify or regenerate the surface chemistry on nanoparticles leads to smart materials, sensors, material recovery, or recycling applications.^5^

“Click” chemical ligations at nanoparticle surfaces with the potential for further exchange will enable a huge variety of small molecules or biomolecules to be easily installed in a versatile scheme and dynamic covalent chemistry is one elegant solution. For example, Kay et al. as well as Milliron et al. produced a variety of molecular ligand systems featuring click-able and switchable moieties, through non-reversible azide-alkyne chemistry (CuAAC and SPAAC),^15^ as well as dynamic and reversible hydrazone^15−17^ and imine chemistries,^18^ on Au and metal oxide nanoparticle surfaces. This enables nanoparticles to be simply,orthogonally modified to act as labels or to form dynamic aggregates, but this approach requires specific pairs of functional groups. DNA-based ligation using toe-hold mediated strand displacement and base-pairing has also been used to great effect, with high precision and reversible surface modification.^19−21^ However, due to the complexity of modifying the DNA for attachment, ligation strategies typically rely on traditional click chemistry or other covalent surface modification,^22^ with varying efficiency. DNA is also highly charged, frequently creating undesirable aggregative effects.

An alternative approach is to apply supramolecular host–guest binding on the surface of nanoparticles. If the binding affinity between host and guest is sufficiently strong, and if the kinetics of dissociation are slow, then this can be regarded as highly efficient “non-covalent click-chemistry”. Such chemistry is orthogonal to its covalent click counterpart and adheres to Sharpless’ principles by being simple to perform, modular, wide in scope, high yielding and producing no by-products requiring separation. Furthermore, one supramolecular host can be applied to bind a wide variety of different guests of diverse binding affinities and chemistry without the need to covalently modify the nanoparticle surface, thereby greatly increasing the versatility of ligations possible.^23−25^ In particular, multimodal stimuli-responsivity and triggered release of functional moieties at the nanoparticle surface can be readily achieved via careful engineering of the binding affinities between each component in the system, which remains a daunting task when using covalent click chemistry due to the largely restricted choices of available bonds.

All precedent examples of non-covalent click chemistry at nanoparticle surfaces have involved the covalent attachment of modified β-cyclodextrin (β-CD) to gold nanoparticle (Au NP) or other nanoparticle surfaces. For example, Velders and co-workers used thiolated β-CD as an Au NP surface-bound host, capable of complexing ferrocene and adamantyl guests, to sense horseradish peroxidase via oxidation of the ferrocene guest and subsequent decomplexation from the nanoparticle surface.^26^ Likewise, Montenegro et al. recently applied a similar scheme using multivalent adamantyl anchors to-modify the surface of Au NPs inside cells with pegylated peptides.^27^ Ravoo and co-workers have exploited thiolated β-CD or carboxylate-modified β-CD to link together Au NPs, iron oxide NPs and LiYF_4_ upconverting NPs with a photoswitchable azobenzene guest, to create a modular system capable of reversible self-assembly.^6,28^ Others have also exploited this approach for patchy gold-on-polystyrene microparticles.^29^ In each case, controlling molecular cross-linking of the nanoparticles was the aim, with limited applications for labelling and sensing.

Whilst the use of β-CD was successful in providing an approach to non-covalent clickable nanoparticle surface modification, the binding affinities (*K*_a_) for the majority of guests are relatively low (10–10^5^ M^–1^) and β-CD exhibits fast exchange kinetics due to lack of constrictive binding,^30,31^ resulting in low functionalization efficiency and chemical stability of the resultant nanomaterials. Furthermore, β-CD must be selectively modified to enable nanoparticle surface attachment at one face. Thus, attention has recently turned to cucurbit[*n*]urils (CB*n*), water soluble macrocyclic oligomers comprising “*n*” (typically 5–8) glycoluril monomers, that offer binding affinities in the range of 10^4^–10^15^ M^–1^, i.e., up to 10 orders of magnitude larger than the strongest host–guest complexes of β-CD, and due to the constrictive binding by the carbonyl portals, slow dissociation kinetics (Figure 1).^32−34^ Thanks to the favorable interaction of the pre-organized 5–8 carbonyl groups around both rims of CB*n*, direct attachment of CB*n* to nanoparticle surfaces is possible.^35,36^ Unfortunately, due to the two symmetrical portals, attachment of CB*n* typically results in spontaneous aggregation of nanoparticles, bridged by CB*n*.^37,38^ Applications are therefore currently limited to entrapping molecular analytes in polydisperse nanoparticle aggregates, e.g., for luminescent or Raman detection,^36,39−42^ and some limited reversibility of aggregation has been shown.^43,44^ Early efforts on attaching CB*n* onto QD surface via their equatorial positions was made with limited success, owing to the challenge in functionalization of native CB*n*.^45^

**Figure 1:**
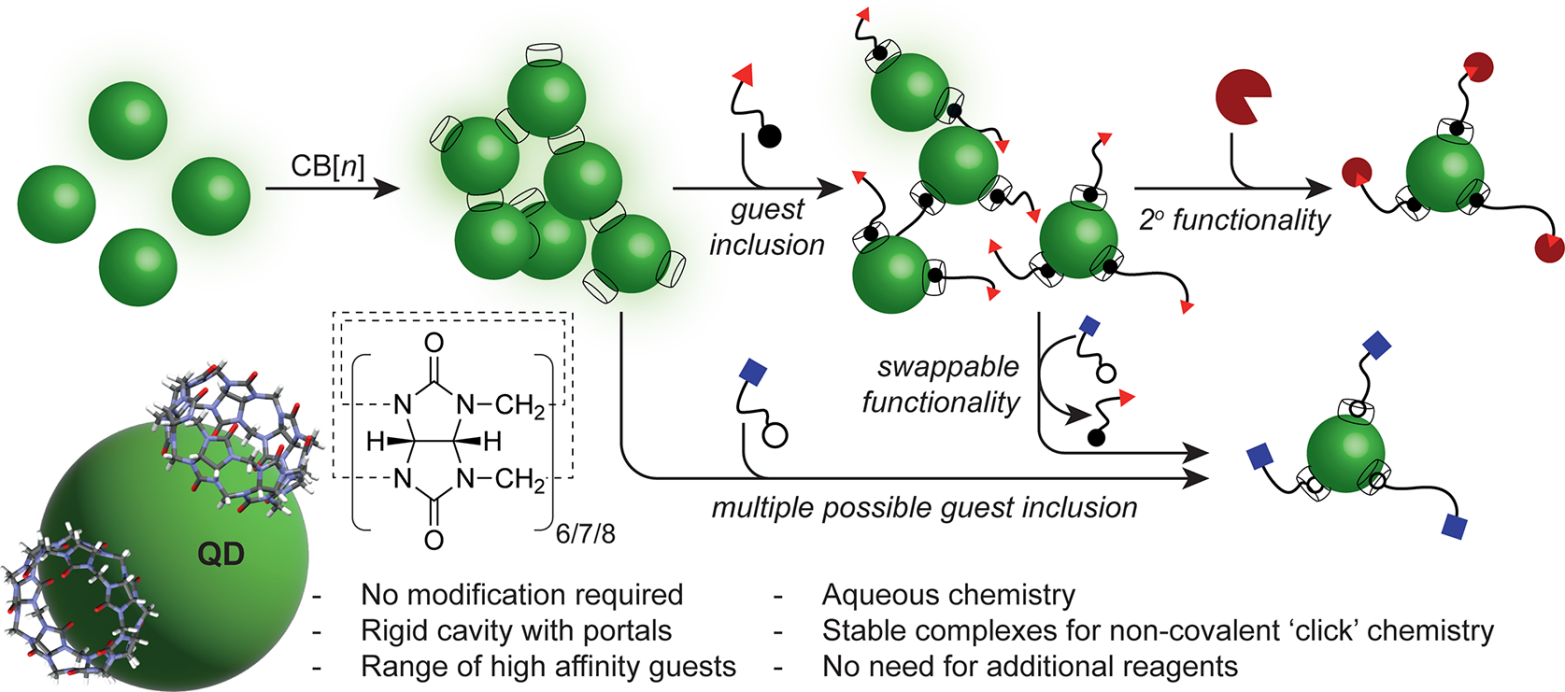
Simple synthetic strategy to modify nanoparticle surfaces using host–guest chemistry via cucurbit[*n*]uril in aqueous solution. The strong host–guest complexes (log*K*_a_ = 4–15) formed at the nanoparticle surfaces allow for robust modification with a wide variety of molecular guests, featuring a functional distal end, without the need for any additional catalysts or reagents, and with high functional group tolerance. These functional surfaces can feature reactivity for secondary modification, or can be “swapped” *in situ* by the appropriate choice of a competing guest.

Recently, we demonstrated the use of CB*n* to aggregate fluorescent colloidal quantum dots (QD) into small (<1 μm) aggregates (Figure 1) for sensing applications.^46^ The method was successfully applied by Scherman et al. to control Au-QD aggregate dynamics and to explore energy transfer.^47^ QDs are an important class of luminescent materials for sensing and labelling that rely on variable surface chemistry to fully exploit their utilization.^1,48^ During our initial work, we noticed that certain larger guests appeared to lead to disaggregation of the QD clusters, however, it was not clear whether this was due to inhibition of the two portal binding of CB*n* by the guest or the removal of CB*n* from the nanoparticle surface. Building on this serendipitous observation, we now demonstrate that CB*n* can indeed be utilized in a host–guest system for non-covalent “click” modification of QD surfaces (Figure 1). We use fluorescence assays as well as light scattering and other techniques to verify that CB7–guest complexes are retained at the surface, and that QD-CB7 nano-aggregates can act as stable intermediates which can subsequently be broken up by a variety of functional guests, allowing facile and efficient alteration of nanoparticle surface chemistry by swapping the guests or modifying the distal functional moieties. Finally, we demonstrate how such a surface modification approach could be applied in optical labels (including fluorescent proteins), bio-sensors, and triggered delivery systems to produce a general design strategy for non-covalent nanoparticle surface modification.

## Results and Discussion

**Figure 2:**
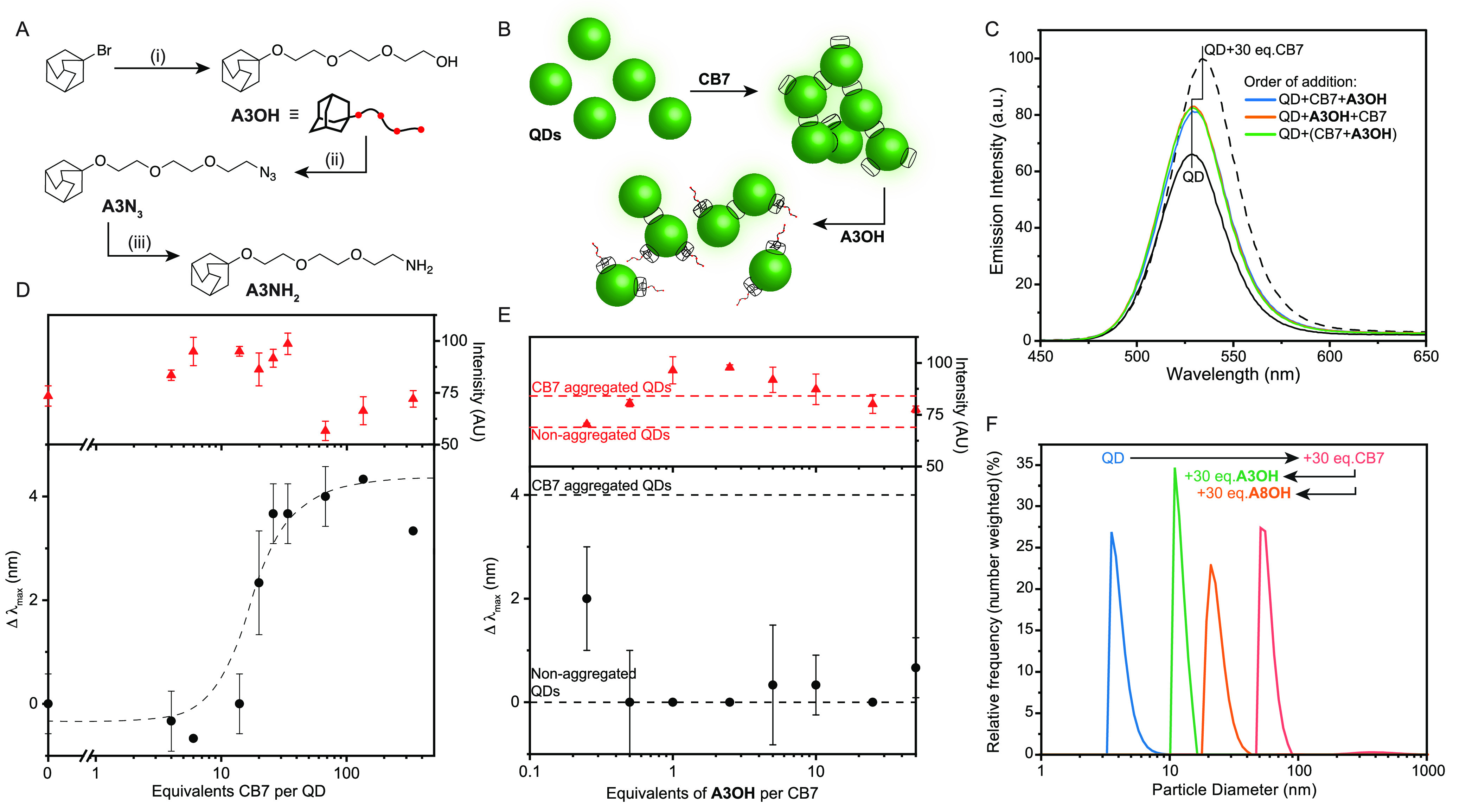
(A) Synthetic route to adamantyl derivatives: (i) triethylene glycol, NEt_3_, DBU, 110 °C, 18 h, 64% yield; (ii) mesyl chloride, NEt_3_, DCM, RT, 20 h, then NaN_3_, DMF, 80 °C, 20 h, 52% yield; (iii) PPh_3_, H_2_O, THF, RT, 24 h, 65% yield. (B) Schematic representation of the aggregation and disaggregation process. (C) Change in emission intensity and *λ*_max_ of CdTe530 upon aggregation with 30 equiv of CB7 and subsequent disaggregation with 30 equiv of **A3OH**; when the order of addition was varied, comparable results were obtained. (D) Titration of CdTe530 QDs (10 μM) with 0–400 equiv of CB7 showing the change in *λ*_max_ (black circles) and the change in emission intensity (red triangles). Data are averages of three independent replicates and error bars refer to the standard deviation. Trend line is to guide the eye. (E) Change in *λ*_max_ of CB7 aggregated CdTe530 (30 equiv) with increasing equivalents of **A3OH** per CB7 added (black circles), along with changes in measured emission intensity (red triangles). Error bars are averages with standard deviation of three independent replicates. (F) DLS measurements (number-weighted) for CdTe530 (10 μM), aggregated with 30 equiv of CB7, and subsequently disaggregated with 1 equiv of **A3OH** or **A8OH** per CB7 (30 equiv per QD). Measures are average of at least 2 technical replicates.

We first demonstrated that CB7 aggregates QDs as previously observed (Figure 2B). The QDs used in this work are predominantly CdTe530. They were synthesized in water, as described in the Supporting Information, and capped with 3-mercaptopropionic acid (MPA). CdTe530 QDs feature an emission maximum at ca. 530 nm and a full width at half maximum (FWHM) of ca. 40 nm (Figure 2C). Particle size was estimated by transmission electron microscopy (4.7 ± 1.2 nm N = 144, Figure S1) and by dynamic light scattering (DLS) (4.5 ± 1.0 nm, volume weighted, 3.8 ± 0.6 nm, number weighted Figure S2).

Multiple batches were produced and showed consistent behavior. Additional QDs used for testing included larger CdTe (CdTe540) and more monodisperse CdSe/ZnS514. The CdSe/ZnS QDs were synthesized via “hot injection” in organic solvents, before phase transfer to water via ligand exchange with reduced glutathione (GSH). The emission maximum was 514 nm, and FWHM was ca. 35 nm (Figure S3). Stock concentrations were estimated based on the method of Peng et al.^49^ and calculations are given in Table S1.

CB7 was prepared and isolated using standard literature methods, and 0–400 equiv of CB7 were added to aqueous QD stocks. As previously reported,^46^ addition of more than ca. 10 equiv of CB7 per QD led to a red shift of the CdTe luminescence by up to 4 nm for 30 equiv. This red shift was accompanied by a slight brightening of emission until the red shift reached a plateau, followed by a loss of intensity upon further CB7 addition (Figure 2D). The red shift arises from the formation of QD aggregates, where energy transfer occurs from slightly smaller QDs to slightly larger QDs within the population, leading to preferential emission from the larger QDs within the population.^50−52^ Beyond ca. 70 equiv of CB7, it was noted that large aggregates flocculated out of solution, accompanied by a decrease of luminescence intensity. The aggregation process rapidly completed with 30 equiv of CB7 per QD and maintained brightness and colloidal stability for days, so this ratio was chosen for further experiments, unless otherwise stated. We estimate that each QD is capable (based on a surface area approximation and a footprint of CB7 modelled as a circle)^53^ of binding 10–20 CB7 molecules, such that a slight excess of CB7 is potentially present in solution (Table S2). It is important to note that these QD aggregates are dynamic, allowing materials to diffuse into and out of the clusters formed.^46^

### Molecular Guest-Mediated Disaggregation of QD Aggregates

To explore the break-up of these stable nano-aggregates by guest addition to the surface-bound hosts (Figure 2B), we synthesized a series of adamantyl-linked polyethylene glycols (PEGs), with high affinity for CB7. Adamantane derivatives are reported to serve as strong binders for the hydrophobic cavity of CB7, and they do not occupy both portals.^33,54^ We hypothesise that the absence of two-portal binding is important to prevent host removal from the nanoparticle surface, and we illustrated this via molecular docking and density functional theory (DFT) simulations as well as NMR (Figure S4). The hydrophilic PEG chain acts as a spacer and solubilising group selectively on the nanoparticle surface, and it is trivial and convenient to alter the distal end to create heterobifunctional ligands (Figure 2A). For these molecules we use the nomenclature **A*n*Y**, where **A** represents an adamantyl (or any other “anchoring”) head group, ***n*** is the number of monomers in the PEG chain, and **Y** is any distal chemistry (natively −OH, or subsequently modified). Simple examples include adamantyl-triethylene glycol, termed **A3OH**, and the longer adamantyl-PEG400 (8–9 ethylene glycol units on average), termed **A8OH**. Adamantyl ether derivatives are reported to have binding affinities as high as *K*_a_ = 10^10^ M^–1^, and we measured a representative interaction between CB7 and **A3OH** of *K*_a_ = 10^9^ M^–1^ via fluorescence displacement assays (Figure S5).^55^ The detailed synthesis of the various guests used in this study is given in the “Methods” section and the Supporting Information.

Upon addition of **A3OH** to the preformed QD aggregates with CB7 (30 equiv), a blue shift of the fluorescence peak was observed (Figure 2C,D), resulting from a break up of the aggregates and a loss of FRET within the population (Figure 2B). The same original QD PL was observed independent of whether the **A3OH** guest was added before CB7, or pre-complexed with CB7 and then added to the QDs. A titration of **A3OH** into the aggregates (Figure 2E) showed that the disruption of the aggregates was largely complete with even sub-stoichiometric amounts of guest added, owing to the high binding affinity of the adamantyl residue and the steric hindrance and aqueous solubility of the ethylene glycol tail. The need for a neutral species was highlighted by similar experiments with adamantane amines, where the amine induced luminescent quenching and underwent potential non-specific adsorption to the QD surface.

To further probe this disaggregating effect and to demonstrate that the luminescence changes originated from the disruption of the aggregates rather than simple interaction between QD and guest, we undertook dynamic light scattering measurements. Number-weighted, along with intensity-weighted and volume-weighted, measurements are shown for CdTe530 (Figures 2F and S6), before and after the addition of 30 equiv of CB7, and after further addition of 30 equiv of **A3OH** per QD. There is a clear growth in the maximum recorded size upon addition of CB7 (from ca. 4 nm to 70–80 nm number-weighted, or several hundred nm volume-weighted), corresponding to aggregate formation, followed by a reduction in size upon addition of **A3OH** (11 nm), suggesting that the aggregates are indeed broken up by host–guest binding (Figure 1C). It is notable that the final population size of the QDs does not return to the starting point, suggesting that there is additional molecular material on the surface, increasing the hydrodynamic radius (the estimated size of the CB7+**A3OH** unit is around 2 nm, so an expected increase in diameter of over 4 nm is reasonable when accounting for additional hydration layers). It is also plausible that a small number of dimers or other small aggregates may lead to larger observed size in this ensemble measurement. An identical DLS experiment was undertaken with the ∼3-times longer guest, **A8OH** (8–9 ethylene glycol repeat units in PEG400), and the DLS results showed that the size was first similarly reduced (by ca. 21 nm) but then increased over the bare QDs alone at higher concentrations of added **A8OH**, again suggesting added material (guest) bound to the QD surface (Figures 2F and S6).

We performed control experiments by adding **A3OH** to CdTe530 in the absence of CB7 and triethylene glycol to CB7-aggregated CdTe530, and observed no unexpected changes in *λ*_max_ of the QD solutions (Figure S7). The aggregation and disaggregation process could also be monitored by gentle centrifugation to pellet-aggregated QDs (Figure S8) and by gel electrophoresis (Figure S9). To demonstrate the generality of the effect with other QDs, we showed that the yellow CdTe540 sample could also be aggregated with CB7 and disaggregated with **A3OH** (Figure S10). A sample of CdSe/ZnS514 could also be aggregated and disaggregated, but due to the narrower FWHM and greater homogeneity of this sample, the aggregation-induced red shift was less discernible. However, the process could be successfully followed by DLS (Figure S11).

**Figure 3:**
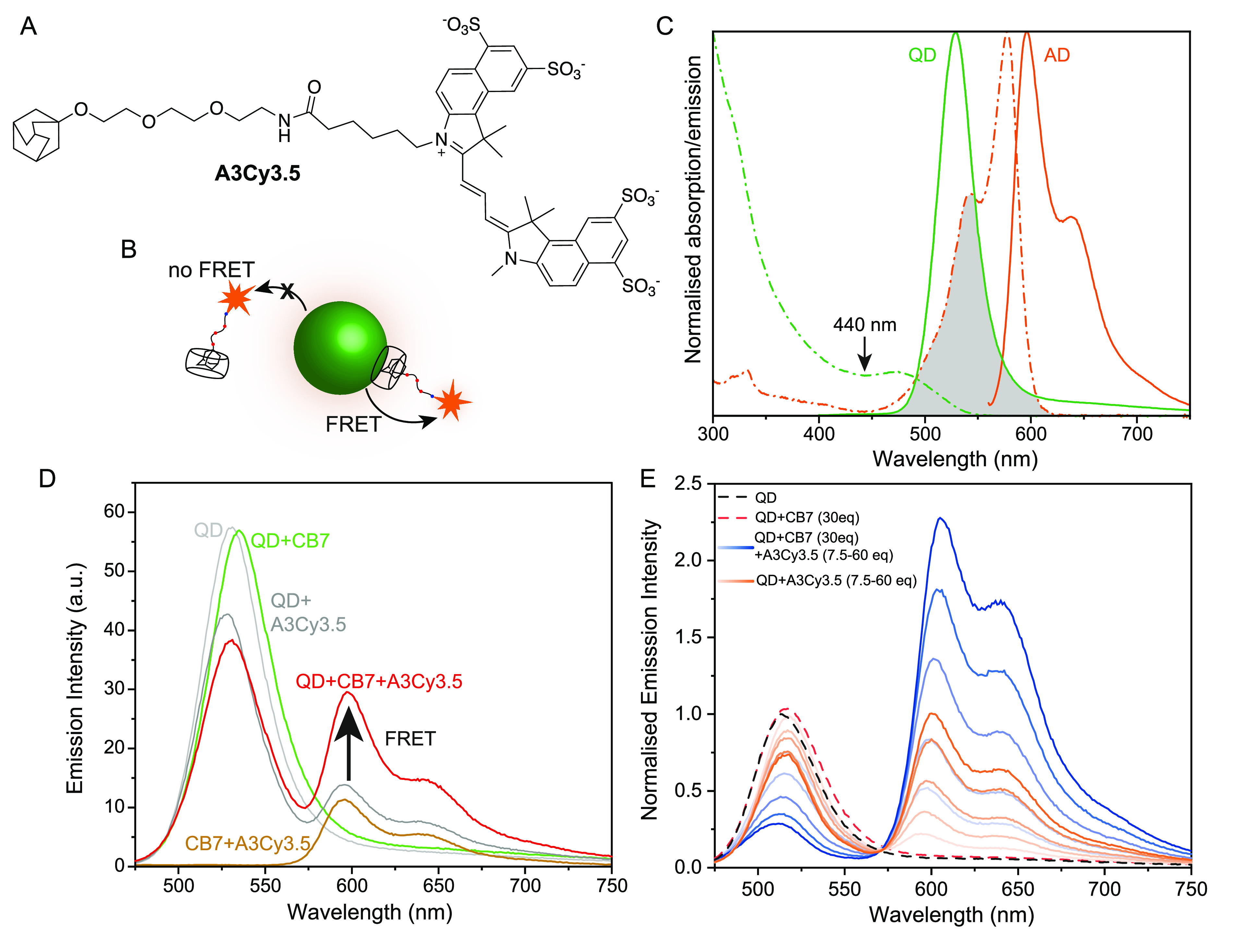
(A) Structure of dye-labelled **A3Cy3.5** and (B) a cartoon showing the possible association routes resulting in FRET if the host–guest complex remains at the QD surface. (C) Optical overlap of QD donor and Cy3.5 acceptor. Overlaid, normalized spectra, where dashed and dotted lines are absorbance and solid lines are emission. (D) Photoluminescence measurements of the system (QDs 3.3 μM, excitation: 440 nm) with each component (CB7 30 equiv, **A3Cy3.5** 30 equiv) added or excluded. The complete system is shown in red. Additional control experiments can be found in Figure S12. (E) Titrations of **A3Cy3.5** (7.5–60 equiv per QD) into aggregated QDs (30 equiv of CB7) shown in blue, or QDs alone shown in orange, showing FRET over the background emission caused by direct excitation. Data normalized to QD-only emission to more clearly show decrease in QD intensity.

While it is evident from the data that addition of **A3OH** successfully disrupts the QD-CB aggregation process, we needed to demonstrate that the host remains bound to the surface when intercalating the guest. To achieve this, we exploited a proximity-enabled Förster resonance energy transfer (FRET) process with a modified guest, **A3Cy3.5** (Figure 3A,B). The addition of a sulfo-cyanine fluorophore (Cy3.5) with complementary absorption to the QD emission (Figure 3C), to the adamantyl-PEG, meant that when QD and fluorophore were in very close proximity, i.e., bound via the adamantyl-CB complex, FRET should become observable from the QD to the dye. If the binding of the adamantyl ligands forces the entire host–guest complex to become detached from the surface of the QD, then FRET would not be observed. Sulfo-Cy3.5 was also chosen for its bulkiness and negative charge and low affinity with CB7, to avoid competition for the host between adamantyl and fluorophore, and to reduce non-specific absorption to the negative QD surface.

CdTe530 was aggregated with 30 equiv of CB7 and then **A3Cy3.5** was added. FRET was observed between the QD and fluorophore, over the background of Cy3.5 itself, a mixture of **A3Cy3.5** and CdTe530 without CB7 added, or a mixture of the QDs, CB7 and Cy3.5 without an adamantyl modification (Figures 3D and S12). As before, a blue shift of the QD-CB7 emission peak was also observed upon adding the adamantyl species. These combined observations suggest that CB7 does indeed remain on the QD surface when incorporating an adamantyl guest. Whilst it was impossible to find an excitation wavelength for the QD that did not cause some background signal from direct excitation of the dye, a titration with and without CB7 present showed the increased FRET over background signal (Figure 3E). If a large excess of **A3OH** was subsequently added, then a displacement of **A3Cy3.5** was observed (Figure S12) due to competitive displacement and reduction of FRET, but due to the similar binding strength of the two **A*n*Y** ligands and slow dissociation kinetics, a large excess was required (10–100 fold) to see an effect.

### Utilizing Molecular Guests at QD Surfaces for Particle Labelling via Specific Protein Binding

**Figure 4:**
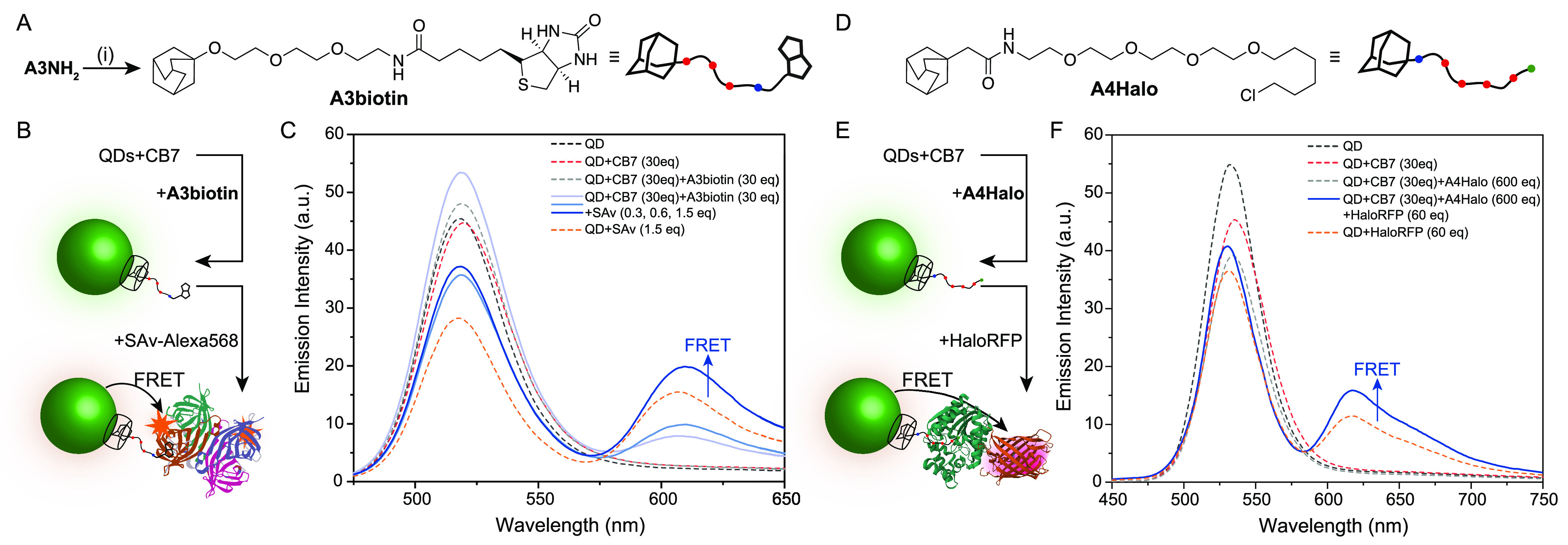
(A) Outline of the synthesis of **A3biotin** from A3NH_2_: (i) NHS ester of biotin, NEt_3_, DMF, 80 °C, 24 hrs, 72% yield. (B) Schematic representation of the QD labelling approach with dye-modified (Alexa568) streptavidin (adapted from RCSB (RCSB.org) Protein Data Bank (PDB) ID 3RY1 Stenkamp et al.^56^). (C) Titration of streptavidin-Alexa568 into CdTe530 (10 μM) aggregated with 30 equiv of CB7 per QD, and subsequently disaggregated with 30 equiv of **A3biotin** per QD (0.3–1.5 equiv of labelled streptavidin per QD shown in pale to dark blue). Control data of the direct excitation of streptavidin-Alexa568 are shown (orange dash). (D) Structure of the halogenated adamantyl ligand **A4Halo**, detailed synthesis is given in Supporting Information. (E) Schematic representation of the QD labelling approach with HaloTagged proteins, here red fluorescent protein (RFP) to induce FRET. Created from PDB IDs 5UXZ(57) and 2H5Q(58) with the Mol* web app.^59^ (F) Data for CdTe540 (10 μM) aggregated with 30 equiv of CB7 and disaggregated with 600 equiv of **A4Halo** per QD, after addition of halo-tagged RFP protein (blue line). The results for the mixture of QDs and protein alone is included to indicate direct excitation and non-specific binding background (orange dashed line).

Having demonstrated the surface affinity of the host–guest system, we sought to demonstrate the utility of the system for functional QD surface chemistry modification, using our non-covalent click-chemical approach. We synthesized the biotinylated adamantyl derivative **A3biotin** (Figure 4A) and demonstrated that it binds through the adamantyl residue to CB7 (Figures 4B and S13).

As expected, titration of **A3biotin** into the CdTe530/CB7 aggregates resulted in a blue shift of the QD aggregate emission peak (Figure 4C), suggesting the QDs are dispersed with biotin now present on the surface via the CB7/adamantyl linkage. Subsequent addition of a fluorescently labelled streptavidin (SAv-Alexa568) capable of a strong and specific biotin-avidin interaction, with good potential optical overlap with the QD donor (Figure S14), resulted in FRET being observed, demonstrating that a functional surface has been formed at the interface of the QD (Figure 4C).^60^ Additional DLS experiments using a nonfluorescent NeutrAvidin also showed the expected size increase on addition to the A3biotin held at the QD surface by CB7 (Figure S15). In both cases, lack of the biotinylated guest at the surface stops the specific ligand-protein interaction. Direct attachment of adamantyl-decorated proteins should also be possible in the future, as has been demonstrated with thiolated β-CD at planar Au surfaces.^61^

As a further demonstration of post-functional modification of the non-covalent click system, we synthesized a series of adamantyl derivatives featuring a PEG linker and alkyl chloride tail, suitable for reacting with HaloTagged proteins (**A3Halo**, **A4Halo,** and others). HaloTag is a recombinant modified protein fragment derived from a bacterial enzyme, that can be expressed as a linked tag to other recombinant proteins, and rapidly and specifically forms a covalent bond with small molecule alkyl halides.^62^ Among the synthetic alkyl chlorides produced, the acetamide-linked **A4Halo** (Figure 4D) was the most practicable and displayed a lower *K*_a_ of 10^4^ M^–1^ (via slow exchange NMR titration, Figure S16). A small amount of DMSO (5%) was required in these experiments, and whilst lowering the host–guest affinity below the common values of neutral adamantyl derivates in CB7 (typically ∼10^9^ M^–1^, discussion Figure S16), it did not unduly impact the aggregation or disaggregation process.^63^ The stronger binding, ether-linked **A3Halo** was also synthesized but was not sufficiently water soluble for use here. **A4Halo** was also capable of preferentially binding to the CB7 pocket and disrupting the QD/CB7 aggregates (Figure 4E), as demonstrated by a blue shift of the aggregated emission (Figures 4F and S17). Red fluorescent protein modified with a HaloTag (HaloRFP) was expressed in *E. coli* as a good FRET acceptor for the CdTe540 QDs (Figure S18). The purified protein was added to the **A4Halo**-modified QDs and FRET indicative of proximal binding was observed when the taggable alkyl halide was held at the QD surface by CB7 (Figure 4F). There was a degree of non-specific binding of the HaloRFP protein to the CdTe540 QDs seen in this measurement (when no alkyl halide was present), leading to higher background energy transfer, but the energy transfer was greater in the complete non-covalent click system. This system could be further optimized in the future by changing the background ligand surface around the CB7 of compact antifouling ligands, e.g., zwitterionic sulfobetaine-based ligands.^64^

### Swapping and Triggered Release of Molecular Guests from QD Surfaces by Noncovalent Click Chemistry

**Figure 5:**
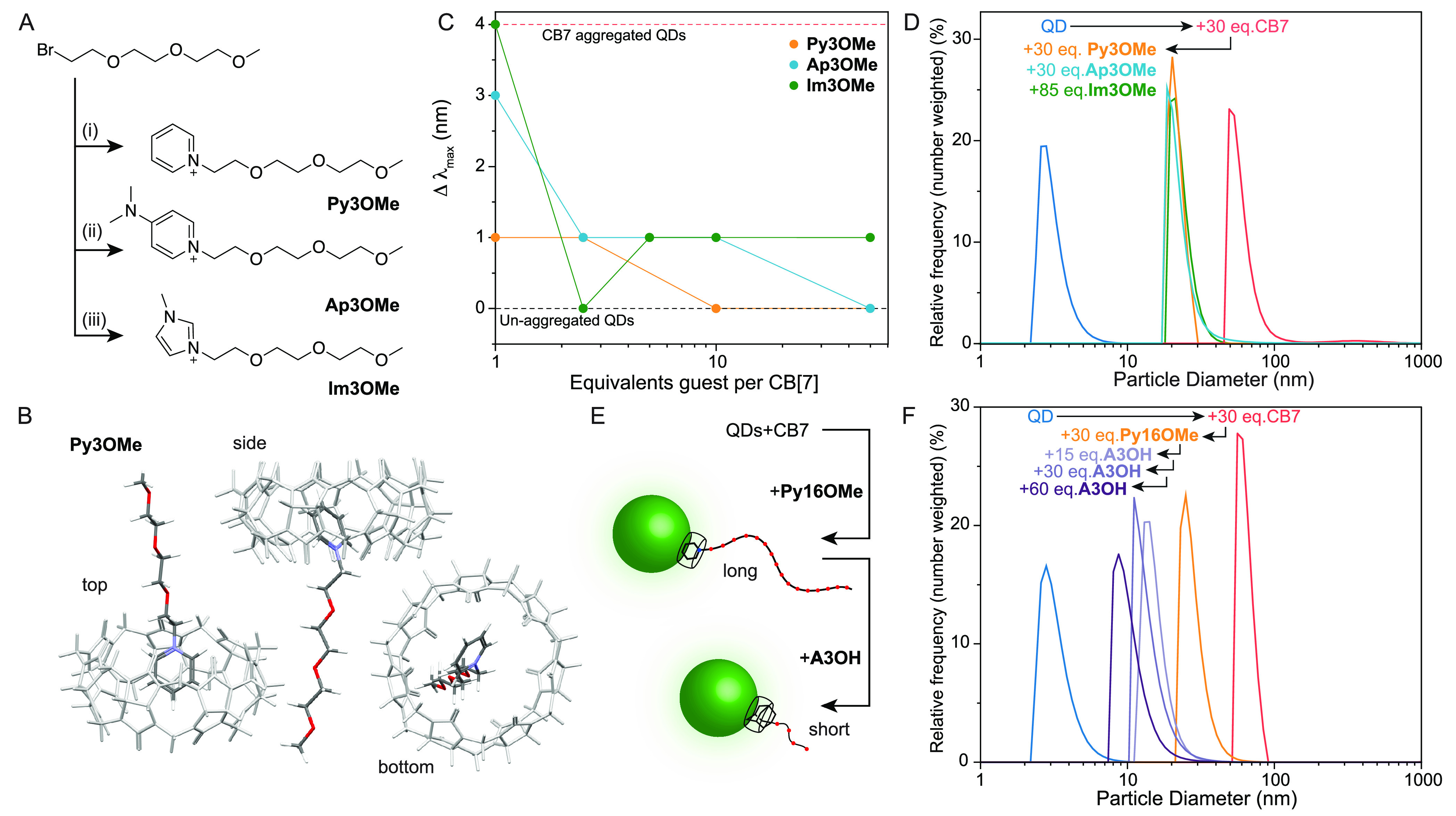
(A) Outline of the synthetic route to pyridinium and imidazolium derivatives from bromo-triethylene glycol monomethyl ether and (i) pyridine, (ii) 4-dimethylamino pyridine, or (iii) 1-methyl imidazole, DMF, 100 °C, 20 h, yields between 25 and 72%. (B) DFT model of Py3OMe complexed to CB7 from three angles. (C) Change in *λ*_max_ for CdTe530 (10 μM) aggregated by 30 equiv of CB7 and subsequently disaggregated by one of the three guests with increasing concentration. (D) DLS of CdTe530 (10 μM) aggregated with 30 equiv of CB7 and disaggregated with one or more equivalents of each of the three guests per CB7, resulting in a reduction in measured size. (E) Schematic representation of the sequential disaggregation and guest-swapping on the QD surface with a long, weakly bound guest (**Py16OMe**) and a short, strongly bound guest (**A3OH**). (F) DLS measurements of CdTe530 (10 μM), aggregated with 30 equiv of CB7 per QD and disaggregated with 1 equiv of long **Py16OMe** per CB7, before titrating in increasing equivalents of short **A3OH** (0.5–2 equiv per CB7), with a concomitant reduction in measured particle size.

To demonstrate the diversity of host–guest chemistry that might be leveraged in the non-covalent click system, we designed and implemented other ligand sets capable of disaggregating the CB7 aggregated QDs. Three different ligands were successfully synthesized (Figure 5A) with positively charged ammonium headgroups for binding to CB7 and monomethyl ether triethyleneglycol tails: **Py3OMe**, **Ap3OMe**, and **Im3OMe**. All were designed to display only medium affinity but also to act as single portal binders for CB7, to allow their subsequent displacement by stronger binders, e.g., those based on adamantyl. The “goodness of fit” was estimated by DFT (Figures 5B and S19), and **Py3OMe** was predicted to be the most promising single portal binder to CB7. Experimentally, all three compounds were shown to bind CB7 via solution NMR measurements and were displaceable by **A3OH** (Figure S20). The absolute *K*_a_ values for CB7 were determined by isothermal calorimetry (ITC), with values of 10^4^ M^–1^ for **Py3OMe**, 10^5^ M^–1^ for **Im3OMe** and 10^6^ M^–1^ for **Ap3OMe** (Figure S21). A range of longer-tailed versions using monomethyl ether PEG750 (16–17 ethylene glycol units on average) were also produced, including **Py16OMe**, **Im16OMe**, and **Ap16OMe**.

All three guest molecules could disaggregate the QD/CB clusters as shown both by blue shifting the measured emission maximum, indicating break up and loss of FRET between the QD sub-populations, and by reduction of size, measured by DLS (Figures 5C,D and S22). It is noteworthy that the pyridinium derivative **Py3OMe** appeared to disaggregate the clusters most reliably, and in the case of the methyl imidazolium derivatives, **Im3OMe** or **Im16OMe**, additional equivalents were required to effect the disaggregation process. Given the larger and more elongated sizes of the head groups in **Im*n*Y** and **Ap*n*Y** guest head groups (Figure S19), the less efficient disaggregation, despite high affinities, and the slightly greater than expected drop in nanoparticle size (by DLS, Figure S22), we presume that these guests are removing some of the host. The aromatic residues protrude more deeply into the CB7 cavity and interact with the second CB7 portal, reducing their surface affinity.

The lower affinities of these designed ligands should enable more trivial swapping by higher affinity alternatives such as adamantyl derivatives (Figure 5E). As a first proof-of-principle, CdTe530 QDs were aggregated with CB7, then disaggregated with the long-tailed **Py16OMe** resulting in a reduction in measured size by DLS from 60–70 nm to ca. 30 nm, indicating the presence of the long-chain species at the QD surface. Subsequent titration of this species with the shorter but much more strongly binding **A3OH** led to an extra observed decrease in size to ca. 10 nm, matching earlier observations for this system, suggesting the displacement of the pyridinium guest was successful (Figure 5F). Despite the high affinity difference, intermediate sizes (between 10–20 nm) do appear at less than 1:1 equivalence and may be due to slow exchange on the experimental time scale and mixed surfaces (long and short tail guests) or averaging effects in the DLS measurements.

## Conclusions

It is well known that CB*n* can aggregate different nanoparticles via two-portal electrostatic interactions between surface cationic sites and the carbonyls of each portal. In particular, while this type of aggregation phenomenon has been extensively demonstrated for Au and Ag NPs, we have reported the CB-mediated aggregation of metal chalcogenide NPs, particularly fluorescent CdTe QDs. Whilst we have previously discussed the choice of surface and stabilizing ligands on CB*n* affinity for the surface,^46^ we have now achieved disaggregation of these clusters via a rational molecular design of guests for the CB7 host that serves as a platform for robust non-covalent click chemical ligations. We confirmed via a range of analytical techniques, that the host–guest complex remains attached to the QD surface after ligation. We show how such a divergent approach with QD-CB nanoaggregates serving as the key intermediate, could lead to the post-synthetic modification of nanoparticle surfaces with a wealth of ligands, via demonstrations of multi-step protein ligations and “swappable” surfaces. Not only can a wide range of chemical functionalities be attached to the QD surface but also their binding strength and stimuli-responsivity can also be tailored to fit a specific application via careful selection from hundreds of guests in the literature with binding affinities spanning across ca. 10 orders of magnitude (10^4^–10^15^ M^–1^), which cannot be achieved using covalent click reaction schemes.

Our “hook-and-eye” approach^65^ is easily amenable in the future to create responsive nanoparticle gels^13^ or to tune surface-nanoparticle-target interactions in solutions or microarrays.^66^ It can be utilized for a wealth of labelling, drug delivery,^67,68^ sensing, and soft nanophotonic applications,^48^ in a highly robust yet flexible manner.

## Methods

Materials were reagent-grade or above, water was deionized to >15 MΩ. Alexa568-labelled streptavidin and unmodified Neutravidin were purchased from Thermo Fisher group, sulfo-Cy3.5 active ester was purchased from Lumiprobe, and biotin active ester was purchased from Tokyo Chemical Industry. All were used as received. Cucurbit[7]uril,^69,70^ molecular guests, and precursors were synthesized in house. Halo-tagged RFP was produced recombinantly in house. Detailed methods and further synthetic routes are provided in the Supporting Information. All experiments were carried out at ambient temperature (RT = 18–22 °C) unless otherwise noted.

Luminescence and absorption measurements were made on a Tecan Spark plate reader with monochromated light from a Xe flash lamp. Samples were measured in Corning half-area, UV-transparent, flat-bottomed 96-well plates (product no. 3679). Working volumes were 75 μL, unless stated otherwise, and for this volume the path length was estimated as 0.86 cm. Reagents (QDs, CB7, **A*n*Y**) were added sequentially to wells to aggregate and disaggregate the QDs with 5–10 minutes incubation and measurement times between the additions. A typical experiment used CdTe QDs at a final well concentration of 10 μM, aggregated by CB7 at a concentration of 300 μM (30 equiv). **A*n*Y** guest was added between sub-stoichiometric and molar excess as required.

### Synthesis of CdTe530

CdTe QDs were prepared following an adapted protocol from Tran et al.^71^ Cd(OAc)_2_ (46 mg, 0.2 mmol) and 3-mercaptopropionic acid (30 μL, 0.34 mmol) were dissolved in 40 mL of N_2_-purged water. This solution was then basified with 1 M aqueous NaOH until the solution was at pH 12, turning the cloudy solution clear. A portion of freshly prepared purple NaHTe solution (0.5 mL in a nitrogen-filled syringe) was rapidly added to the Cd solution under an N_2_ atmosphere, and a color change from colorless to orange was immediately observed. This orange solution was heated to 100 °C for 1 h and checked periodically with a hand-held 365 nm lamp and UV-visible/emission spectroscopy of needle tip aliquots, to ensure that the correct color was obtained. The product was estimated to be 30 μM in concentration by absorbance spectroscopy and stored at 4 °C in the dark before use. Prolonged storage or light exposure led to oxidation of the CdTe QDs, identified by a marked blueshift in their luminescence, which was avoided.

### Synthesis of A3OH as a prototypical AnY

The synthesis protocol was adapted from Gustafson et al.^72^ Triethylene glycol (9 g, 66.6 mmol), 1-bromoadamantane (2 g, 9.2 mmol), Et_3_N (4.2 mL, 30.0 mmol), and DBU (66 μL, 0.46 mmol) were magnetically stirred in a round-bottomed flask with condenser and heated to 110 °C for 18 h. The reaction was diluted with 25 mL 1 M aqueous HCl and extracted into DCM (2 × 25 mL). The organic layer was washed with water (2 × 25 mL) and dried with MgSO_4_ to yield a crude brown oil. The product was purified by column chromatography with a gradient of 0–20% MeOH in EtOAc. Fractions containing the product were identified by TLC and combined before the solvent was removed *in vacuo* to yield a deep yellow oil (1.68 g, 64.1% yield). ^1^H NMR (400 MHz, CDCl_3_) δ 3.74–3.52 (m, 12H, H_TEG_), 2.75 (s, 1H, H_OH_), 2.19–2.06 (m, 3H, H_Adm-bridgehead_), 1.78–1.67 (m, 6H, H_Adm_), 1.67–1.47 (m, 6H, H_Adm_). ^13^C NMR (101 MHz, CDCl_3_) δ 72.54, 72.35, 71.27, 70.69, 70.39, 61.79, 59.25, 41.44, 36.45, 30.51. MS (ESI+) *m/z* 307.1926 ([M + Na]^+^ C_16_H_28_O_4_Na^+^ expected 307.1880).

## Supporting Information Available

Detailed materials, methods and characterization data for all nanomaterials and molecules produced; DFT parameters; binding titrations; supplementary microscopy, spectroscopy, and images (PDF)

## References

[ref1] Algar W. R.; Massey M.; Rees K.; Higgins R.; Krause K. D.; Darwish G. H.; Peveler W. J.; Xiao Z.; Tsai H.-Y.; Gupta R.; et al. Photoluminescent Nanoparticles for Chemical and Biological Analysis and Imaging. Chem Rev 2021, 121, 9243–9358. 10.1021/acs.chemrev.0c01176. 34282906

[ref2] Murray C. B.; Kagan C. R.; Bawendi M. G. Self-Organization of CdSe Nanocrystallites into Three-Dimensional Quantum Dot Superlattices. Science 1995, 270, 1335–1338. 10.1126/science.270.5240.1335.

[ref3] Wang W.; Mattoussi H. Engineering the Bio–Nano Interface Using a Multifunctional Coordinating Polymer Coating. Acc. Chem. Res. 2020, 53, 1124–1138. 10.1021/acs.accounts.9b00641. 32427464

[ref4] Heuer-Jungemann A.; Feliu N.; Bakaimi I.; Hamaly M.; Alkilany A.; Chakraborty I.; Masood A.; Casula M. F.; Kostopoulou A.; Oh E.; et al. The Role of Ligands in the Chemical Synthesis and Applications of Inorganic Nanoparticles. Chem Rev 2019, 119 (8), 4819–4880. 10.1021/acs.chemrev.8b00733. 30920815

[ref5] Klajn R.; Stoddart J. F.; Grzybowski B. A. Nanoparticles Functionalised with Reversible Molecular and Supramolecular Switches. Chem. Soc. Rev. 2010, 39, 2203–2237. 10.1039/b920377j. 20407689

[ref6] Engel S.; Möller N.; Stricker L.; Peterlechner M.; Ravoo B. J. A Modular System for the Design of Stimuli-Responsive Multifunctional Nanoparticle Aggregates by Use of Host–Guest Chemistry. Small 2018, 14, 1704287 10.1002/smll.201704287. 29573341

[ref7] Cherkasov V. R.; Mochalova E. N.; Babenyshev A. V.; Vasilyeva A. V.; Nikitin P. I.; Nikitin M. P. Nanoparticle Beacons: Supersensitive Smart Materials with On/Off-Switchable Affinity to Biomedical Targets. ACS Nano 2020, 14, 1792–1803. 10.1021/acsnano.9b07569. 31944662

[ref8] Aldewachi H.; Chalati T.; Woodroofe M. N.; Bricklebank N.; Sharrack B.; Gardiner P. Gold Nanoparticle-Based Colorimetric Biosensors. Nanoscale 2018, 10, 18–33. 10.1039/C7NR06367A. 29211091

[ref9] Zahran M.; Khalifa Z.; Zahran M. A.-H.; Abdel Azzem M. Recent Advances in Silver Nanoparticle-Based Electrochemical Sensors for Determining Organic Pollutants in Water: A Review. Mater. Adv. 2021, 2, 7350–7365. 10.1039/D1MA00769F.

[ref10] Shan B.; Broza Y. Y.; Li W.; Wang Y.; Wu S.; Liu Z.; Wang J.; Gui S.; Wang L.; Zhang Z.; et al. Multiplexed Nanomaterial-Based Sensor Array for Detection of COVID-19 in Exhaled Breath. ACS Nano 2020, 14, 12125–12132. 10.1021/acsnano.0c05657. 32808759

[ref11] You C. C.; Miranda O. R.; Gider B.; Ghosh P. S.; Kim I. B.; Erdogan B.; Krovi S. A.; Bunz U. H.; Rotello V. M. Detection and Identification of Proteins Using Nanoparticle-Fluorescent Polymer “chemical Nose” Sensors. Nat. Nanotechnol. 2007, 2, 318–323. 10.1038/nnano.2007.99. 18654291

[ref12] Saha K.; Agasti S. S.; Kim C.; Li X.; Rotello V. M. Gold Nanoparticles in Chemical and Biological Sensing. Chem. Rev. 2012, 112 (5), 2739–2779. 10.1021/cr2001178. 22295941 PMC4102386

[ref13] Green A. M.; Ofosu C. K.; Kang J.; Anslyn E. V.; Truskett T. M.; Milliron D. J. Assembling Inorganic Nanocrystal Gels. Nano. Lett. 2022, 22, 1457–1466. 10.1021/acs.nanolett.1c04707. 35124960

[ref14] Idiago-López J.; Moreno-Antolín E.; de la Fuente J. M.; Fratila R. M. Nanoparticles and Bioorthogonal Chemistry Joining Forces for Improved Biomedical Applications. Nanoscale Adv. 2021, 3, 1261–1292. 10.1039/D0NA00873G. 36132873 PMC9419263

[ref15] Dominguez M. N.; Howard M. P.; Maier J. M.; Valenzuela S. A.; Sherman Z. M.; Reuther J. F.; Reimnitz L. C.; Kang J.; Cho S. H.; Gibbs S. L.; et al. Assembly of Linked Nanocrystal Colloids by Reversible Covalent Bonds. Chem. Mater. 2020, 32, 10235–10245. 10.1021/acs.chemmater.0c04151.

[ref16] della Sala F.; Kay E. R. Reversible Control of Nanoparticle Functionalization and Physicochemical Properties by Dynamic Covalent Exchange. Angew. Chem. Int. Ed. 2015, 54, 4187–4191. 10.1002/anie.201409602. PMC4409818 25973468

[ref17] Edwards W.; Marro N.; Turner G.; Kay E. R. Continuum Tuning of Nanoparticle Interfacial Properties by Dynamic Covalent Exchange. Chem. Sci. 2018, 9, 125–133. 10.1039/C7SC03666C. 29629080 PMC5869618

[ref18] Diez-Castellnou M.; Suo R.; Marro N.; Matthew S. A. L.; Kay E. R. Rapidly Adaptive All-Covalent Nanoparticle Surface Engineering. Chem. Eur. J. 2021, 27, 9948–9953. 10.1002/chem.202101042. 33871124 PMC8362155

[ref19] Algar W. R.; Krull U. J. Adsorption and Hybridization of Oligonucleotides on Mercaptoacetic Acid-Capped CdSe/ZnS Quantum Dots and Quantum Dot–Oligonucleotide Conjugates. Langmuir 2006, 22, 11346–11352. 10.1021/la062217y. 17154624

[ref20] Qiu X.; Hildebrandt N. Rapid and Multiplexed MicroRNA Diagnostic Assay Using Quantum Dot-Based Förster Resonance Energy Transfer. ACS Nano 2015, 9, 8449–8457. 10.1021/acsnano.5b03364. 26192765

[ref21] Pini F.; Francés-Soriano L.; Andrigo V.; Natile M. M.; Hildebrandt N. Optimizing Upconversion Nanoparticles for FRET Biosensing. ACS Nano 2023, 17, 4971–4984. 10.1021/acsnano.2c12523. 36867492

[ref22] Banerjee A.; Pons T.; Lequeux N.; Dubertret B. Quantum Dots–DNA Bioconjugates: Synthesis to Applications. Interface Focus 2016, 6, 20160064 10.1098/rsfs.2016.0064. 27920898 PMC5071820

[ref23] Schreiber C. L.; Smith B. D. Molecular Conjugation Using Non-Covalent Click Chemistry. Nat. Rev. Chem. 2019, 3, 393–400. 10.1038/s41570-019-0095-1. 33834115 PMC8025804

[ref24] Yu G.; Jie K.; Huang F. Supramolecular Amphiphiles Based on Host–Guest Molecular Recognition Motifs. Chem. Rev. 2015, 115, 7240–7303. 10.1021/cr5005315. 25716119

[ref25] Addonizio C. J.; Gates B. D.; Webber M. J. Supramolecular “Click Chemistry” for Targeting in the Body. Bioconjug. Chem. 2021, 32, 1935–1946. 10.1021/acs.bioconjchem.1c00326. 34415139 PMC8764705

[ref26] de laRica R.; Fratila R. M.; Szarpak A.; Huskens J.; Velders A. H. Multivalent Nanoparticle Networks as Ultrasensitive Enzyme Sensors. Angew. Chem. Int. Ed. 2011, 50, 5704–5707. 10.1002/anie.201008189. 21591033

[ref27] Fernández-Caro H.; Méndez-Ardoy A.; Montenegro J. Dynamic Nanosurface Reconfiguration by Host–Guest Supramolecular Interactions. Nanoscale 2022, 14, 3599–3608. 10.1039/D1NR05315A. 35188162

[ref28] Niehues M.; Engel S.; Ravoo B. J. Photo-Responsive Self-Assembly of Plasmonic Magnetic Janus Nanoparticles. Langmuir 2021, 37, 11123–11130. 10.1021/acs.langmuir.1c01979. 34499520

[ref29] Zhou Y.; Wang D.; Huang S.; Auernhammer G.; He Y.; Butt H.-J.; Wu S. Reversible Janus Particle Assembly via Responsive Host–Guest Interactions. Chem. Commun. 2015, 51, 2725–2727. 10.1039/C4CC09672J. 25574952

[ref30] Dsouza R. N.; Pischel U.; Nau W. M. Fluorescent Dyes and Their Supramolecular Host/Guest Complexes with Macrocycles in Aqueous Solution. Chem. Rev. 2011, 111, 7941–7980. 10.1021/cr200213s. 21981343

[ref31] Jin Jeon Y.; Kim S.-Y.; Ho Ko Y.; Sakamoto S.; Yamaguchi K.; Kim K. Novel Molecular Drug Carrier: Encapsulation of Oxaliplatin in Cucurbit[7]Uril and Its Effects on Stability and Reactivity of the Drug. Org. Biomol. Chem. 2005, 3, 2122–2125. 10.1039/b504487a. 15917899

[ref32] Assaf K. I.; Nau W. M. Cucurbiturils: From Synthesis to High-Affinity Binding and Catalysis. Chem. Soc. Rev. 2015, 44 (2), 394–418. 10.1039/C4CS00273C. 25317670

[ref33] Barrow S. J.; Kasera S.; Rowland M. J.; del Barrio J.; Scherman O. A. Cucurbituril-Based Molecular Recognition. Chem Rev 2015, 115, 12320–12406. 10.1021/acs.chemrev.5b00341. 26566008

[ref34] Huang Z.; Chen X.; O’Neill S. J. K.; Wu G.; Whitaker D. J.; Li J.; McCune J. A.; Scherman O. A. Highly Compressible Glass-like Supramolecular Polymer Networks. Nat Mater 2022, 21, 103–109. 10.1038/s41563-021-01124-x. 34819661

[ref35] Tan L.; Wei M.; Shang L.; Yang Y. Cucurbiturils-Mediated Noble Metal Nanoparticles for Applications in Sensing, SERS, Theranostics, and Catalysis. Adv. Funct. Mater. 2021, 31, 2007277 10.1002/adfm.202007277.

[ref36] Benyettou F.; Nchimi-Nono K.; Jouiad M.; Lalatonne Y.; Milosevic I.; Motte L.; Olsen J.-C.; Saleh N.; Trabolsi A. Viologen-Templated Arrays of Cucurbit[7]uril-Modified Iron-Oxide Nanoparticles. Chem. Eur. J. 2015, 21, 4607–4613. 10.1002/chem.201405774. 25582844

[ref37] Lee T.-C.; Scherman O. A. A Facile Synthesis of Dynamic Supramolecular Aggregates of Cucurbit[n]Uril (N=5-8) Capped with Gold Nanoparticles in Aqueous Media. Chem. Eur. J. 2012, 18, 1628–1633. 10.1002/chem.201102675. 22238182

[ref38] Han Y.; Yang X.; Liu Y.; Ai Q.; Liu S.; Sun C.; Liang F. Supramolecular Controlled Cargo Release via Near Infrared Tunable Cucurbit[7]Uril-Gold Nanostars. Sci. Rep. 2016, 6, 22239 10.1038/srep22239. 26917240 PMC4768098

[ref39] Taylor R. W.; Lee T.-C.; Scherman O. A.; Esteban R.; Aizpurua J.; Huang F. M.; Baumberg J. J.; Mahajan S. Precise Subnanometer Plasmonic Junctions for SERS within Gold Nanoparticle Assemblies Using Cucurbit[n]Uril “Glue.”. ACS Nano 2011, 5, 3878–3887. 10.1021/nn200250v. 21488693

[ref40] Kasera S.; Biedermann F.; Baumberg J. J.; Scherman O. A.; Mahajan S. Quantitative SERS Using the Sequestration of Small Molecules inside Precise Plasmonic Nanoconstructs. Nano Lett. 2012, 12, 5924–5928. 10.1021/nl303345z. 23088754

[ref41] Lu X.; Masson E. Formation and Stabilization of Silver Nanoparticles with Cucurbit[n]Urils (N= 5–8) and Cucurbituril-Based Pseudorotaxanes in Aqueous Medium. Langmuir 2011, 27, 3051–3058. 10.1021/la104729j. 21322592

[ref42] Chio W.-I. K.; Peveler W. J.; Assaf K. I.; Moorthy S.; Nau W. M.; Parkin I. P.; Olivo M.; Lee T.-C. Selective Detection of Nitroexplosives Using Molecular Recognition within Self-Assembled Plasmonic Nanojunctions. J. Phys. Chem. C 2019, 123, 15769–15776. 10.1021/acs.jpcc.9b02363. PMC6614880 31303905

[ref43] Zhang M.; Gong Z.; Yang W.; Jin L.; Liu S.; Chang S.; Liang F. Regulating Host-Guest Interactions between Cucurbit[7]Uril and Guests on Gold Surfaces for Rational Engineering of Gold Nanoparticles. ACS Appl. Nano Mater. 2020, 3, 4283–4291. 10.1021/acsanm.0c00435.

[ref44] Sinha S.; Das Saha N.; Sasmal R.; Joshi D.; Chandrasekhar S.; Bosco M. S.; Agasti S. S. Reversible Encapsulations and Stimuli-Responsive Biological Delivery from a Dynamically Assembled Cucurbit[7]Uril Host and Nanoparticle Guest Scaffold. J. Mater. Chem. B. 2018, 6, 7329–7334. 10.1039/C8TB01596A. 32226626 PMC7100906

[ref45] Cui S.-C.; Tachikawa T.; Fujitsuka M.; Majima T. Photoinduced Electron Transfer in a Quantum Dot-Cucurbituril Supramolecular Complex. J. Phys. Chem. C 2011, 115, 1824–1830. 10.1021/jp1110828.

[ref46] Peveler W. J.; Jia H.; Jeen T.; Rees K.; Macdonald T. J.; Xia Z.; Chio W.-I. K.; Moorthy S.; Parkin I. P.; Carmalt C. J.; et al. Cucurbituril-Mediated Quantum Dot Aggregates Formed by Aqueous Self-Assembly for Sensing Applications. Chem. Commun. 2019, 55, 5495–5498. 10.1039/C9CC00410F. 31017133

[ref47] Sokołowski K.; Huang J.; Földes T.; McCune J. A.; Xu D. D.; de Nijs B.; Chikkaraddy R.; Collins S. M.; Rosta E.; Baumberg J. J.; Scherman O. A. Nanoparticle Surfactants for Kinetically Arrested Photoactive Assemblies to Track Light-Induced Electron Transfer. Nat. Nanotechnol. 2021, 16, 1121–1129. 10.1038/s41565-021-00949-6. 34475556

[ref48] Peveler W. J.; Algar W. R. More Than a Light Switch: Engineering Unconventional Fluorescent Configurations for Biological Sensing. ACS Chem. Biol. 2018, 13, 1752–1766. 10.1021/acschembio.7b01022. 29461796

[ref49] Yu W. W.; Qu L.; Guo W.; Peng X. Experimental Determination of the Extinction Coefficient of CdTe, CdSe, and CdS Nanocrystals. Chem. Mater. 2003, 15, 2854–2860. 10.1021/cm034081k.

[ref50] Cohen E.; Gdor I.; Romero E.; Yochelis S.; van Grondelle R.; Paltiel Y. Achieving Exciton Delocalization in Quantum Dot Aggregates Using Organic Linker Molecules. J. Phys. Chem. Lett. 2017, 8, 1014–1018. 10.1021/acs.jpclett.6b02980. 28195481

[ref51] Kagan C. R.; Murray C. B.; Nirmal M.; Bawendi M. G. Electronic Energy Transfer in CdSe Quantum Dot Solids. Phys. Rev. Lett. 1996, 76, 1517–1520. 10.1103/PhysRevLett.76.1517. 10061743

[ref52] Crooker S. A.; Hollingsworth J. A.; Tretiak S.; Klimov V. I. Spectrally Resolved Dynamics of Energy Transfer in Quantum-Dot Assemblies: Towards Engineered Energy Flows in Artificial Materials. Phys. Rev. Lett. 2002, 89, 186802 10.1103/PhysRevLett.89.186802. 12398626

[ref53] Gnidovec A.; Božič A.; Čopar S. Dense Packings of Geodesic Hard Ellipses on a Sphere. Soft Matter 2022, 18, 7670–7678. 10.1039/D2SM00624C. 36172841

[ref54] Moghaddam S.; Yang C.; Rekharsky M.; Ko Y. H.; Kim K.; Inoue Y.; Gilson M. K. New Ultrahigh Affinity Host–Guest Complexes of Cucurbit[7]Uril with Bicyclo[2.2.2]Octane and Adamantane Guests: Thermodynamic Analysis and Evaluation of M2 Affinity Calculations. J. Am. Chem. Soc. 2011, 133, 3570–3581. 10.1021/ja109904u. 21341773 PMC3065999

[ref55] Alnajjar M. A.; Nau W. M.; Hennig A. A Reference Scale of Cucurbit[7]Uril Binding Affinities. Org. Biomol. Chem. 2021, 19, 8521–8529. 10.1039/D1OB01304A. 34378628

[ref56] Le Trong I.; Wang Z.; Hyre D. E.; Lybrand T. P.; Stayton P. S.; Stenkamp R. E. Streptavidin and Its Biotin Complex at Atomic Resolution. Acta Crystallogr Sect D Biological Crystallogr 2011, 67, 813–821. 10.1107/S0907444911027806. PMC3169315 21904034

[ref57] Liu Y.; Miao K.; Dunham N. P.; Liu H.; Fares M.; Boal A. K.; Li X.; Zhang X. The Cation−π Interaction Enables a Halo-Tag Fluorogenic Probe for Fast No-Wash Live Cell Imaging and Gel-Free Protein Quantification. Biochemistry 2017, 56 (11), 1585–1595. 10.1021/acs.biochem.7b00056. 28221782 PMC5362743

[ref58] Shu X.; Shaner N. C.; Yarbrough C. A.; Tsien R. Y.; Remington S. J. Novel Chromophores and Buried Charges Control Color in MFruits. Biochemistry 2006, 45 (32), 9639–9647. 10.1021/bi060773l. 16893165

[ref59] Sehnal D.; Bittrich S.; Deshpande M.; Svobodová R.; Berka K.; Bazgier V.; Velankar S.; Burley S. K.; Koča J.; Rose A. S. Mol* Viewer: Modern Web App for 3D Visualization and Analysis of Large Biomolecular Structures. Nucleic Acids Res. 2021, 49, W431–W437. 10.1093/nar/gkab314. 33956157 PMC8262734

[ref60] Jain A.; Cheng K. The Principles and Applications of Avidin-Based Nanoparticles in Drug Delivery and Diagnosis. J. Control. Release 2017, 245, 27–40. 10.1016/j.jconrel.2016.11.016. 27865853 PMC5222781

[ref61] Schwarz D. H.; Elgaher W. A. M.; Hollemeyer K.; Hirsch A. K. H.; Wenz G. Reversible Immobilization of a Protein to a Gold Surface through Multiple Host–Guest Interactions. J. Mater. Chem. B 2019, 7 (40), 6148–6155. 10.1039/C9TB00560A. 31555792

[ref62] Los G. V.; Encell L. P.; McDougall M. G.; Hartzell D. D.; Karassina N.; Zimprich C.; Wood M. G.; Learish R.; Ohana R. F.; Urh M.; et al. HaloTag: A Novel Protein Labeling Technology for Cell Imaging and Protein Analysis. ACS Chem. Biol. 2008, 3 (6), 373–382. 10.1021/cb800025k. 18533659

[ref63] Senler S.; Cheng B.; Kaifer A. E. Rotaxane Formation by Cucurbit[7]Uril in Water and DMSO Solutions. Org. Lett. 2014, 16 (22), 5834–5837. 10.1021/ol502479k. 25383988

[ref64] Zhan N.; Palui G.; Safi M.; Ji X.; Mattoussi H. Multidentate Zwitterionic Ligands Provide Compact and Highly Biocompatible Quantum Dots. J. Am. Chem. Soc. 2013, 135 (37), 13786–13795. 10.1021/ja405010v. 24003892

[ref65] Klajn R.; Olson M. A.; Wesson P. J.; Fang L.; Coskun A.; Trabolsi A.; Soh S.; Stoddart J. F.; Grzybowski B. A. Dynamic Hook-and-Eye Nanoparticle Sponges. Nat. Chem. 2009, 1 (9), 733–738. 10.1038/nchem.432. 21124361

[ref66] Palankar R.; Medvedev N.; Rong A.; Delcea M. Fabrication of Quantum Dot Microarrays Using Electron Beam Lithography for Applications in Analyte Sensing and Cellular Dynamics. ACS Nano 2013, 7 (5), 4617–4628. 10.1021/nn401424y. 23597071

[ref67] Zou L.; Braegelman A. S.; Webber M. J. Dynamic Supramolecular Hydrogels Spanning an Unprecedented Range of Host–Guest Affinity. ACS Appl. Mater. Interfaces 2019, 11 (6), 5695–5700. 10.1021/acsami.8b22151. 30707553

[ref68] Zou L.; Braegelman A. S.; Webber M. J. Spatially Defined Drug Targeting by in Situ Host–Guest Chemistry in a Living Animal. ACS Cent. Sci. 2019, 5 (6), 1035–1043. 10.1021/acscentsci.9b00195. 31263763 PMC6598162

[ref69] Day A.; Arnold A. P.; Blanch R. J.; Snushall B. Controlling Factors in the Synthesis of Cucurbituril and Its Homologues. J. Org. Chem. 2001, 66 (24), 8094–8100. 10.1021/jo015897c. 11722210

[ref70] Kim J.; Jung I.-S.; Kim S.-Y.; Lee E.; Kang J.-K.; Sakamoto S.; Yamaguchi K.; Kim K. New Cucurbituril Homologues: Syntheses, Isolation, Characterization, and X-Ray Crystal Structures of Cucurbit[n]Uril (N= 5, 7, and 8). J. Am. Chem. Soc. 2000, 122 (3), 540–541. 10.1021/ja993376p.

[ref71] Tran D. P.; Macdonald T. J.; Wolfrum B.; Stockmann R.; Nann T.; Offenhäusser A.; Thierry B. Photoresponsive Properties of Ultrathin Silicon Nanowires. Appl. Phys. Lett. 2014, 105 (23), 231116 10.1063/1.4904089.

[ref72] Gustafson J. L.; Neklesa T. K.; Cox C. S.; Roth A. G.; Buckley D. L.; Tae H. S.; Sundberg T. B.; Stagg D. B.; Hines J.; McDonnell D. P.; et al. Small-Molecule-Mediated Degradation of the Androgen Receptor through Hydrophobic Tagging. Angew. Chem., Int. Ed. 2015, 54 (33), 9659–9662. 10.1002/anie.201503720. PMC4547777 26083457

