# Supplementary material for: Supramolecular Click Chemistry for Surface Modification of Quantum Dots Mediated by Cucurbit[7]uril
Source: ACS Nano. 2023 Nov 3;17(21):21585–94. doi: 10.1021/acsnano.3c06601 (PMC10655248; doi:10.1021/acsnano.3c06601)
Supplement: Supplementary file 1 — nn3c06601_si_001.pdf [file nn3c06601_si_001.pdf]

Supporting Information

Supramolecular Click Chemistry for Surface Modification of Quantum Dots Mediated by Cucurbit[7]uril

Katie McGuire[1], Suhang He[2], Jennifer Gracie[1], Charlotte Bryson[1], Dazhong Zheng[1], Alasdair W Clark[3], Jesko Koehnke[1,4], David J France[1], Werner M Nau[2], Tung-Chun Lee[5,6] and William J Peveler[1,*]

1) School of Chemistry, Joseph Black Building, University of Glasgow, Glasgow, G12 8QQ, UK
E-mail: william.peveler@glasgow.ac.uk

2) School of Science, Constructor University, Campus Ring 1, 28759 Bremen, Germany

3) James Watt School of Engineering, Advanced Research Centre, University of Glasgow, Glasgow, G11 6EW, UK

4) Institut für Lebensmittelchemie, Leibniz Universität Hannover, Callinstr 5, 30167 Hannover, Germany

5) Institute for Materials Discovery, University College London, London, WC1H 0AJ, UK

6) Department of Chemistry, University College London, London, WC1H 0AJ, UK

Table of Contents

Figure S1 – Transmission Electron Microscopy of CdTe530 QDs 3
Figure S2 – Dynamic Light Scattering of CdTe530 QDs 3
Figure S3 – Optical characterisation of CdTe530, CdTe540 and CdSe/ZnS514 QDs 4
Table S1 – Size calculation and absorption coefficient estimation for CdTe530 4
Table S2 – Surface area coverage estimations for CdTe530 4
Figure S4 – DFT model of A3OH•CB7 and solution NMR 5
Figure S5 – Fluorescence displacement titration to estimate A3OH affinity for CB7 6
Figure S6 – Additional DLS of A3OH and A8OH host/guest systems with CdTe530 7
Figure S7 – Control titration of triethylene glycol into CB7-aggregated CdTe530 7
Figure S8 – Images of CB7-aggregated and disaggregated CdTe530 with A3OH 8
Figure S9 – Gel electrophoresis to monitor the CB7 mediated aggregation and disaggregation process of CdTe530 8
Figure S10 – Aggregation and disaggregation of CdTe540 with CB7 9
Figure S11 – Monitoring CB7-mediated aggregation and disaggregation of CdSe/ZnS514 by DLS 9
Figure S12 – Additional FRET measurements on CB7-aggregated CdTe530 with A3OH and A3Cy3.5 10
Figure S13 – NMR of A3biotin and CB7 11
Figure S14 – Absorbance and emission spectra for CdTe530 and Alexa568 labelled streptavidin (SAv-Alexa568) 11
Figure S15 – DLS of A3biotin host/guest systems on CdTe530 with Neutravidin 12
Figure S16 – NMR titration of A4Halo into CB7 12
Figure S17 – Association of A4Halo with CdTe540•CB7 aggregates 13
Figure S18 – Absorbance and emission spectra for Halo-RFP and CdTe540 14
Figure S19 – Additional DFT models of quaternary ammonium-containing guests 14
Figure S20 – NMR of Py3OMe•CB7, Ap3OMe•CB7 and Im3OMe•CB7 15
Figure S21 – Isothermal calorimetry for Py3OMe, Ap3OMe and Im3OMe with CB7 17
Figure S21 – Additional DLS measures of Py16OMe, Ap16OMe and Im16OMe disrupting CdTe530/CB7 aggregates 19
General Materials and Methods 20
DFT Methods 20
Instrumentation and Measures 20
Synthesis of Nanoparticles 21
Production of RFP 22
Synthesis and Characterisation of Molecular Guests 23
References 42

Figure S1 – Transmission Electron Microscopy of CdTe530 QDs

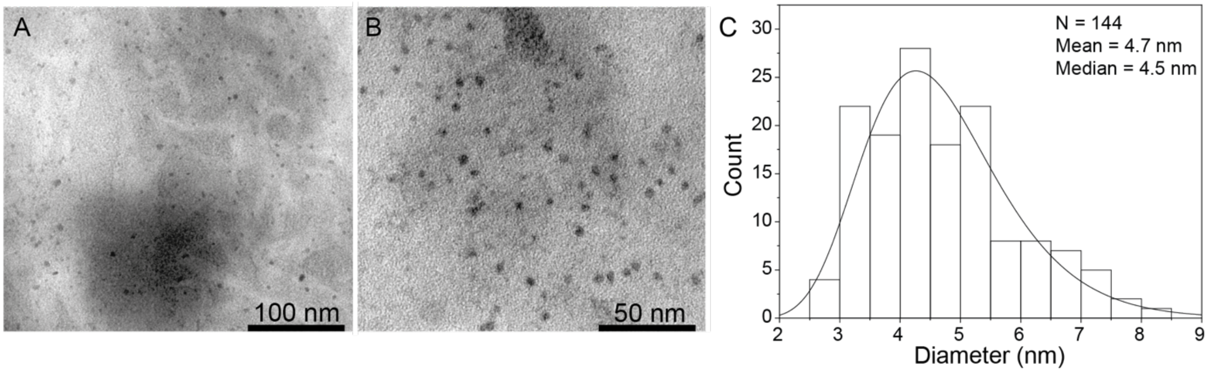

Representative TEM images of CdTe530 QDs (a/b) and (c) a histogram of particle size counted with automated threshold and edge finding routines in ImageJ. A lognormal distribution is shown. Standard deviation on the mean is ±1.2 nm. It should be noted that at this low resolution/magnification, particles may appear larger than they are due to poorly defined edges, and may also have defected material at the surface, meaning that the actual QD size is smaller than the observed particle size. This is supported by DLS measures (below) which suggest a particle size of less than 4 nm.

Figure S2 – Dynamic Light Scattering of CdTe530 QDs

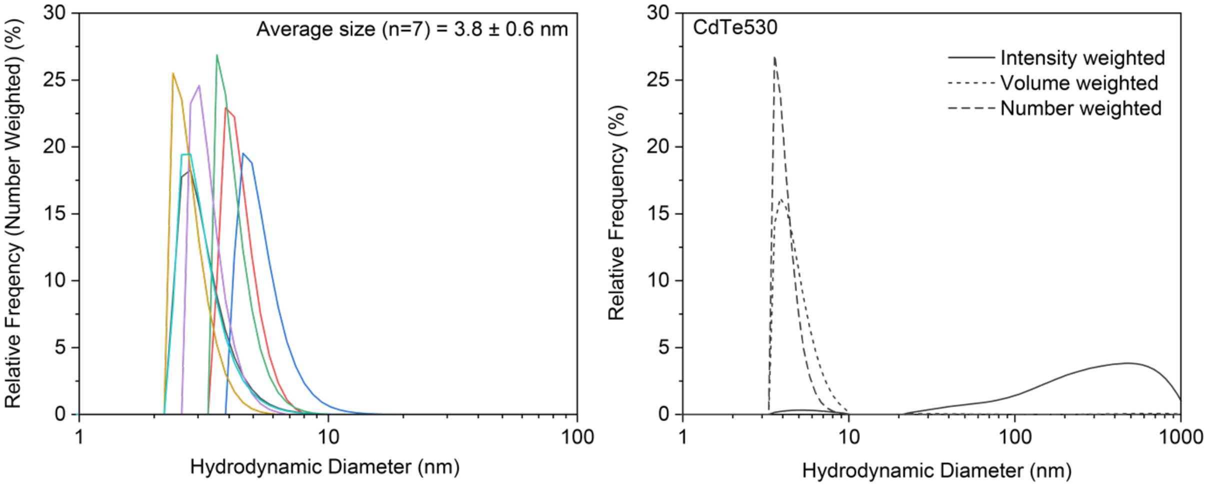

(Left) Number weighted DLS data for seven independent measurements on the batches of CdTe530 used in this work. The overall average size is 3.8 ± 0.6 nm (number weighted) or 4.5 ± 1.0 nm if the volume weighted data is used. (Right) Illustration of the difference in intensity, volume, and number weighted DLS for one sample. Number or volume weighting correction is necessary for the small size of the nanoparticles and greatly increased scattering from larger particles. These corrections were applied by the instrument software on the basis of the measured correlation coefficients, solvent refractive index, solid refractive index and absorption coefficient for CdTe at the 633 nm laser wavelength used (see also Figure S6 below).

Figure S3 – Optical characterisation of CdTe530, CdTe540 and CdSe/ZnS514 QDs

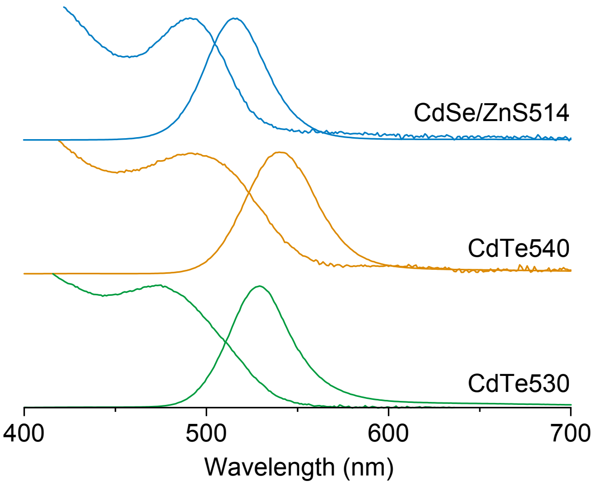

Normalised absorption and emission spectra for the QD samples used in this study. Emission spectra were collected with excitation at 375 nm, typical for the experiments.

Table S1 – Size calculation and absorption coefficient estimation for CdTe530

Using the methods of Peng et al.[1] and measures of QD absorption, a value for the molar extinction coefficient was estimated for the QD samples to enable normalisation between batches. A sample calculation is provided below:

Absorbance maximum (nm)	A at maximum (path length in cm)	D estimated (nm)	Estimated ϵ ($M^{-1}cm^{-1}$)	Estimated well concentration (µM)
475 nm	0.2 (0.86)	1.5	24,600	9.5 uM

This can only ever be an approximation due to the different synthetic methodologies used. Organic 'hot-injection' particles that are used for the literature calibration, whilst our QDs are produced with an aqueous 'heat up' method. An alternative approximation of ϵ can be made using the CdTe(aq) calibration proposed by Dong and Ren.[2] In our case we found our estimated ϵ to be broadly consistent with the green emitting QDs measured in this work (24,000-26,000 $M^{-1}cm^{-1}$), however our measured particle size was larger for the same emission wavelength (reported 2.5 nm), consistent with the discussion above.

Table S2 – Surface area coverage estimations for CdTe530

The packing on cylinders on the surface of a sphere (a geometric approximation for this system) is non-trivial, as packing density varies depending on the relative size (curvature) of the sphere and the diameter of the cylinder. As an estimate, using circles on a spherical plane, Gnidovec et al. give a packing efficiency of 0.84,[3] so we use this to model the number of whole CB7 (circular footprint of 2 nm^2) that may fit on various size spheres.

QD diameter (nm)	QD surface area (nm^2)	CB7 footprint (nm^2)	Packing density	CB/QD
4	50	2	0.84	21
3.5	38	2	0.84	16
3	28	2	0.84	11
2.5	20	2	0.84	8

Figure S4 – DFT model of A3OH•CB7 and solution NMR

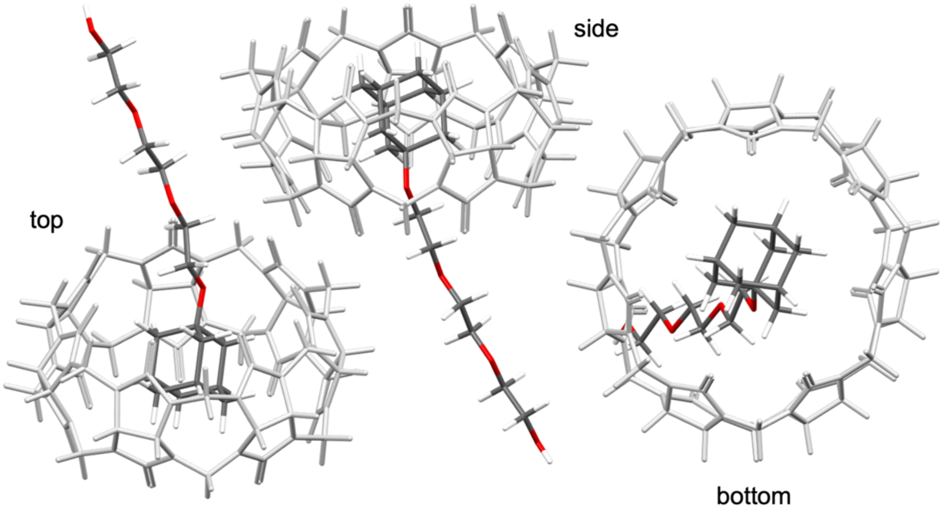

A DFT model of **A3OH** in the CB7 cavity (above) shows a good fit, differential interaction of the internal and external protons of the top face with the PEG chain, and no projection from the bottom face. Solution ^{1}H NMR (below, D_2O, 400 MHz) shows the downfield 'int' proton doublet has a large splitting, whilst the 'ext' proton doublet shows only a small splitting, resulting from inequivalence at each portal. The equatorial proton singlet shows only a small upfield shift. The adamantyl peaks shift greatly upfield due to increased shielding in the CB cavity, and there is a further loss of equivalency in the triethylene glycol chain, due to interactions with the portal protons.

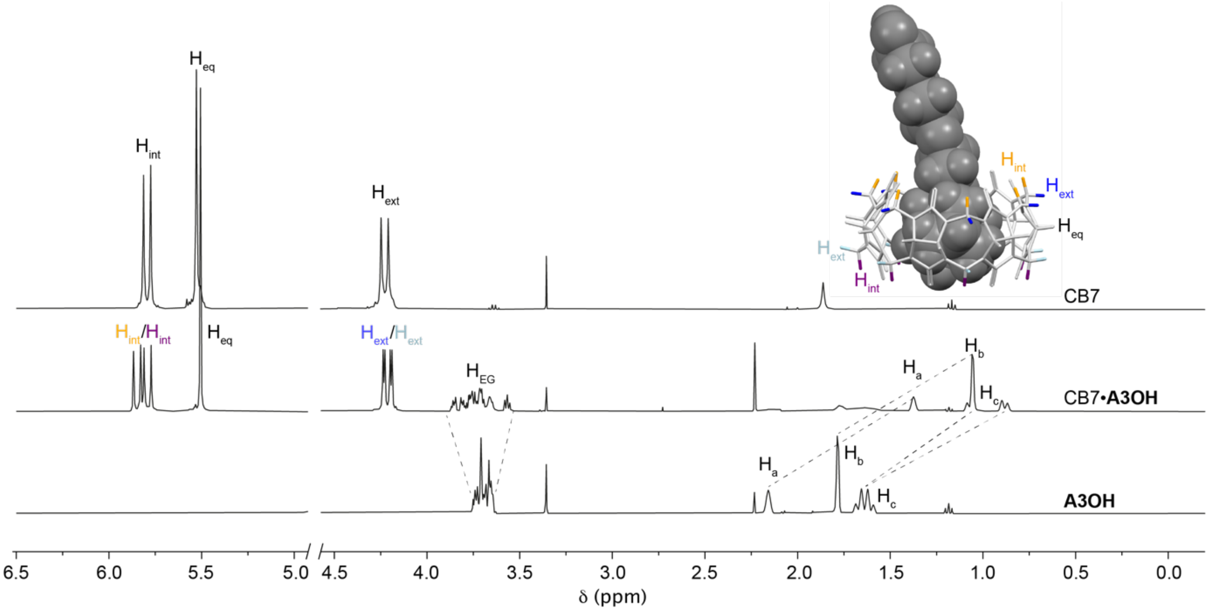

Figure S5 – Fluorescence displacement titration to estimate A3OH affinity for CB7

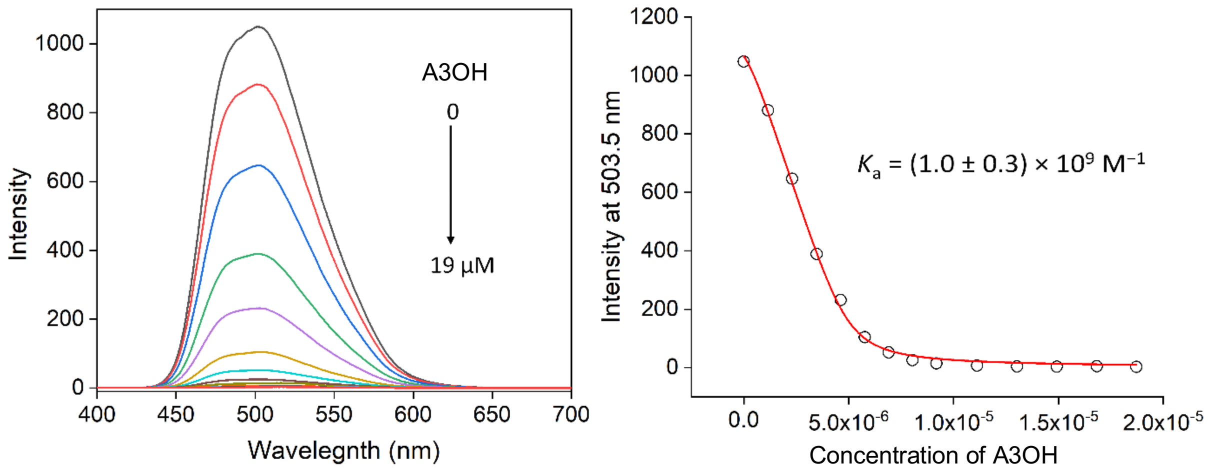

Competitive fluorescence titrations. Left: Fluorescence spectra of berberine (5 μM) and CB7 (5 μM) with increasing concentration of **A3OH** from 0 to 19 μM. Right: Plotting of the fluorescence intensity at 503.5 nm against the concentration of **A3OH**. The curve was fitted with a competitive binding model and the binding affinity of **A3OH**•CB7 was obtained to be (1.0 ± 0.3) × 10^9 M^{-1}.

The binding constant of **A3OH**•CB7 was determined through competitive fluorescence titrations, with berberine as the guest competitor.[4] To carry out the titration, 2.5 ml of the mixture solution containing 5 μM berberine and 5 μM CB7 was transferred to a fluorescence cuvette and measured as the starting point. Then, the solution was titrated with **A3OH** stock solution (1 mM) until its concentration reached around 19 μM. The fluorescence spectra of the solution in the range of 400-700 nm were measured after each addition, with an excitation wavelength of 342 nm. The fluorescence intensity at 503.5 nm of each spectrum was plotted against the concentration of **A3OH** and fitted with a competitive titration model with the Origin software package.

Figure S6 – Additional DLS of A3OH and A8OH host/guest systems with CdTe530

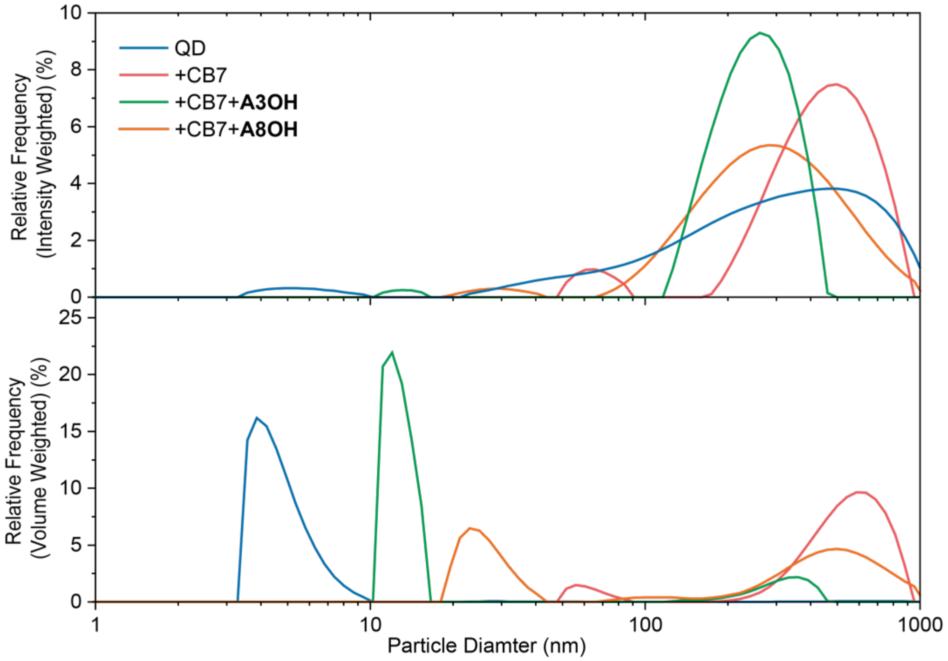

Representative, raw intensity weighted data (top), and volume weighted data (bottom) of the native CdTe530 QDs, after aggregation with CB7, and then subsequent disaggregation with either **A3OH** or **A8OH**. Due to the small size of the nanoscopic system, and the greatly increased scattering from larger particles, it was necessary to apply reasonable volumetric or number (in main text Figure 2) corrections, to better see the aggregates formed and broken up. These were applied by the instrument software on the basis of the measured correlation coefficients, solvent refractive index, solid refractive index and absorption coefficient for CdTe at the 633 nm laser wavelength used.

Even in the raw intensity weighted data it is obvious that the QDs aggregate and then disaggregate with a larger size than the raw QDs on application of the molecular guests.

Figure S7 – Control titration of triethylene glycol into CB7-aggregated CdTe530

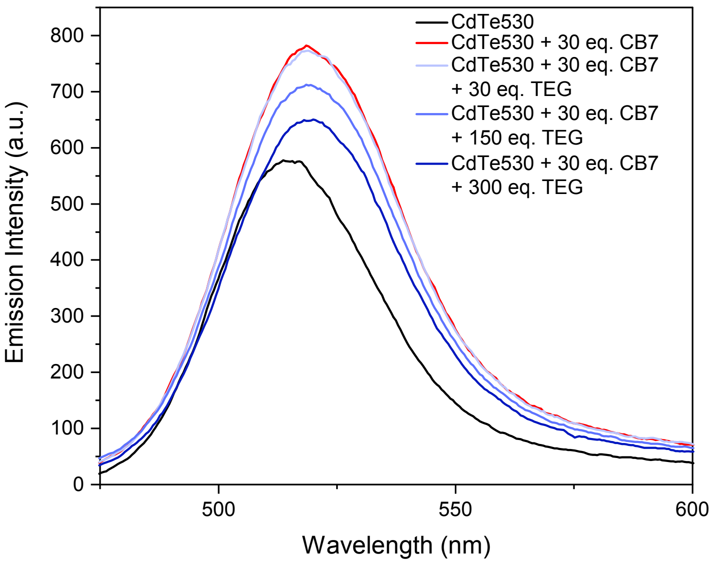

CdTe530 was aggregated with 30 eq. CB7 (red shifting observed between the black and red curves) and then titrated with either 30, 150 or 300 eq. triethyelene glycol (TEG) per QD (blue curves). No

evidence of disaggregation was observed (no blue shifting), although the change in solution refractive index or a general dilution effect did modify the luminescence intensity slightly.

Figure S8 – Images of CB7-aggregated and disaggregated CdTe530 with A3OH

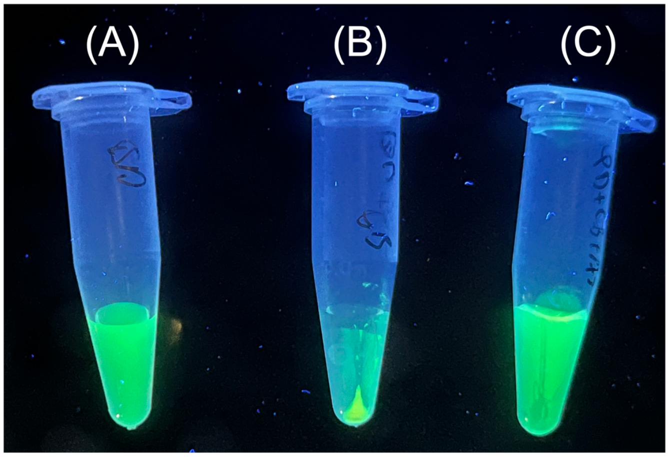

Images of (A) CdTe530, (B) CdTe530 aggregated with 30 eq. CB7 and then centrifuged briefly to pellet the aggregates, (C) CdTe530 aggregated with 30 eq. CB7, and then mixed with 30 eq. A3OH before centrifugation, showing the non-aggregated QDs are resistant to centrifugation. Samples imaged under 365 nm UV light.

Figure S9 – Gel electrophoresis to monitor the CB7 mediated aggregation and disaggregation process of CdTe530

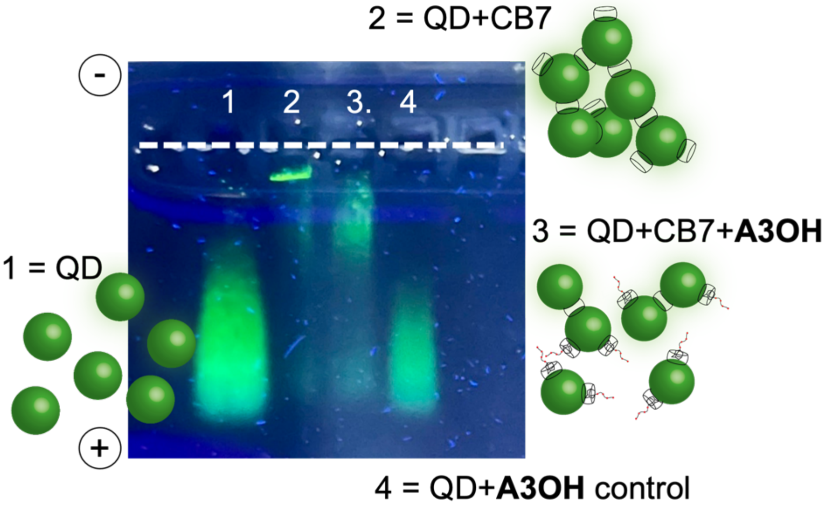

Agarose gel electrophoresis of native CdTe530 QDs (Lane 1), CdTe aggregated with 30 eq. CB7 (Lane 2), QD/CB aggregates treated with 30 eq. **A3OH** (Lane 3), and a control of the native QDs with **A3OH** alone (Lane 4). Gel is produced using 1% agarose in 1X TAE buffer, and wells are loaded with *c.* 4 uL solution and 1 uL glycerol per well (40 pmol QDs per well) and run at 100 V (16.7 V cm^{-1}) for 30 minutes.

Figure S10 – Aggregation and disaggregation of CdTe540 with CB7

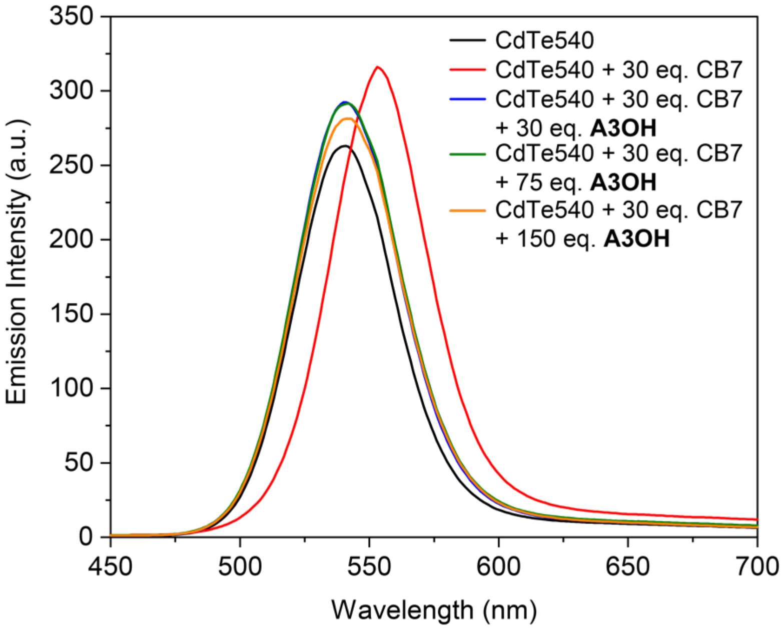

CdTe540 (10 μM) was measured (excitation at 375 nm) and then aggregated with 30 eq. CB7, and after subsequent disaggregation with between 30 and 150 eq. **A3OH** per quantum dot (1-5 eq. **A3OH** per CB7). A larger red shift was observed on aggregation than for the greener CdTe530, due to the larger population distribution in the larger QDs (FWHM was measured at 46 nm, vs 40 nm for CdTe530).

Figure S11 – Monitoring CB7-mediated aggregation and disaggregation of CdSe/ZnS514 by DLS

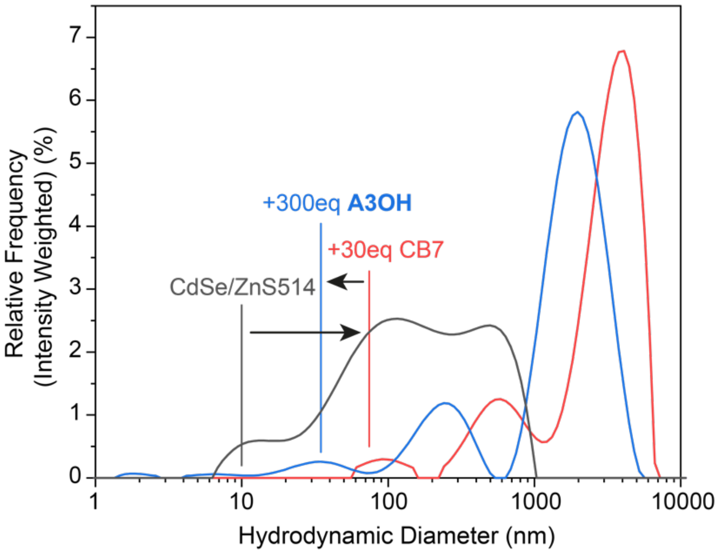

CdSe/ZnS514 (10 μM) was measured by DLS – the smaller size of the particles and poorer scattering meant that number weighted data was variable, so uncorrected intensity weighted data is presented here, but is skewed by larger aggregates. The aggregation and disaggregation process still worked as hypothesised but was less effective – aggregation was slower due to the GSH surface coating rather than MPA on the CdTe samples, and it appeared more equivalents of **A3OH** were required to disaggregate.

Figure S12 – Additional FRET measurements on CB7-aggregated CdTe530 with A3OH and A3Cy3.5

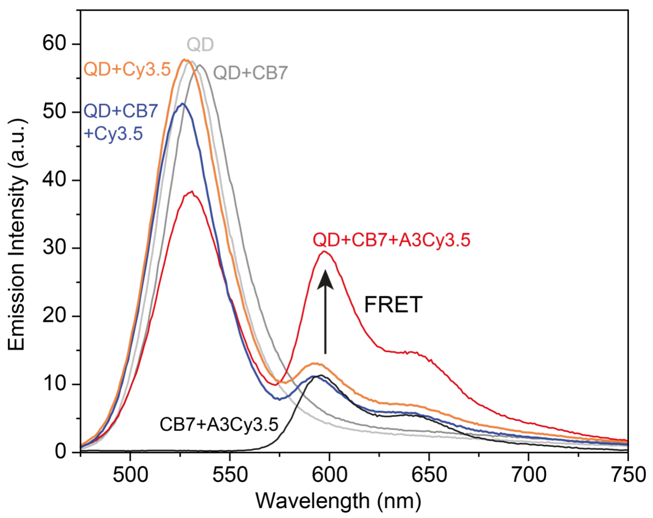

Additional control experiments on aggregated (30 eq. CB7) and unaggregated CdTe530 with sulfo-Cy3.5 without an adamantyl label (pendent carboxylic acid present). FRET is only observed in the complete system and there is limited non-specific adsorption onto the QD surface.

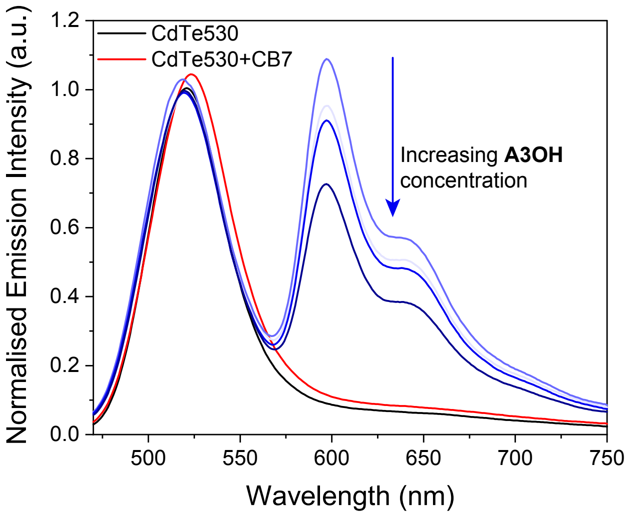

CdTe530 was aggregated with 30 eq. CB7 and 30 eq. **A3Cy3.5**. This was then titrated with 10–100 eq. of **A3OH** per **A3Cy3.5** (300-3000 eq. per QD). Data are normalised in intensity to the QD peak to show the loss of Cy3.5 intensity as a result in FRET reduction on addition of the unlabelled guest. The large excess is required due to the similar and high binding affinities of the guests and slow dissociation kinetics.

Figure S13 – NMR of A3biotin and CB7

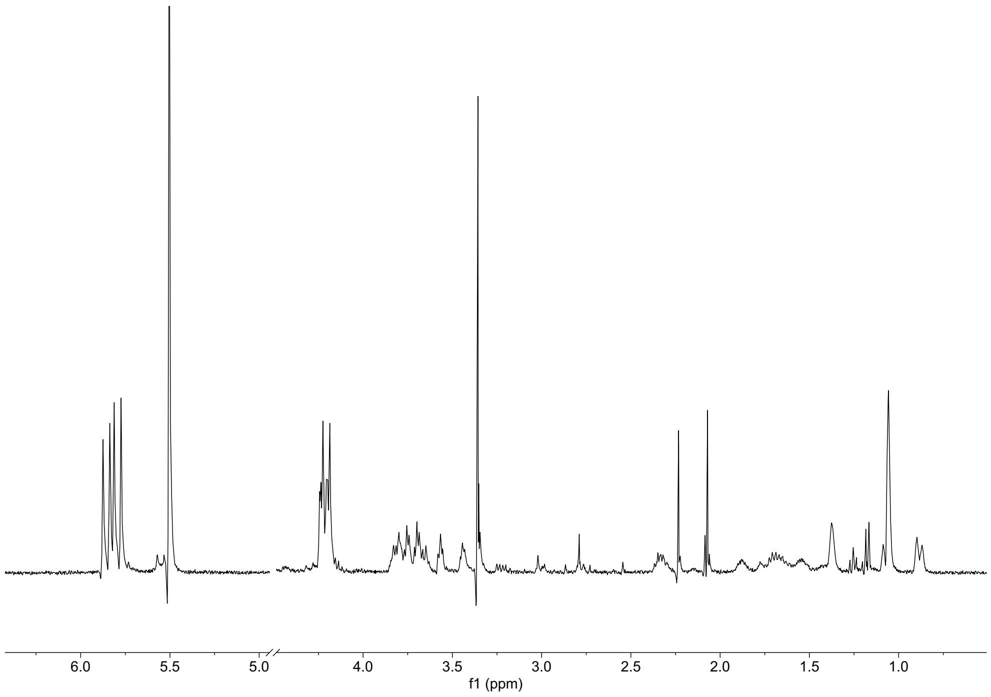

NMR of **A3biotin** with CB7 in D_2O (residual HOD peak removed for clarity). The CB7 peaks (4.0-6.0 ppm) are split and shifted as expected in the slow exchange regime, and as observed in the similar **A3OH** experiment the PEG protons (3.5-4.0 ppm) are split further, due to increased loss of symmetry, and the adamantyl peaks (1.5-2.5 ppm in uncomplexed molecule) are clearly shifted upfield below 1.5 ppm.

Figure S14 – Absorbance and emission spectra for CdTe530 and Alexa568 labelled streptavidin (SAv-Alexa568)

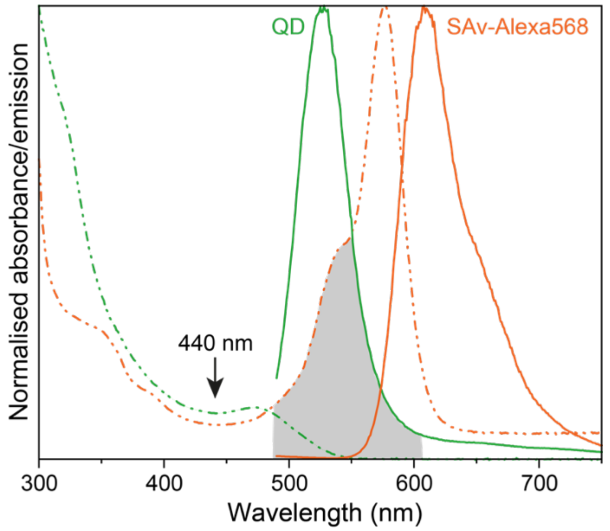

Absorbance data are in dash/dot lines, and emission solid lines. The potential FRET overlap is highlighted in grey. The system was excited at 440 nm to minimise direct excitation of the Alexa568 label, but there is no point with a true zero absorption, so some direct excitation always occurs.

Figure S15 – DLS of A3biotin host/guest systems on CdTe530 with Neutravidin

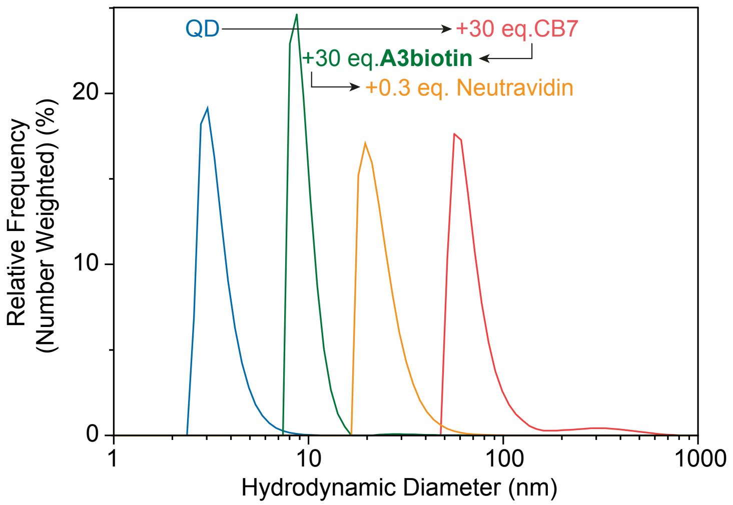

Monitoring the aggregation of CdTe530 with DLS shows the usual trend in size increase, and then subsequent decrease on addition of **A3biotin**. If non-fluorescent Neutravidin is then added (0.01 eq. per biotin) then a size increase is observed. The low ratio was used to prevent uncontrolled aggregation of the biotinylated QDs by the tetravalent Neutravidin, that would destabilise the system. Neutravidin is reported to be c. 6 nm in size,[5] so the > 10 nm increase in size here may suggest some mild aggregation of the system does occur.

Figure S16 – NMR titration of A4Halo into CB7

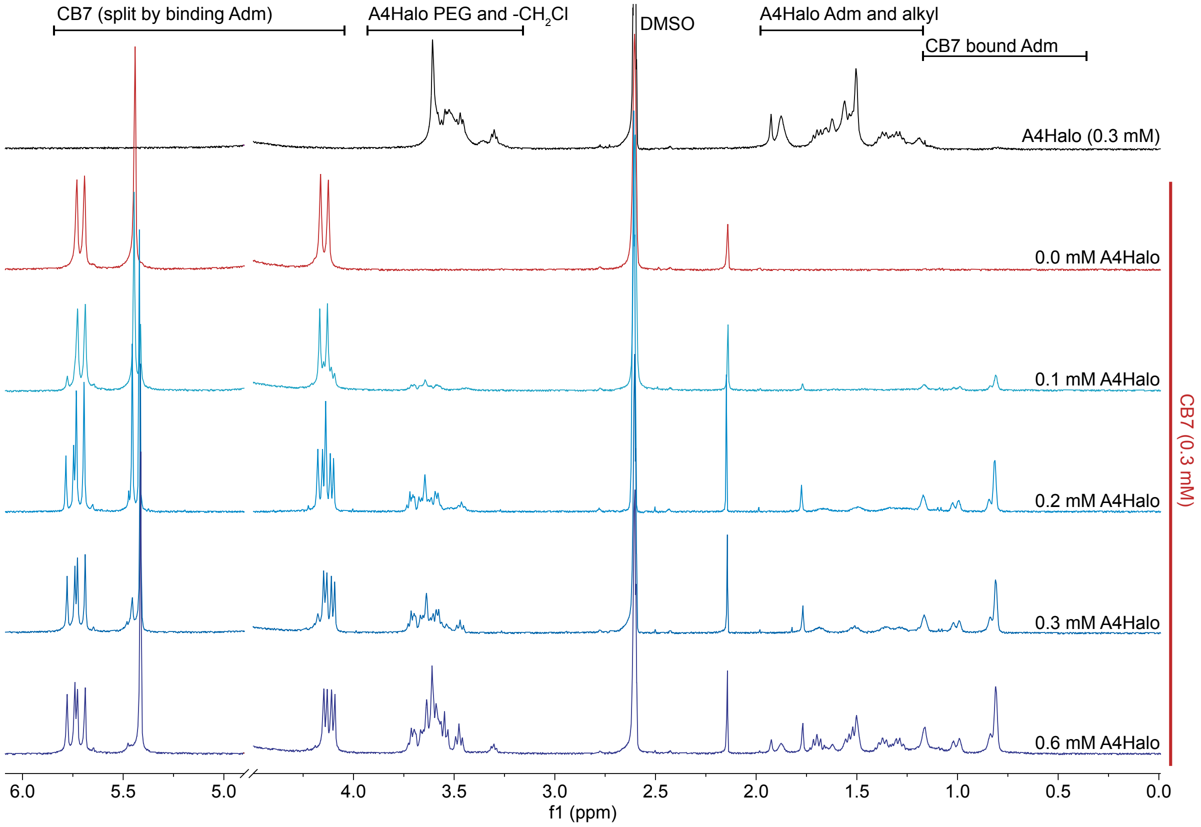

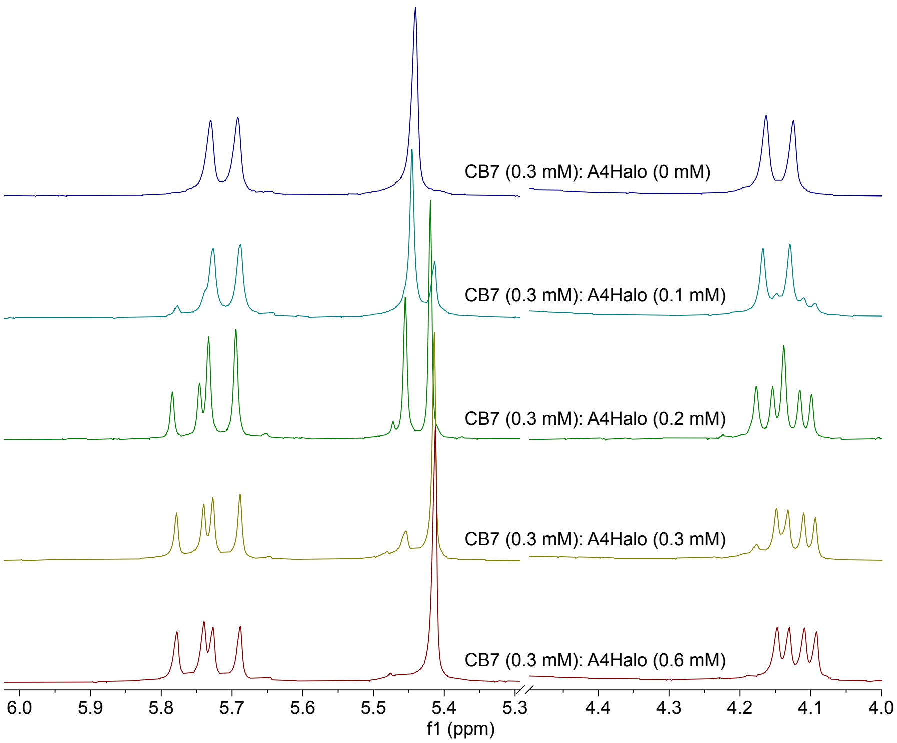

^{1}H NMR (400 MHz) of a titration of **A4Halo** into CB7 in D_2O (5% d6DMSO) (Top). CB7 is held at 0.3 mM and A4Halo is varied between 0.1 and 0.6 mM. The relative integral at 5.41 (bound CB7 H_{eq}) vs 5.45 (free CB7 H_{eq}) is used to quantify K_a on the basis of slow exchange on the NMR timescale (zoom-bottom), and averaged across the 4 experiments K_a is found to be $(4.1 \pm 0.6) \times 10^4$ M^{-1}.

It is noted that the recorded affinity for **A4Halo** is lower than expected for a neutral adamantyl guest in CB7. A survey by Scherman *et al.* suggests values of 10^8–10^9 M^{-1} might be thought typical for adamantyl moieties where there is not a positive amine group with good spacing from the hydrophobic adamantyl to interact with the CB portal.[6] We hypothesise that in the case of the adamantyl acetamide, there is no positive charge or well-spaced H-bond donor, and the carbonyl may indeed cause a steric hindrance to the binding. Furthermore, the inclusion of DMSO for solubilising the guest can also lower the apparent affinity by up to 5 orders of magnitude.[7] The same effect has been recently reported by Masson, Kaleta *et al.*[8] Thus, we posit that values in the region of 10^4-10^5 M^{-1} seem reasonable for an adamantyl with steric hindrance and in the presence of DMSO.

Figure S17 – Association of A4Halo with CdTe540•CB7 aggregates

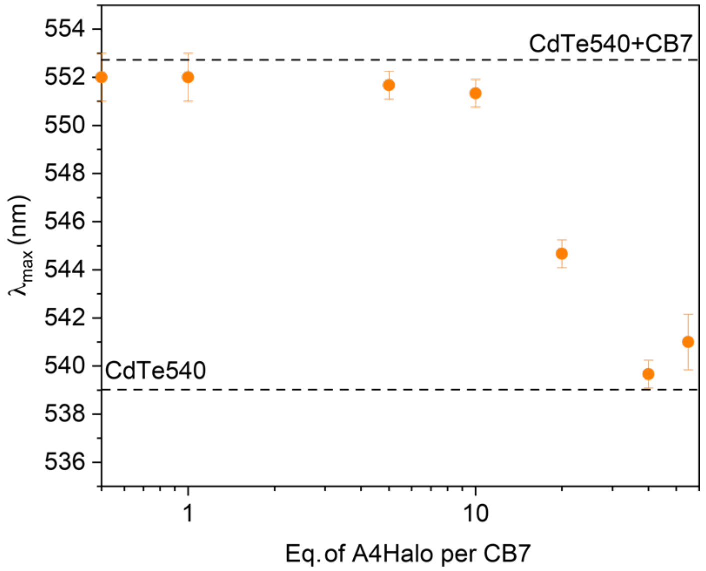

CdTe540 was aggregated with 30 eq. CB7, and then varying equivalencies of **A4Halo** per CB7 were added, and blue-shifting of the aggregate emission was monitored. An effect was seen then > 10 eq. per CB7 were added indicating a much lower affinity for the CB7 than seen with other AnY derivatives. This is likely because of the amide linkage used to impart solubility, rather than the ether linkage on other A*n*Y derivatives (see above).

Figure S18 – Absorbance and emission spectra for Halo-RFP and CdTe540

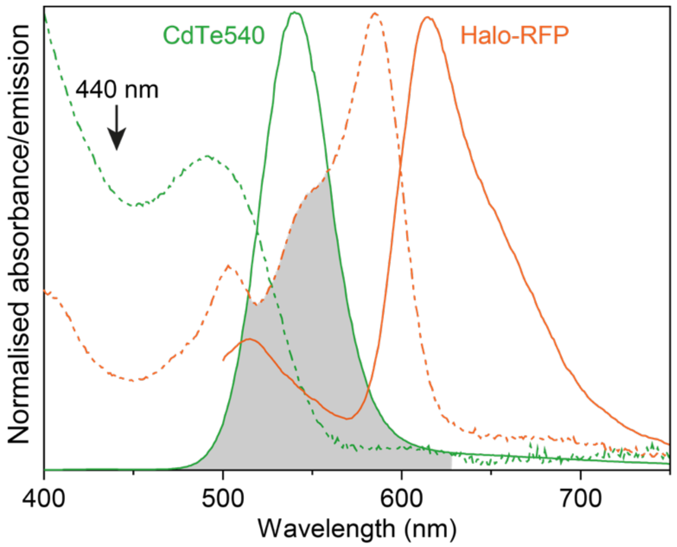

Absorbance data are in dash/dot lines, and emission solid lines. The potential FRET overlap is highlighted in grey. The system was excited at 440 nm to minimise direct excitation of the RFP, but there is no point with a true zero absorption, so some direct excitation always occurs.

Figure S19 – Additional DFT models of quaternary ammonium-containing guests

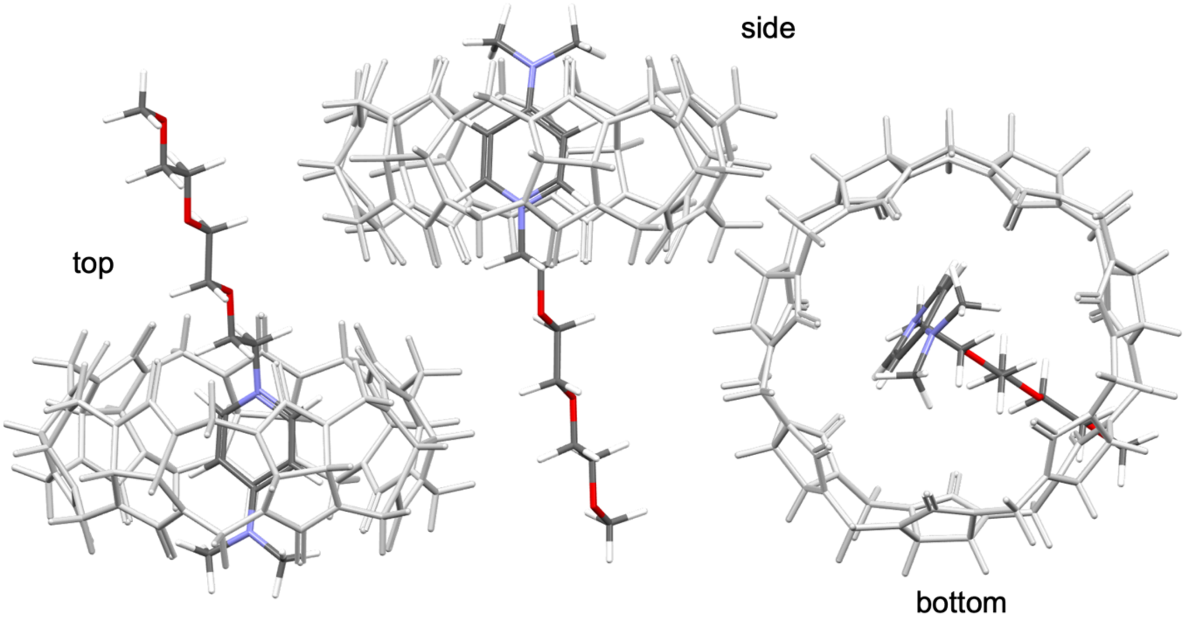

Model of **Ap3OMe** in CB7 cavity, showing slight protrusion form lower face. However, it is plausible that if the CB7 is bound to a surface this may be prevented, and the pyridinium nitrogen sits higher in the cavity opening.

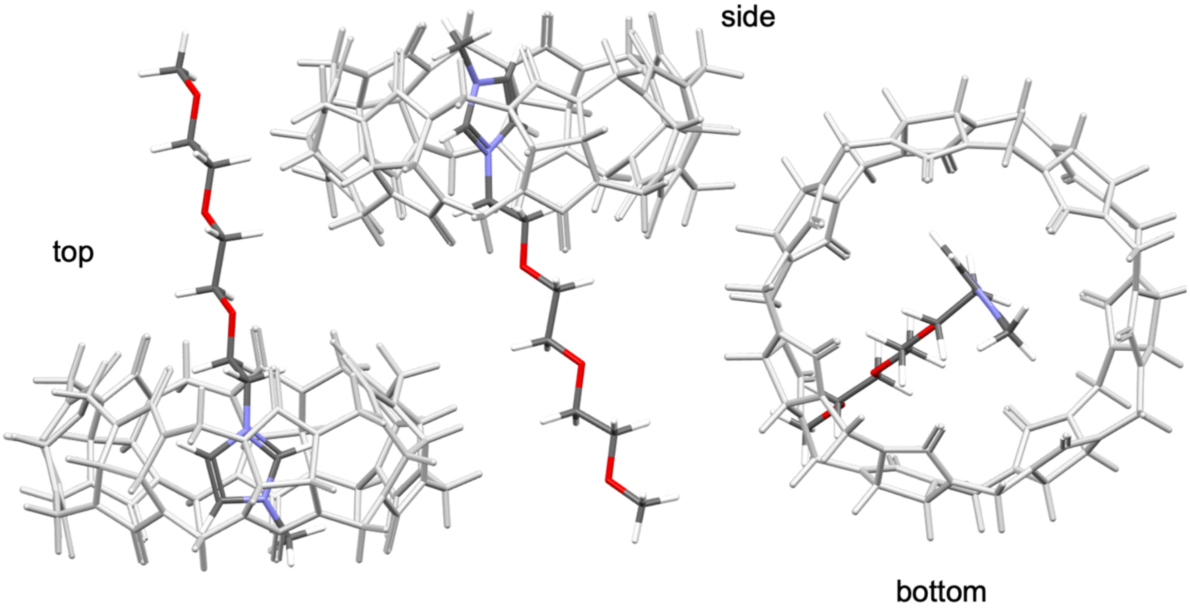

Model of **Im3OMe** in CB7 cavity showing good fit. It is plausible that if one portal is occluded then the imidazolium nitrogen may sit higher to interact more strongly with the open portal.

Figure S20 – NMR of Py3OMe•CB7, Ap3OMe•CB7 and Im3OMe•CB7

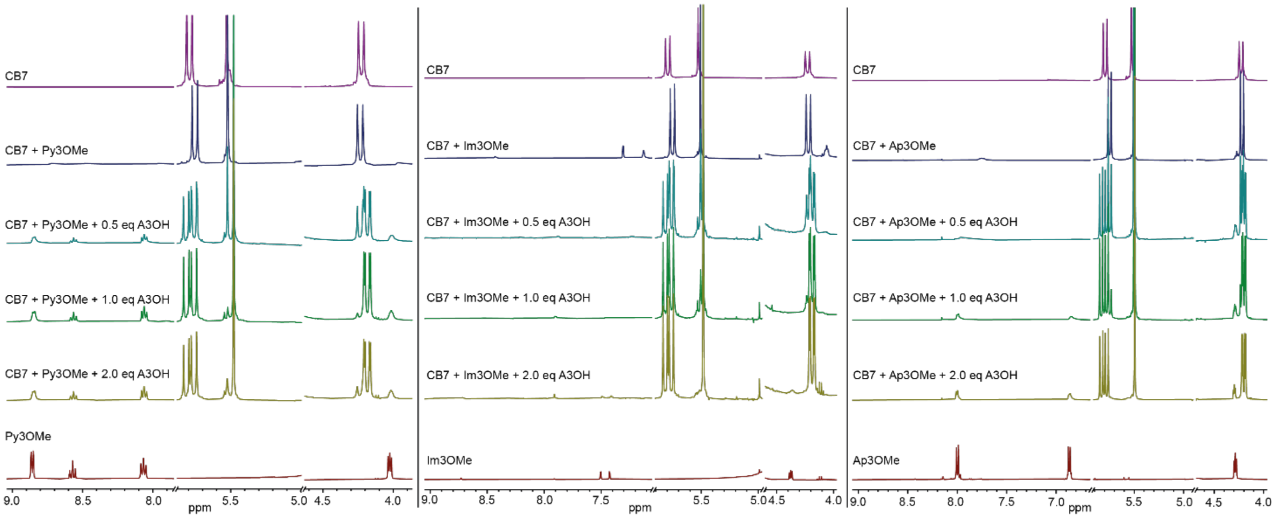

^{1}H NMR (D_2O) of **Py3OMe**, **Im3OMe** and **Ap3OMe** in 1:1 complex with CB7 (CB and aromatic regions shown around the HOD peak at 4.8 ppm). The aromatic protons are broadened and lost on binding CB7 due to dynamic exchange and the H_{int} peaks shift upfield. On addition of 0.5 to 2 equivalents of **A3OH** – a stronger competing guest – there are marked changes in the spectra, including re-emergence of the weaker guest proton resonances, and further splitting and shifting of the CB7 peaks.

Figure S21 – Isothermal calorimetry for Py3OMe, Ap3OMe and Im3OMe with CB7

The binding constants of CB7 with **Im3OMe**, **Py3OMe** and **Ap3OMe** were directly measured by isothermal titration calorimetry. The data points were fitted with the one site model. The small endothermic effect observed at the last stage of the titrations was due to solvent mismatch (syringe: pure H_2O, cell: 0.5% D_2O in H_2O) and has been subtracted before fitting.

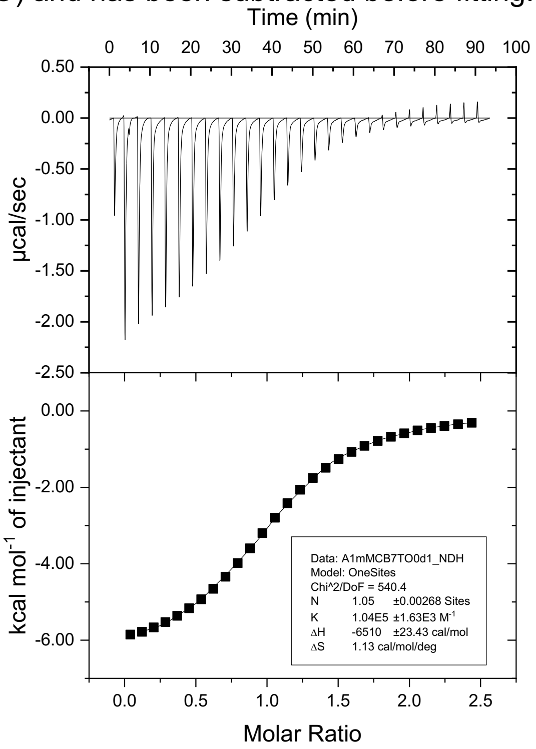

ITC isotherm for the titration of CB7 (1 mM) into **Im3OMe** (0.1 mM) in water at 25 °C. The binding constant of **Im3OMe**•CB7 was obtained to be (1.0 ± 0.1) × 10^5 M^{-1}.

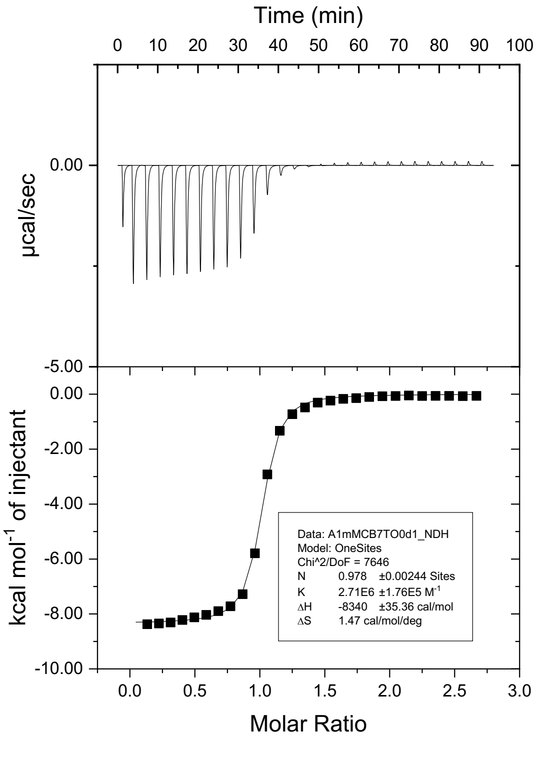

ITC isotherm for the titration of CB7 (1 mM) into **Ap3OMe** (0.1 mM) in water at 25 °C. The binding constant of **Ap3OMe•**CB7 was obtained to be (2.7 ± 0.2) × 10^6 M^{-1}.

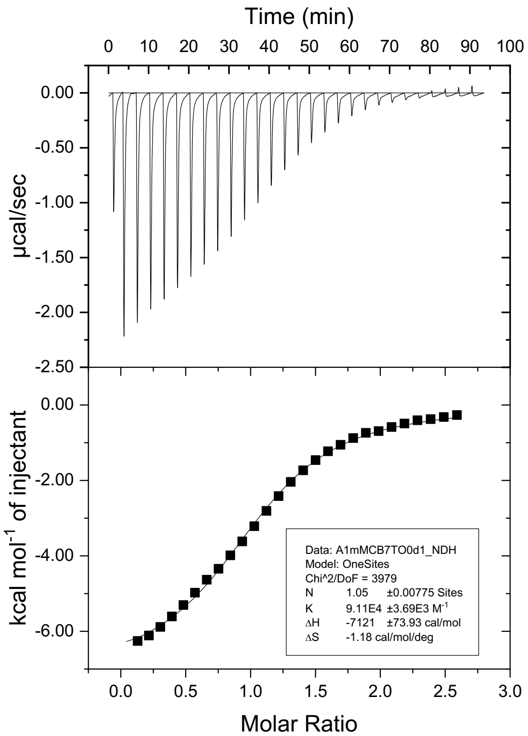

ITC isotherm for the titration of CB7 (1 mM) into **Py3OMe** (0.1 mM) in water at 25 °C. The binding constant of **Py3OMe•**CB7 was obtained to be (9.1 ± 0.4) × 10^4 M^{-1}.

Comparing the values found to similar literature compounds, **Im3OMe** (1.0 × 10^5 M^{-1}) can be thought of as similar to ionic liquid $C2MIm^+$ which has K_{ref} 1.8 × 10^5 M^{-1}.[9] **Py3OMe** (9.1 × 10^4 M^{-1}) can be roughly approximated by $C12Py^+$ (albeit with a long alkyl chain) with a K_{ref} 8.0 × 10^5 M^{-1}.[9] **Ap3OMe** (2.7 × 10^6 M^{-1}) can be approximated by $BAPH^{2+}$ in a 2:1 coordination to mimic the one portal binding, with K_{ref} 6.8 × 10^5 M^{-1}.[10]

Figure S21 – Additional DLS measures of Py16OMe, Ap16OMe and Im16OMe disrupting CdTe530/CB7 aggregates

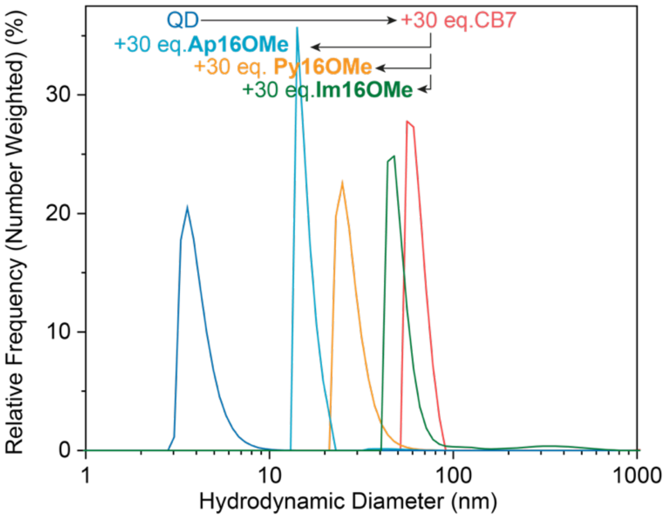

DLS of CdTe530 CB7 aggregates being disaggregated with three longer guests (see also Figure 5) containing a PEG750 (c. 16 EG monomomer units) spacer between guest and tail. As with the shorter versions, **Py16OMe** gives the most consistent results (and has the best fit to the CB7 cavity as a single portal binder), whereas **Ap16OMe** seems to lead to smaller aggregates than expected, maybe due to some removal of CB7 from the surface by being too large to act as a single portal binder, and **Im16OMe** struggling to disaggregate the clusters in a 1:1 ratio with the CB7 added.

General Materials and Methods

Reagents were used as supplied from Sigma Aldrich, Alfa Aesar, Fisher Scientific, Tokyo Chemical Industry or Lumiprobe without further purification. Reactions preformed at room temperature (RT) are run at approximately 20 °C. Water used was deionised (> 15 MΩ) and non-aqueous solvents used were of analytical grade. Thin layer chromatography was performed on Merck aluminium backed silica gel 60 F254 plates. Visualisation was achieved with UV light, iodine on silica, or $KMnO_4$ in basic aqueous solution, depending on the compound. Transformations carried out under an inert atmosphere were performed under an atmosphere of nitrogen. Where biphasic extraction was performed, Biotage ISOLUTE® Phase Separators were used to quickly remove the organic phase and retain the aqueous phase for subsequent collection.

DFT Methods

DFT calculations (structures shown in Figure 5b, Figure S4 and Figure S19) were performed using Spartan'16 Parallel Suite, and force-field calculations were performed using Chem3D. Geometry optimization was performed first using MMFF94, followed by full optimization at wB97X-D/6-31G* (vacuum) level of theory. All quantum mechanical calculations were done using restricted (closed-shell) models. The binding energy of the discrete inclusion complex was obtained by subtracting the energies of CB7 and free guest from that of the complex optimized and calculated at the same level of theory.

Instrumentation and Measures

Absorbance and fluorescence measurements were made on a Tecan Spark plate reader with monochromated light from an Xe flash lamp. Excitation wavelengths were set to best capture data without interference and are given in the text (typically 375 nm for QD excitation or 440 nm where direct dye/fluorescent protein excitation was to be avoided), band pass was typically set to 7.5 or 10 nm. Emission was measured between the excitation wavelength + 15 nm and 800 nm.

Samples were measured in Corning ½ area, UV-transparent, flat-bottomed 96-well plates. Working volumes were 75 µL, unless stated otherwise and for this volume the transmission path length was estimated to be 0.86 cm. All liquid handling steps were completed using calibrated single channel or electronic multichannel pipettes with an error of < 3%. Where imaging or premixing of solutions was required, the solutions were transferred from or to 1.5 mL Eppendorf tubes.

Fluorescence binding titrations were made in 2.5 ml BRAND disposable fluorescence cuvettes. The fluorescence spectra were recorded on a Jasco FP-8500 fluorimeter or Horiba DuettaBio absorbance and fluorescence spectrometer.

The isothermal titration calorimetry experiments were carried out on a MicroCal VP-ITC.

^{1}H NMR and ^{13}C NMR were collected on Bunker AVI 400 or 500 MHz spectrometers in deuterated solvents as noted. Chemical shifts are recorded in ppm, relative to the residual protonated solvent. Coupling constants are recorded in Hz. Mass spectra data for each compound were recorded on Agilent 6546 UHRA TOF or Bruker microTOFq system using positive mode electrospray ionisation (ESI) unless stated otherwise. Molecular ions or other major ion peaks are reported as *m/z*. Spectra are visually presented for novel compounds, or referenced against literature for known compounds.

Dynamic light scattering (DLS) was collected in backscattering mode on an Anton Paar Litesizer 500 with a 658 nm laser source in 1 cm path length quartz cuvettes. Water was the solvent in each case, and to estimate volume and number weighted intensities, for CdTe QDs, a refractive index of 2.9819 and an extinction coefficient of 0.35 m^{-1} was used, while for CdSe@Zns QDs, a refractive index of 2.5929 and extinction coefficient of 0.278 m^{-1} was used.[11]

TEM images were collected on a JEOL 1200 EX TEM running at an accelerating voltage of 80 kV. Digital micrographs were captured as tifs using a Cantega 2k x 2k camera with Olympus ITEM Software. Particle analysis was carried out using by binarising images in ImageJ before particle counting by edge detecting and fitting ellipses to the dark particles. Ellipses arising from image speckle/noise or larger multiparticle aggregates were discarded from the final count.

Synthesis of Nanoparticles

CdTe QDs

CdTe QDs were prepared following protocol from Tran *et al.*[12] The QDs were synthesised from a NaHTe intermediate. Tellurium powder (26 mg, 0.2 mmol) was dissolved in 5 mL of N_2 purged water followed by addition of $NaBH_4$ (19 mg, 0.5 mmol), and stirred under inert atmosphere at 85 °C for 1 h until a purple/red colour developed, and no solid Te residue could be seen.

In parallel, $Cd(OAc)_2$ (46 mg, 0.2 mmol) and 3-mercaptopropionic acid (30 µL, 0.34 mmol) were dissolved in 40 mL N_2 purged water. This solution was then basified with 1M aq. NaOH until the solution was at pH 12, turning the cloudy solution clear. A portion of the NaHTe solution (0.5 mL in an oxygen free syringe) was added to the Cd solution under an inert atmosphere, and a colour change was immediately observed from colourless to orange. This orange solution was heated to 100 °C for 1 to 4 h, until the desired fluorescence wavelength was observed with a hand-held 365 nm lamp.

Typically, CdTe530 required a growth time of 60 minutes, and CdTe540 a growth time of 240 minutes. Concentrations were estimated as detailed above (**Table S1**).

CdSe@ZnS QDs

CdSe@ZnS synthesis adapted from protocol by Shen *et al.*[13] In a flask, Se (0.19 g, 2.4 mmol) and octadecylamine (1.94 g, 7.2 mmol) were dissolved in 18 mL octadecene (ODE) and heated to 100 °C under Schlenk conditions. The reaction was stirred at 100 °C for 20 mins. Subsequently, the reaction was heated to 220 °C for 3 h. During this process the colour of the mixture changed from colourless to red and finally turned yellow.

Separately, CdO (15.4 mg, 0.12 mmol) and oleic acid (102 mg, 0.36 mmol) were dissolved in 5 mL ODE and heated to 280 °C under Schlenk conditions until the solution turned clear and colourless. Then, 2 mL of the Se solution was injected into the flask, resulting in a rapid colour change from orange to deep red with increasing reaction time. After 1 min of growth post Se solution injection, the sample was removed from the heat, producing green CdSe QDs. The QDs were precipitated in EtOH and centrifuged at 4000 rpm for 15 mins and resuspended in 5 mL hexane.

To create the CdSe@ZnS core shell particles, a method adapted from Peveler et al. was used.[14] In a flask under Schlenk conditions, zinc diethyldithiocarbamate (0.26 g, 0.7 mmol), oleylamine (3 mL, 9.12 mmol), trioctylphosphine (3 mL, 6.73 mmol) were dissolved in 10 mL ODE. To this, 2 mL CdSe QDs in hexane, was added by injection and the resultant mixture was heated slowly (around 3 °C/min) under partial vacuum at 70 °C to remove the hexane. The solution was then heated to 120 °C under an inert atmosphere. After 2 h, the reaction was cooled, and the QDs were precipitated with EtOH and isolated with centrifugation at 4000 rpm before being resuspended in 3 mL hexane for storage.

To phase exchange the samples to be water soluble a biphasic ligand exchange with glutathione was performed as previously reported.[15,16] To transfer the QDs into water, 1.4 nmol of QDs were suspended in 100 µL chloroform. In a separate Eppendorf tube, glutathione (40 mg, 0.13 mmol) and tetramethylammonium hydroxide (100 µL, 25% in methanol) were dissolved in 200 µL borate buffer (50

mM, pH 9). The chloroform solution was added to the glutathione solution, and the sample was vortexed before heating at 55 °C for 20 h. The colour of the QD solution moved into the aqueous layer, which was extracted and the QDs precipitated with a 1:1 mixture of MeOH: acetone, before centrifugation and resuspension of the QDs in borate buffer. The sample was stored in the dark at 4 °C.

Production of RFP

10xhis-RFP-Halotag plasmid was transformed into *E. coli* BL21(DE3) start pLys cells. Transformed cells were plated on a Luria-Bertani (LB) agar plate containing 50 µg/mL of Kanamycin and grown at 37 °C for 16 h. A single colony was used to inoculate 100 mL of LB broth, supplemented by 50 µg/mL Kanamycin and the culture incubated at 37 °C, 200 rpm for 16 h.

This overnight culture was used at a dilution of 1:100 to inoculate fresh LB supplemented with the same antibiotic, which was incubated at 37 °C, 200 rpm until the optical density at 600 nm reached 0.6. The culture was cooled to 4 °C before the addition of 100 µM of isopropyl β-D-1-thiogalactopyranoside to induce protein expression, which was carried out at 22 °C, 200 rpm for 16h.

Cells were harvested by centrifugation for 15 min at 5,000 × g, resuspended in lysis buffer (150 mM NaCl, 20 mM Bis-Tris pH 6.8, 20 mM imidazole and 1.5 mM β-mercaptoethanol) and lysed in a cell disruptor at 30,000 psi. Cell debris was removed by centrifugation at 50,000 × g for 20 min at 4 °C and the supernatant was further cleared by filtration using a 0.22 µm nitrocellulose filter, before being applied to a 5 mL FF Histrap column pre-equilibrated in lysis buffer. The column was extensively washed using a step-gradient of increasing imidazole in lysis buffer (50 mM and 100 mM) and the UV absorbance at 280 nm allowed to return to baseline each time.

Protein was then eluted with lysis buffer supplemented with 300 mM imidazole. Elution fractions were pooled and concentrated using a 30 kDa cut-off concentrator, before injection onto a Superdex 200 16/600 size exclusion column, equilibrated with 150 mM NaCl, 20 mM Tris pH 8 and 5% glycerol. The peak fractions were pooled, and purity of the protein analysed by SDS-PAGE. The protein was concentrated further using a 30 kDa cut-off filter to a concentration of 420 µM, aliquoted, flash frozen in liquid N_2 and stored at -80 °C for further use.

Synthesis and Characterisation of Molecular Guests

Cucurbit[7]uril (CB)

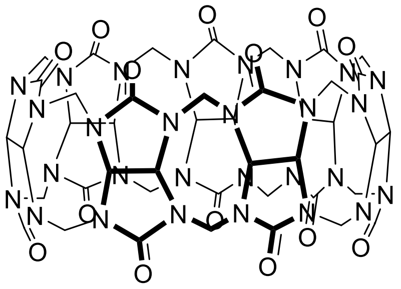

Synthesis adapted from Day *et al.* and Kim *et al.*[17,18] Glycouril (99.3 g, 0.70 mol) and paraformaldehyde (44.25 g, 1.47 mol) were mixed in a round bottom flask and stirred vigerously with a glass rod. The flask was cooled to 0 °C and 141 mL conc. HCl was added portion-wise with continued stirring. Stirring was stopped once the mixture had solidified completely. The reaction was heated over 2 h to 80 °C and maintained at 80 °C for 2.5 h, yielding a red solution. This mixture was then heated to 100 °C for a further 18 h. The solution was cooled to RT and the residual solid (CB6 and CB8) was filtered off. The filtrate was reduced to 75 mL by heating, and added to a mixture of 375 mL MeOH and 25 mL water, with magnetic stirring, to precipitate CB5 and CB7.

To obtain pure CB7, the precipitate containing a mixture CB5/CB7 was dissolved in 1L water, this was then filtered to remove any residual solid CB6. The now clear solution was stirred in a large conical flask at RT and MeOH was added gradually until the mixture turned cloudy. At this point addition of MeOH was stopped but stirring was continued for a further 1h. After this time stirring was halted to allow for sedimentation of the precipitated CB7, which was collected by filtration and dried in a vacuum oven. The process was repeated multiple times and in total, ca. 3 g of pure CB7 was isolated.

^{1}H NMR (600 MHz D_2O/DCl) δH 5.73 (14H, d, J=15.3, H_{int}), 5.47 (14H, s, H_{eq}), 4.17 (14H, d, J=15.3, H_{ext}).

13C NMR (150 MHz D_2O) δC 157.1, 69.8, 50.7.

MS (ESI+ in presence of di-cationic bis-imidazolium guest) m/z 733.53 (expected $[M+BisIm]^{2+}$ $C_{60}H_{74}N_{32}O_{14}$ calc. 733.30).

Fresh stock solutions at 5 mM (5.8 mg/mL) were prepared in water for experiments and diluted as necessary.

Adamantyl triethylene glycol (A3OH)

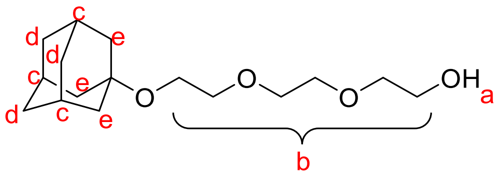

Synthesis and characterisation of this molecule is given in the main text Methods and corresponds with literature.[19,20]

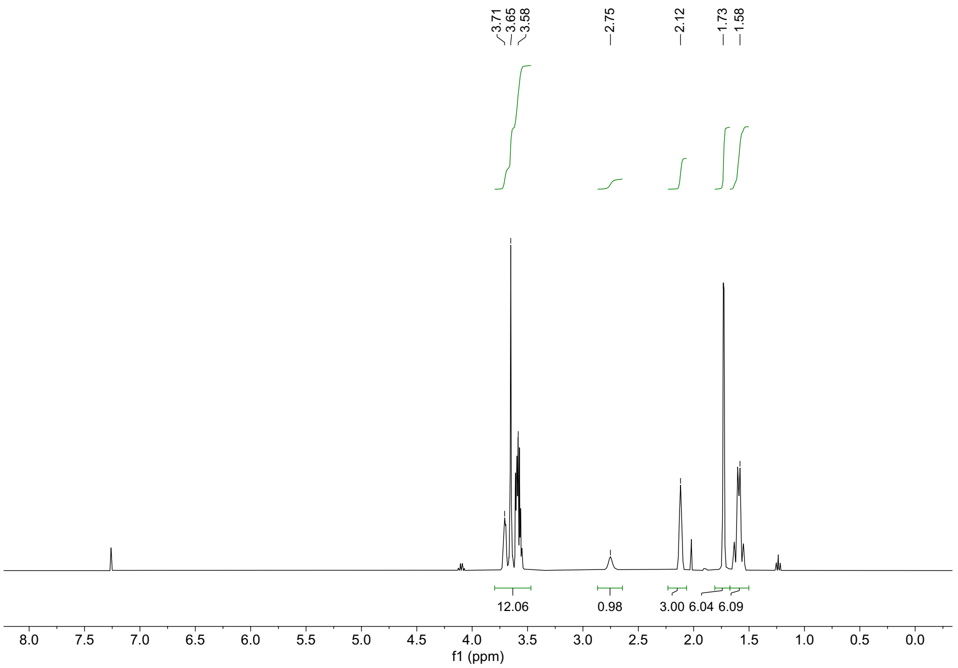
3.71
3.65
3.58
2.75
2.12
1.73
1.58
12.06
0.98
3.00
6.04
6.09
8.0
7.5
7.0
6.5
6.0
5.5
5.0
4.5
4.0
3.5
3.0
2.5
2.0
1.5
1.0
0.5
0.0
f1 (ppm)

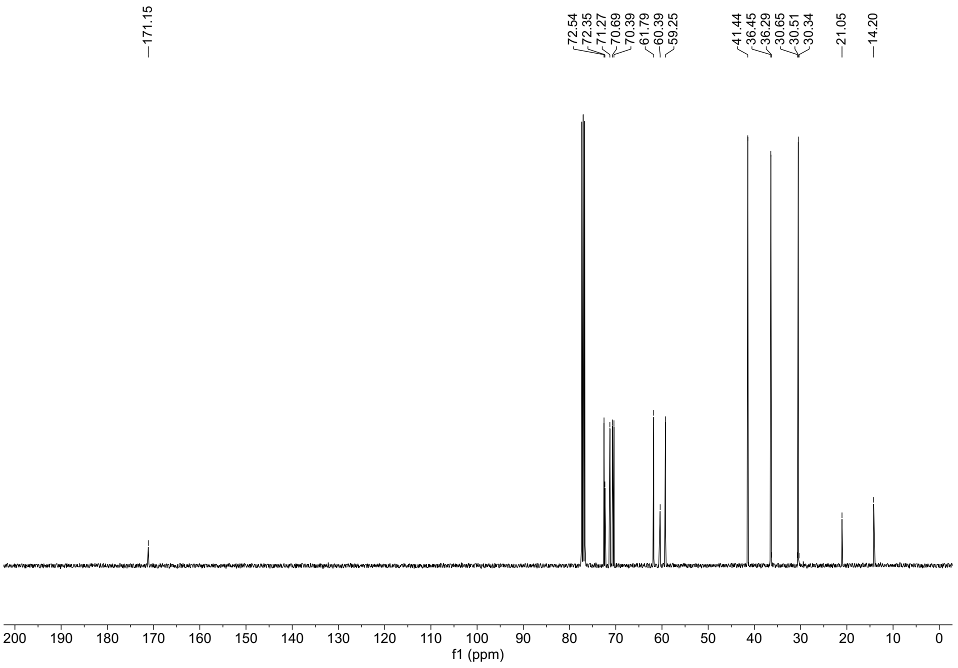
171.15
72.54
72.35
71.27
70.69
70.39
61.79
60.39
59.25
41.44
36.45
36.29
30.65
30.51
30.34
21.05
14.20
200
190
180
170
160
150
140
130
120
110
100
90
80
70
60
50
40
30
20
10
0
f1 (ppm)

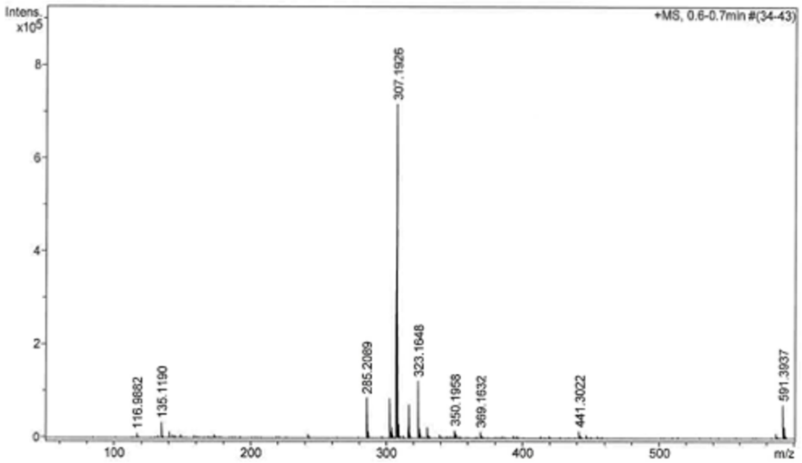

Adamantyl polyethylene glycol 400 (A8OH*)*

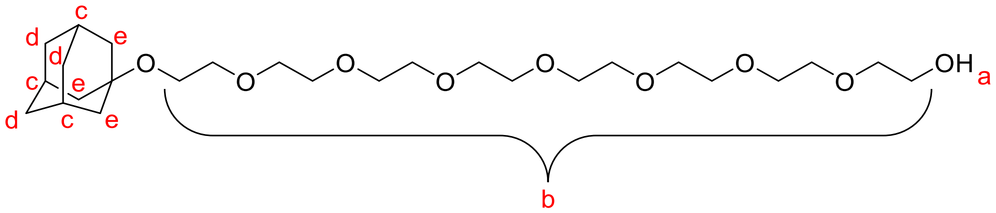

Synthesis based on that of **A3OH**. Polyethylene glycol MW400 (*c.* 8-9 ethylene glycol units 13.3 g, 33.2 mmol), 1-bromoadamantane (1.4g, 6.4 mmol), Et_3N (3 mL, 21.5 mmol) and 1,8-diazabicycloundec-7-ene (49 µL, 0.33 mmol) were stirred together and heated at 110 ˚C for 18 h. The reaction was diluted with 1 M aq. HCl (27.5 mL) and extracted into DCM (2 × 25 mL). The organic layer was washed with water (2 × 25 mL) and dried with $MgSO_4$ to yield a crude brown oil. This oil was further washed with (4 × 50 mL) 2M HCl and (1 × 50 mL) brine, yielding the product as an orange oil (270 mg, 8%). The low yield was ascribed to losses during washing to obtain a pure product.

^{1}H NMR (400 MHz, $CDCl_3$) δ 3.78 – 3.52 (m, ~32H, H_b), 2.64 (s, 1H, H_a), 2.20 – 2.10 (m, 3H, H_c), 1.74 (d, J = 2.9 Hz, 6H, H_d), 1.70 – 1.50 (m, 6H, H_e).

^{13}C NMR (101 MHz, $CDCl_3$) δ 77.26, 72.60, 72.52, 72.45, 72.33, 72.28, 71.25, 71.23, 71.21, 70.58, 70.53, 70.52, 70.49, 70.47, 70.43, 70.31, 70.27, 70.20, 70.14, 61.72, 61.65, 61.57, 61.53, 59.25, 59.16, 41.48, 41.46, 41.42, 36.45, 30.50.

MS (ESI+) *m/z* 527.3153 ([A8OH+Na]$^+$ $C_{26}H_{48}O_9Na^+$ calc. 527.3191), 571.3420 (([A9OH+Na]$^+$ $C_{28}H_{52}O_{10}Na^+$ calc. 527.3453), A7OH – A13OH observed in mixture.

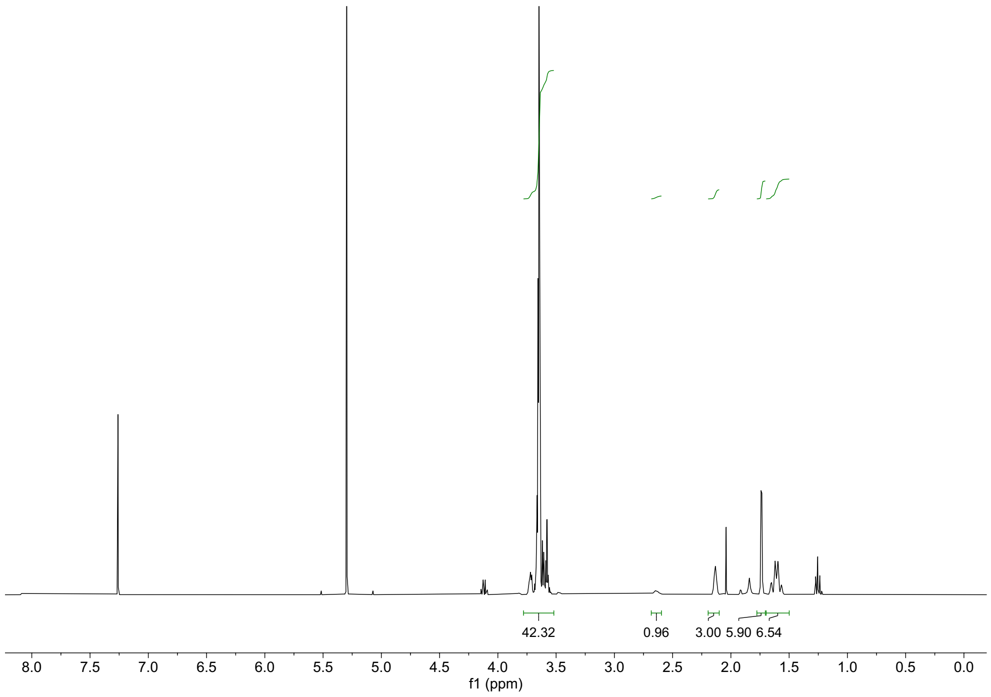

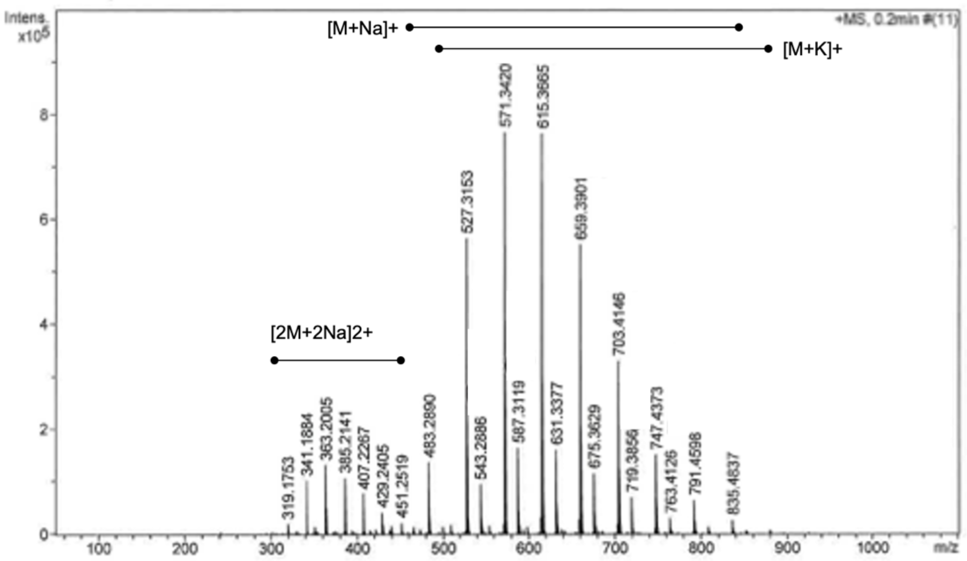

Adamantyl triethylene glycol azide (A3N$_3$)

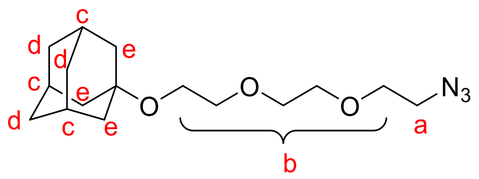

Synthesis adapted from Gustafson *et al*.[19] **A3OH** (400 mg, 1.4 mmol) with MsCl (140 µL, 2.1 mmol) and Et$_3$N (506 µL, 4.2 mmol) in 10 mL DCM at RT for 20 h. This reaction mixture was subsequently diluted with 10 mL 1 M aq. HCl and extracted into DCM (2 × 10 mL), the organic layers were combined and

washed with water (2 × 20 mL). The intermediate was dried over a hydrophobic frit and the solvent was removed *in vacuo*. This species was used without further purification (507 mg, 1.39 mmol).

The mesylate intermediate was dissolved in 5 mL of DMF. NaN_3 (218 mg, 3.36 mmol) was added to the reaction mixture and the reaction was heated to 80 °C for 20 h. The reaction mixture was subsequently diluted with 7 mL of water and extracted into ethyl acetate (2 × 7 mL). Organic layers were combined and washed with water (2 × 14 mL). The resultant crude product was dried over a hydrophobic frit and solvent removed *in vacuo*. The product was purified by column chromatography (10% MeOH in chloroform) and the appropriate factions combined and dried to give the product as a brown oil (255 mg, 52.4 %).

^{1}H NMR (400 MHz, $CDCl_3$) δ 3.75 – 3.54 (m, 10H, H_b), 3.42 (d, J = 4.6 Hz, 2H, H_a), 2.19 – 2.13 (m, 3H, H_c), 1.77 (m, J = 2.9 Hz, 6H, H_d), 1.70 – 1.59 (m, 6H, H_e).

^{13}C NMR (101 MHz, $CDCl_3$) δ 72.24, 71.39, 70.75, 70.70, 70.06, 59.30, 50.73, 41.51, 36.48, 30.52.

MS (ESI+) m/z 332.1935 (expected $[M+Na]^+$ $C_{16}H_{27}N_3O_3Na^+$ 332.1945)

***NB extreme care** and appropriate procedures should be used when handling NaN_3 and other small molecule azides, due to the risk of generating explosive metal azide salts or organic azides, or toxic HN_3 gas!*

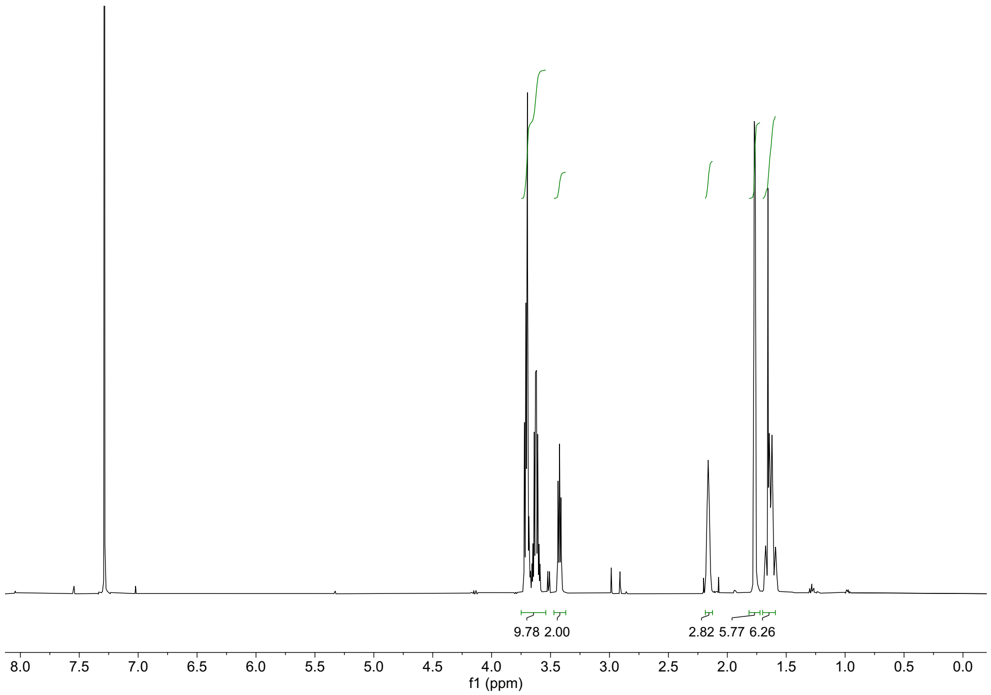

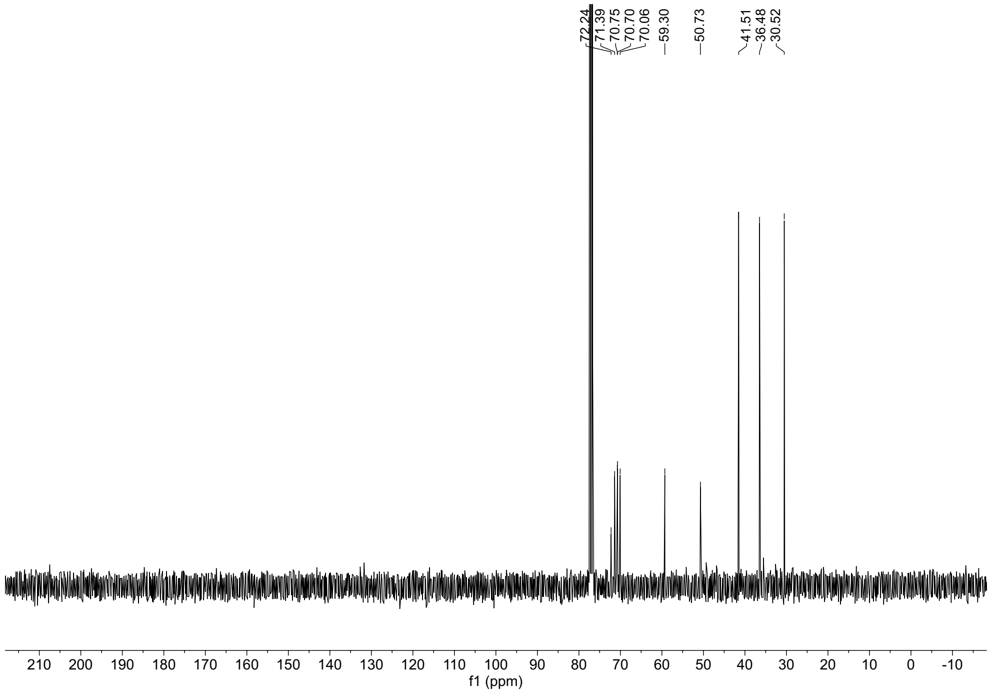

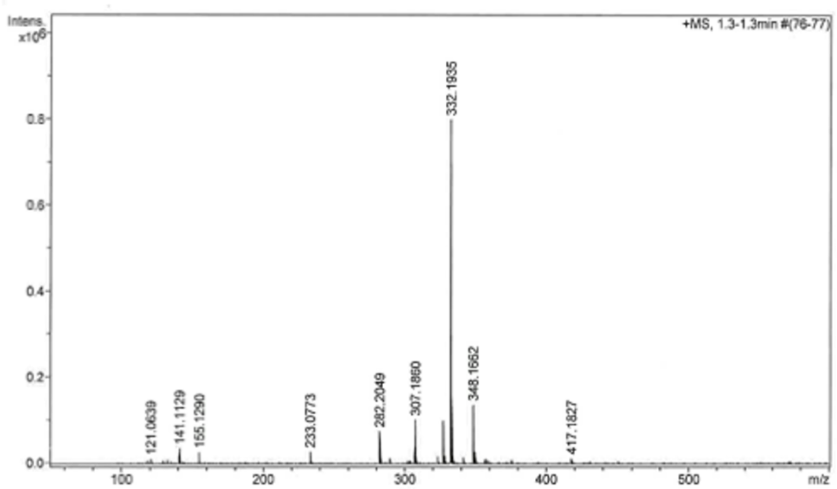

Adamantyl triethylene glycol amine (A3NH_2)

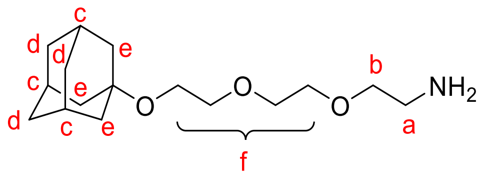

Synthesis adapted from Gustafson *et al.*[19] **A3N_3** (250 mg, 0.81 mmol) and triphenyl phosphine (275 mg, 1.05 mmol) were dissolved in 5 mL THF and stirred at RT for 2 h. After 2 h, water (29 µL) was added, and the reaction was left to stir at RT for 20 h. The mixture was diluted with 5 mL 1 M aq. HCl, and the mixture was washed with (2 × 10 mL) ethyl acetate. The aqueous layer was basified with 20 mL 3M aq. NaOH and the resulting solution was extracted into (4 × 25 mL) DCM. The solution was dried over a hydrophobic frit and solvent removed *in vacuo* producing A3NH_2 as a brown oil (150 mg, 65.6 %).

^{1}H NMR (400 MHz, $CDCl_3$) δ 3.71 – 3.54 (m, 8H, H_f), 3.51 (t, J = 5.0 Hz, 2H, H_b), 2.86 (t, J = 5.3 Hz, 2H, H_a), 2.17 – 2.07 (m, 3H, H_c), 1.79 – 1.71 (m, 6H, H_d), 1.66 – 1.53 (m, 6H, H_e).

^{13}C NMR (101 MHz, $CDCl_3$) δ 72.80, 71.30, 70.58, 70.31, 59.27, 41.63, 41.50, 36.46, 30.52.

MS (ESI+) m/z 284.2157 (expected $[M+H]^+$ $C_{16}H_{30}NO_3^+$ 284.2220)

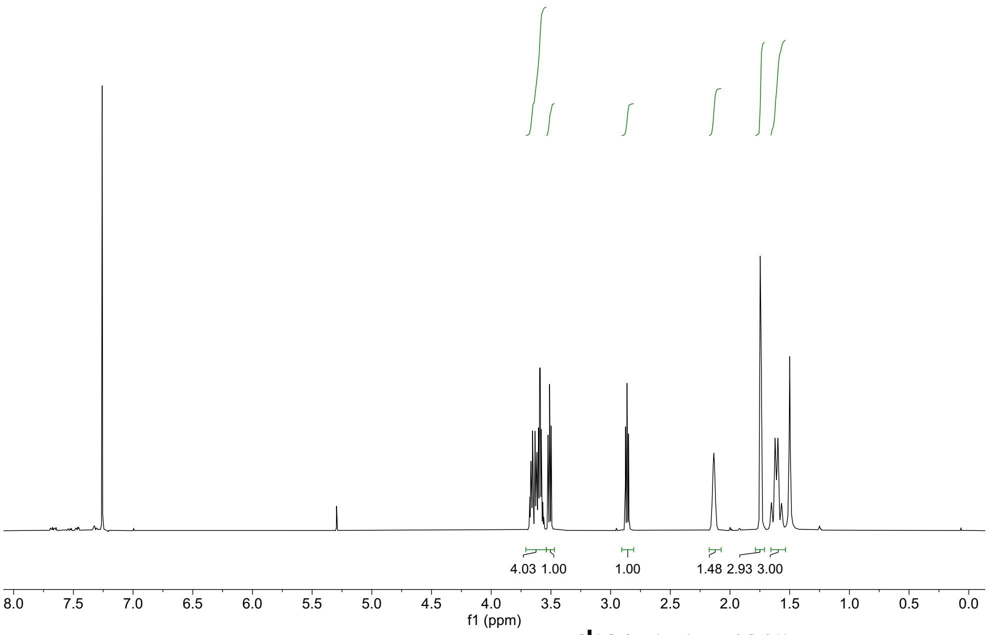

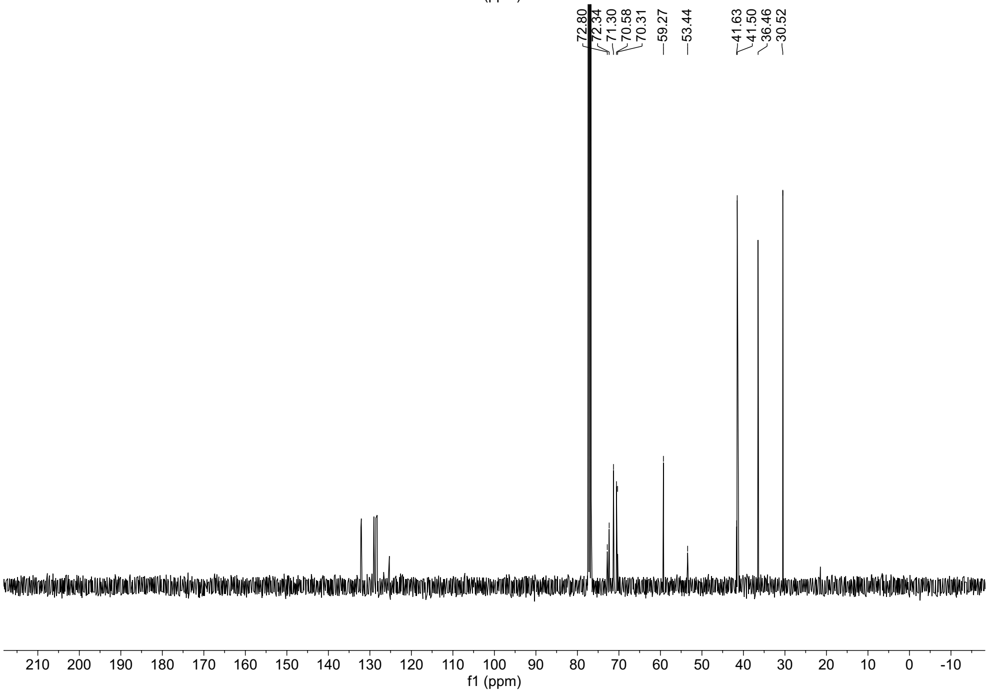

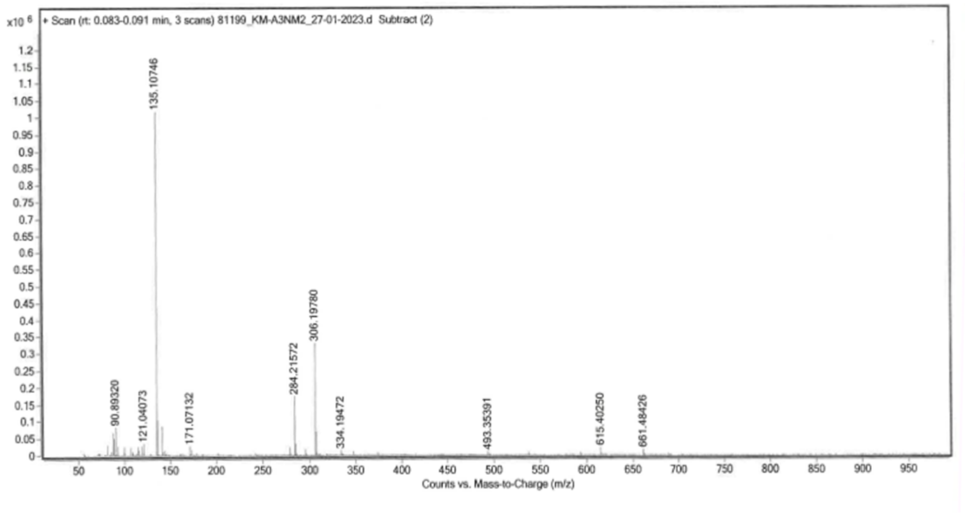

Adamantyl triethylene glycol sulfoCy3.5 amide (A3Cy3.5)

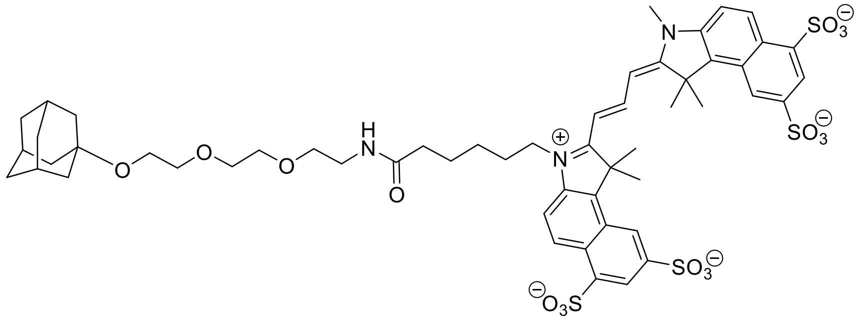

A3NH₂ (800 µL, 1.38 µmol, from a 2.2 mg/mL stock solution in DMF) was added dropwise with a syringe into a solution of sulfo-cy3.5 NHS ester (1.5 mg, 1.38 µmol, Lumiprobe) in 1 mL DMF. The reaction was stirred at RT for 20 h. Subsequently, the solvent was removed *in vacuo* to yield the product as a purple powder (1.60 mg, 92.5%). Due to the small amount of material made, NMR characterisation was not possible, but mass spectrometry showed a single product with expected mass characteristics.

MS (ESI+) m/z 379.7583 (expected $[M]^{3-}$ $C_{54}H_{64}N_3O_{16}S_4^{3-}$ calc. 379.4395)

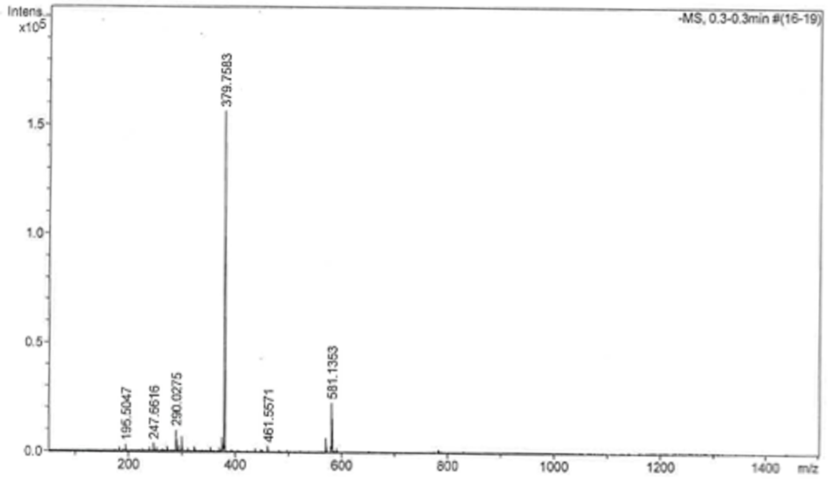

Adamantyl triethylene glycol biotin amide (A3biotin)

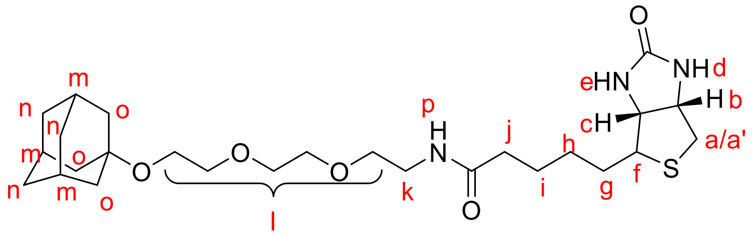

A3NH$_2$ (100 mg, 0.35 mmol), biotin-NHS ester (120 mg, 0.35 mmol, Tokyo Chemical Industry) and Et$_3$N (147 µL, 1.05 mmol) were dissolved in 2 mL DMF at heated at 80 °C for 20 h. The sample was concentrated *in vacuo* and purified by column chromatography (10% MeOH in ethyl acetate), yielding the product as a white powder (128 mg, 72.7%).

^{1}H NMR (400 MHz, CDCl$_3$) δ 6.32 (s, 1H, H_p), 5.20 (s, 1H,H_e), 4.54 (m, 2H, H_b, H_d), 4.35 (m, 1H, H_c), 3.74 – 3.53 (m, 10H,H_l), 3.52 – 3.35 (m, 2H, H_k), 3.20 (t, J = 4.9 Hz, 1H, H_f), 2.96 (dd, J = 12.7, 4.8 Hz, 1H, H_a), 2.75 (dd, J = 13.0, 4.8 Hz, 1H, $H_{a'}$), 2.28 – 2.22 (m, 2H, H_j), 2.17 (m, 3H, H_m), 1.86 – 1.43 (m, 18H, H_g, H_h, H_i, H_n, H_o).

^{13}C NMR (101 MHz, CDCl$_3$) δ 173.37, 164.16, 72.37, 71.24, 70.38, 70.18, 70.00, 61.83, 60.25, 59.25, 55.69, 41.52, 40.56, 39.21, 36.45, 36.04, 30.50, 28.28, 28.14, 25.64.

MS (ESI+) *m/z* 510.3007 (expected $[M+H]^+$ $C_{26}H_{44}N_3O_5S^+$ calc. 510.2996).

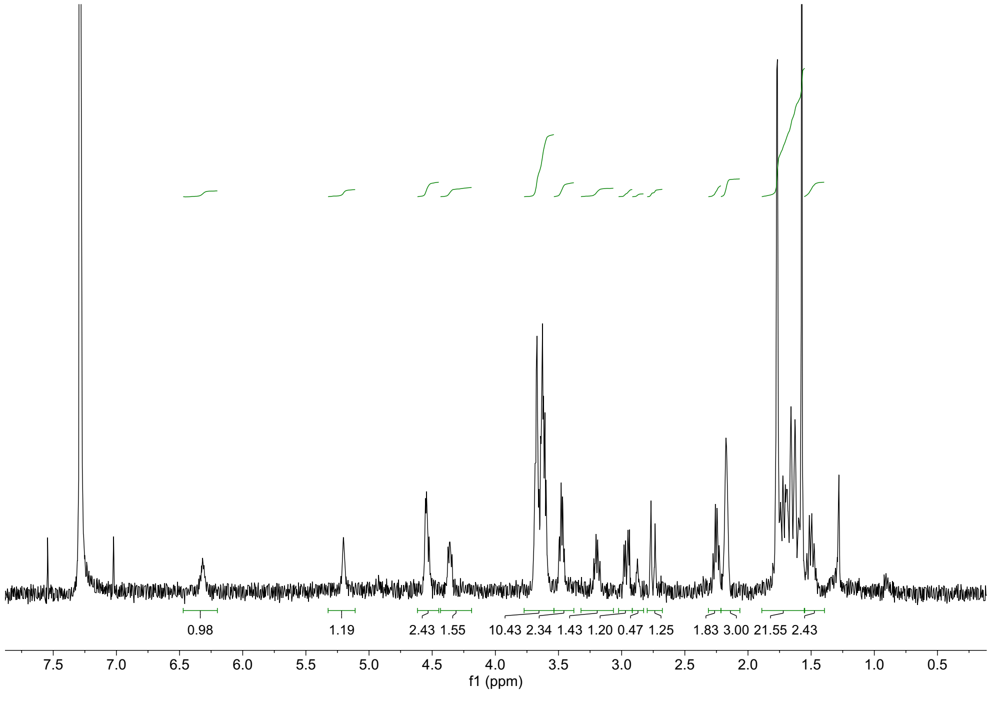

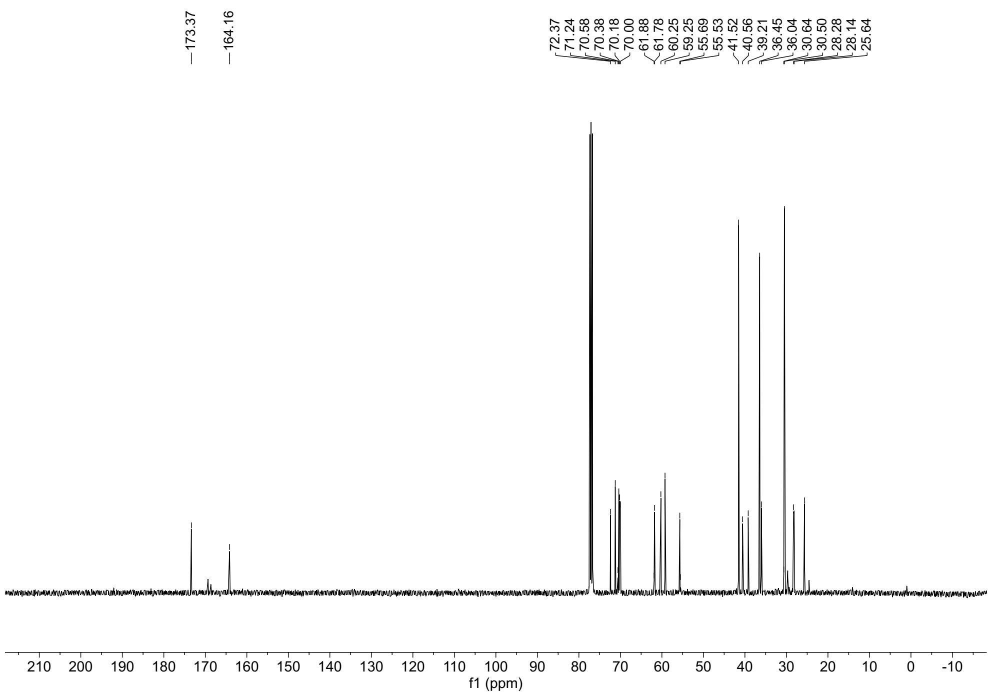

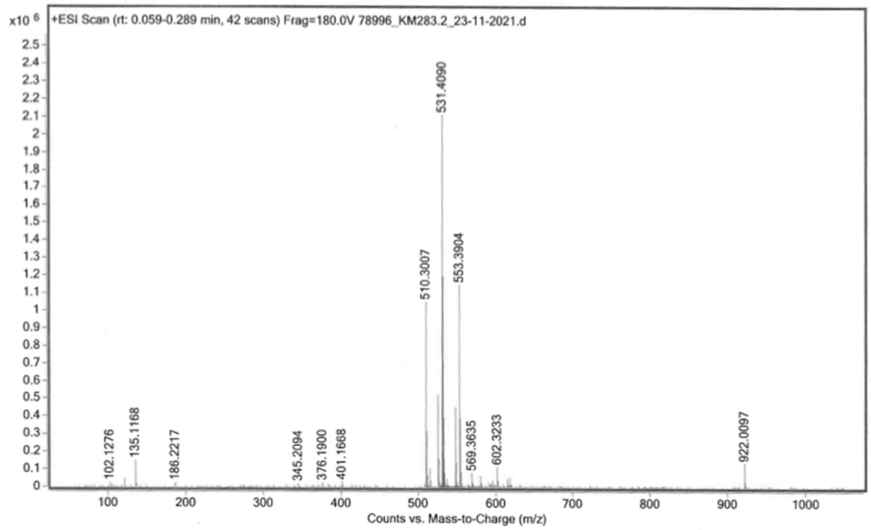

Adamantyl triethylene glycol-*O*-1-chlorohexane (A3Halo)

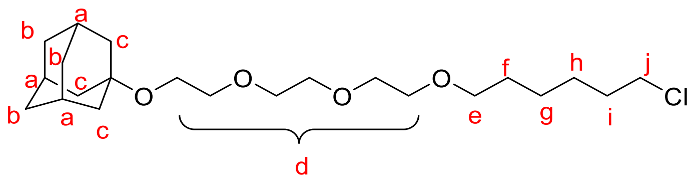

A3OH (175 mg, 0.61 mmol) in 1 mL anhydrous THF was added dropwise to NaH (48.8 mg, 1.22 mmol, 60% dispersion in mineral oil) suspended in 1.44 mL anhydrous THF, at 0 °C under an inert atmosphere. The resultant solution was stirred at 0 °C for 30 mins. To the reaction mixture, 1-chloro-6-iodohexane (0.13 mL, 0.85 mmol) was added dropwise before the reaction mixture was warmed to RT and stirred for 16.5 h. The reaction mixture was cooled to 0 °C and quenched by the dropwise addition of 4 mL saturated aq. NH_4Cl before being extracted with ethyl acetate (3 × 10 mL). The combined organic layers were washed with water (10 mL) and brine (2 × 10 mL), dried over $MgSO_4$, and concentrated *in vacuo* to give the crude product as a yellow oil. The crude residue was purified by column chromatography (20% ethyl acetate in petroleum ether) to give the desired product as a colourless oil (112 mg, 41% yield).

^{1}H NMR (400 MHz, CDCl3) δ 3.76 – 3.39 (m, 16H, H_d, H_e, H_j), 2.16 – 2.11 (m, 3H, H_a), 1.85 – 1.68 (m, 8H, H_b, H_e), 1.66 – 1.52 (m, 8H, H_c, H_i), 1.53 – 1.12 (m, 4H, H_g, H_h).

^{13}C NMR (101 MHz, CDCl3) δ 72.23, 71.30, 71.25, 70.65, 70.15, 59.28, 45.07, 41.50, 36.49, 32.57, 30.53, 29.49, 26.73, 25.45.

MS (ESI+) *m/z* 425.2442 (expected $[M+Na]^+$ $C_{22}H_{39}ClO_4Na^+$ calc. 425.2429).

The limiting aqueous solubility of this compound prevented its further exploitation.

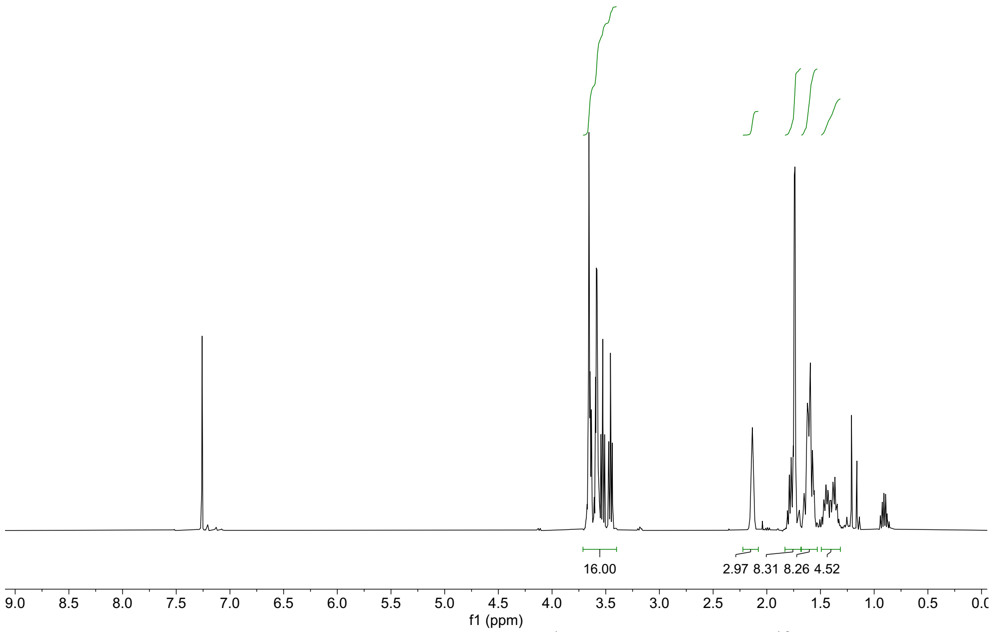

Some residual mineral oil is apparent below 1.25 ppm (^{1}H) and 25 ppm (^{13}C)

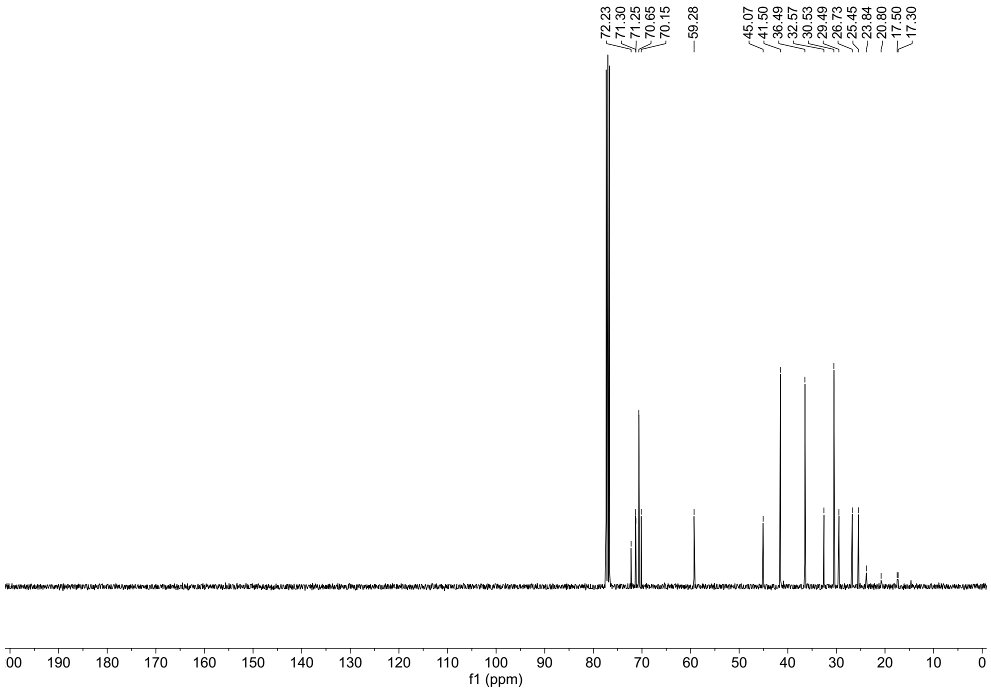

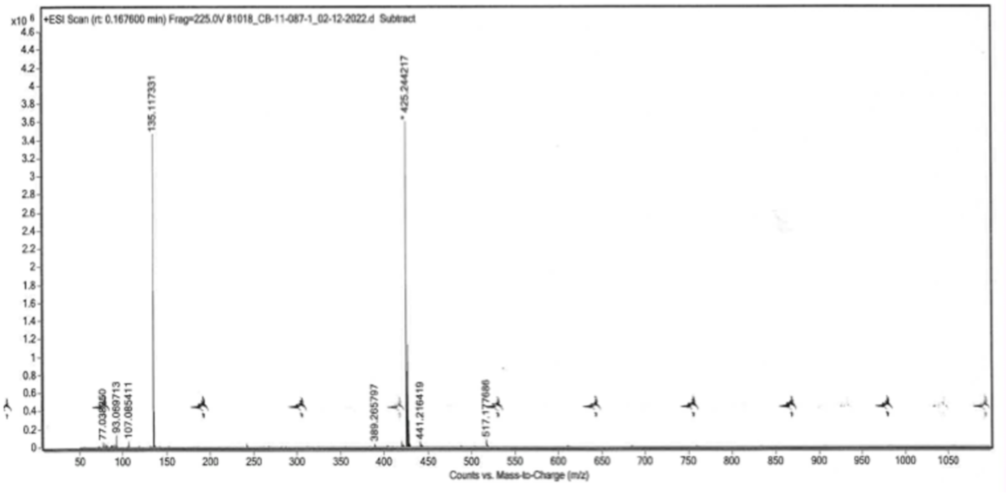

tert-butylcarbonyl-tetraethylene glycol (BocNH4OH)

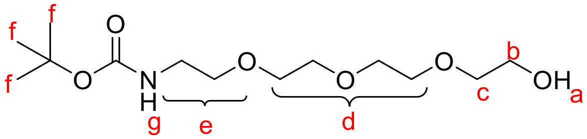

Synthesised from the method of Zhang *et al.*[21] To a solution of Boc_2O (2.03 g, 9.3 mmol) and tetra-ethylene glycol amine (1.50 g, 7.8 mmol) in 30 mL DCM. Et_3N (1.2 mL, 8.5 mmol) was added dropwise. The reaction mixture was concentrated *in vacuo* to give the crude product as a pale-yellow solid. The crude product was purified by column chromatography (gradient of 50-100% ethyl acetate in petroleum ether), the desired fractions were combined and solvent was removed to give the desired product as a pale-yellow oil (1.63 g, 72% yield). Observed analytical data matched that of the original work.

1H NMR (400 MHz, $CDCl_3$) δ 5.63 (s, 1H, H_g), 3.76 – 3.68 (m, 4H, H_e), 3.67 – 3.59 (m, 8H, H_d), 3.55 – 3.50 (m, 2H, H_c), 3.35 – 3.27 (m, 2H, H_b), 3.01 (s, 1H, H_a), 1.44 (s, 9H, H_f).

MS (ESI+) m/z 316.20 (expected $[M+Na]^+$ $C13H27NO6Na^+$ calc. 316.20).

tert-butylcarbonyl-tetraethylene glycol-*O*-1-chlorohexane (BocNH4Halo)

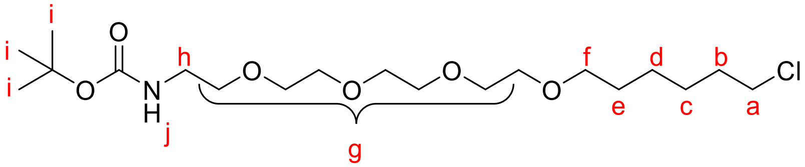

Synthesised from the method of Steinebach *et al.*[22] To a suspension of NaH (123 mg, 3.06 mmol, 60% dispersion in mineral oil) in 3mL anhydrous THF at 0 °C, **BocNH4OH** (750 mg, 2.55 mmol) was added. The resulting solution was stirred at 0 °C for 30 min. 1-Chloro-6-iodohexane (0.54 mL, 3.57 mmol) was added dropwise and the reaction mixture stirred at RT for 21 h. The reaction mixture was quenched by addition of sat. aq. ammonium chloride solution before being extracted with DCM (3 × 10 mL). The combined organic layers were washed with water (2 × 10 mL) and brine (10 mL), dried over $MgSO_4$, and concentrated *in vacuo* to give the crude product as a yellow oil. The crude product was purified by column chromatography (gradient of 30–100% ethyl acetate in petroleum ether) to give the desired product as a pale-yellow oil (461 mg, 44% yield). Observed analytical data matched that of the original work.

^1H NMR (400 MHz, $CDCl_3$) δ 5.02 (s, 1H, H_j), 3.69 – 3.43 (m, 18H, H_a, H_b, H_g), 3.35 – 3.26 (m, 2H, H_h), 1.83 – 1.72 (m, 2H, H_b), 1.64 – 1.55 (m, 2H, H_c), 1.50 – 1.31 (m, 13H, H_d, H_e, H_i).

MS (ESI+) m/z 434.20 (expected $[M+Na]^+$ $C_{19}H_{38}ClNO_6Na^+$ calc. 434.20)

Adamantyl acetamide tetraethylene glycol-*O*-1-chlorohexane (A4Halo)

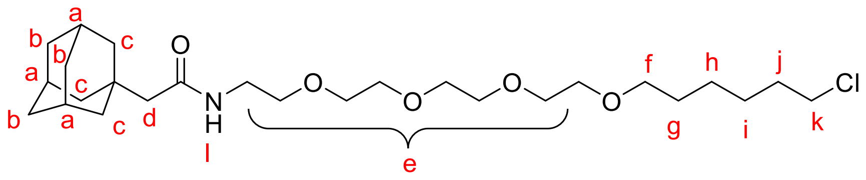

BocNH4Halo (34 mg, 0.081 mmol) was dissolved in a mixture of 0.5 mL trifluoroacetic acid and 0.5 mL DCM, the reaction was stirred at RT for 3.5 h. The reaction was monitored by TLC for complete consumption of the starting material before removal of volatile components *in vacuo*. The residue was dissolved 0.4 mL anhydrous DMF and DIPEA (85 µL, 0.48 mmol) was added. Separately, adamantane acetic acid (16 mg, 0.080 mmol) and HATU (30 mg, 0.080 mmol) were dissolved in 0.4 mL anhydrous DMF. The two solutions were combined and stirred at RT for 22 h. The reaction mixture was quenched by the dropwise addition of 0.8 mL 1M aq. HCl and stirred at RT for 10 min. The reaction mixture was extracted with DCM (3 × 10 mL). The combined organic layers were washed with 5% aq. lithium chloride (6 × 3 mL), dried over $MgSO_4$, and concentrated *in vacuo* to give the crude product as an orange solid. The crude product was purified by column chromatography (gradient of 50–95% diethyl ether in petroleum ether, followed by 50–100% ethyl acetate in petroleum ether and then 10% EtOH in ethyl acetate) to give the desired product as a colourless oil (18 mg, 45% yield).

^{1}H NMR (400 MHz, $CDCl_3$) δ 5.98 (s, 1H, H_l), 3.70 – 3.39 (m, 18H, H_e,H_k), 2.00 – 1.93 (m, 3H, H_a), 1.93 (s, 2H, H_d), 1.81 – 1.54 (m, 14H, H_b,H_c,H_f), 1.48 – 1.22 (m, 8H, $H_{g\text{-}j}$).

^{13}C NMR (101 MHz, $CDCl_3$) δ 171.22, 71.41, 70.80, 70.71, 70.68, 70.61, 70.36, 70.27, 70.13, 51.85, 45.17, 42.76, 39.18, 36.94, 32.86, 32.69, 29.56, 28.81, 26.84, 25.55.

MS (ESI+) m/z 488.3121 (expected $[M+H]^+$ $C_{26}H_{47}NO_5Cl^+$ calc. 488.3137).

A stock solution at 200 mM (97.4 mg/mL) was prepared in DMSO for experiments, where appropriate aliquots of this stock was diluted into water.

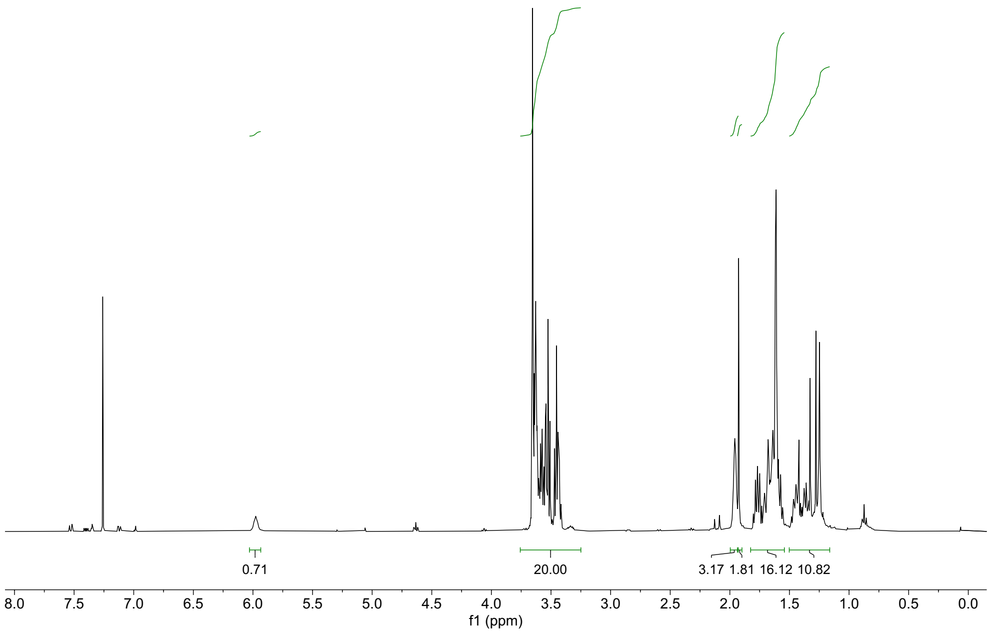
0.71
20.00
3.17 1.81 16.12 10.82
8.0 7.5 7.0 6.5 6.0 5.5 5.0 4.5 4.0 3.5 3.0 2.5 2.0 1.5 1.0 0.5 0.0
f1 (ppm)

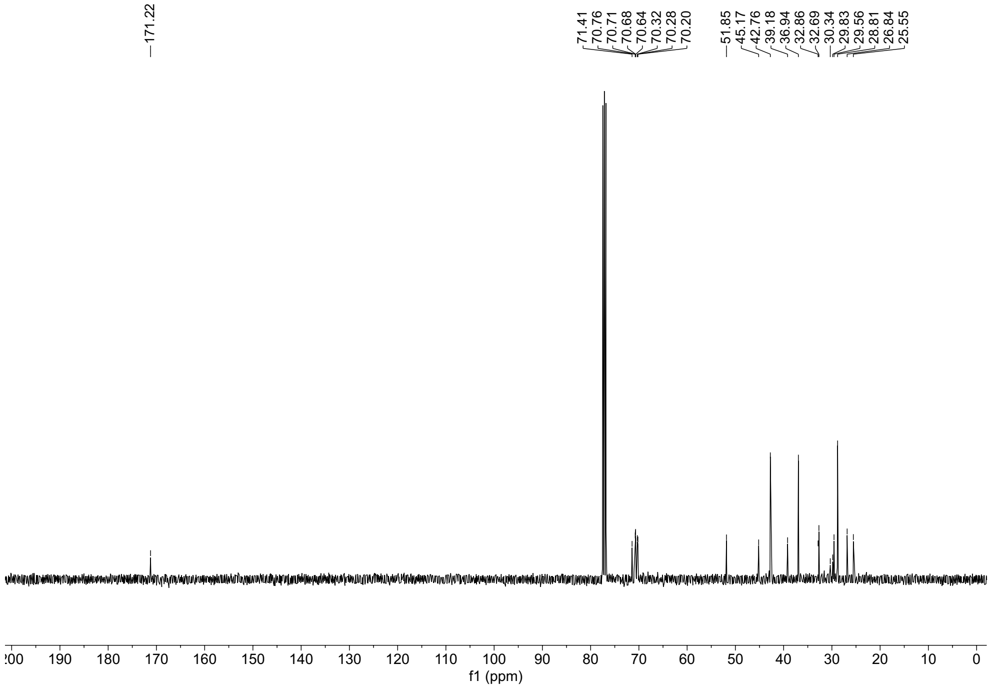
171.22
71.41
70.76
70.71
70.68
70.64
70.32
70.28
70.20
51.85
45.17
42.76
39.18
36.94
32.86
32.69
30.34
29.83
29.56
28.81
26.84
25.55
190 180 170 160 150 140 130 120 110 100 90 80 70 60 50 40 30 20 10 0
f1 (ppm)

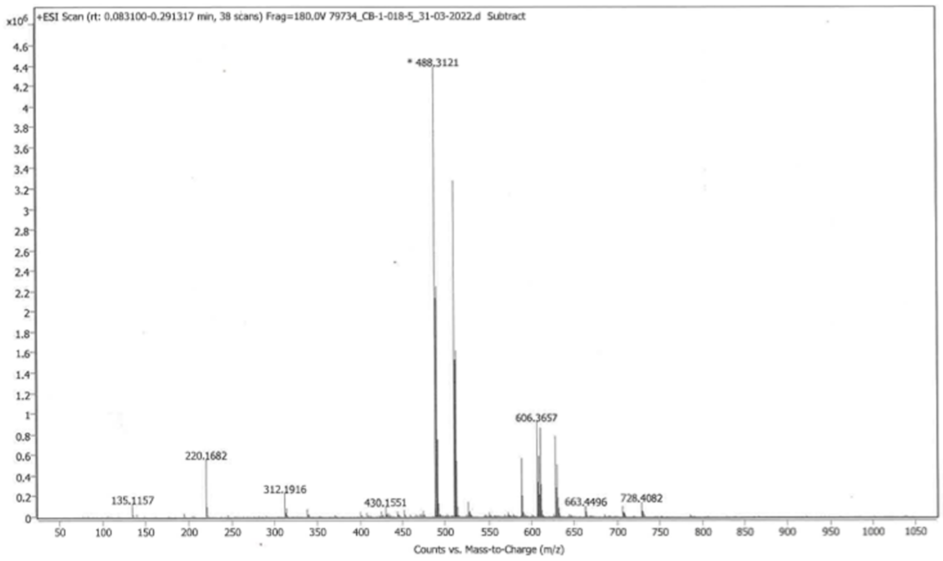

Bromotriethylene glycol monometheyl ether (Br3OMe)

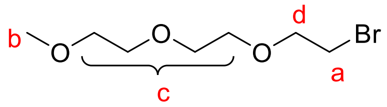

Syntheisised by the method of Karimi *et al.*[23] Triethylene glycol monomethyl ether (4.85 mL, 30.5 mmol) and carbon tetrabromide (12.25 g, 36.5 mmol) were dissolved in 150 mL anhydrous DCM under an inert atmosphere and cooled to 0 °C. Separately, triphenyl phosphine (12.0 g, 45.8 mmol) was dissolved in 50 mL anhydrous DCM under an inert atmosphere. This solution was added dropwise to the solution of triethylene glycol and carbon tertrabromide at 0 °C. The resultant solution was stirred at 0 °C for 3 h and then allowed to warm to RT and stirred for a further 2 h. The solvent was removed *in vacuo*. This yielded the crude product as a yellow oil, which was washed with (3 × 30 mL) diethyl ether. The crude sample was purified by column chromatography (5 % MeOH in chloroform) to give the product as a colourless oil (8.12g, 89.0%). Observed analytical data matched that of the original work.

^{1}H NMR (400 MHz, $CDCl_3$) δ 3.82 (t, J = 6.3 Hz, 2H, Ha), 3.71 – 3.65 (m, 6H, Hc), 3.59 – 3.53 (m, 2H, Hd), 3.48 (t, J = 6.3 Hz, 2H), 3.39 (s, 3H, Hb).

^{13}C NMR (101 MHz, $CDCl_3$) δ 71.95, 71.22, 70.66 – 70.59 (m), 70.55, 59.06, 30.31.

MS (ESI+) *m/z* 249.0104 (expected $[M+Na]^+$ $C_7H_{14}O_3BrNa^+$ calc. 249.0097).

Pyridinium triethylene glycol monomethyl ether (Py3OMe)

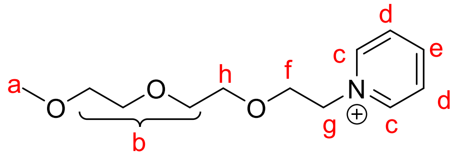

Br3OMe (165 mg, 0.73 mmol) and pyridine (57.7 mg, 0.73 mmol) were dissolved in 1 mL DMF and heated to 100 °C for 20 h. The solvent was removed *in vacuo* and dissolved in 2 mL ethyl acetate and extracted in water (3 × 5 mL), then solvent was removed *in vacuo,* the sample was dissolved in 2 mL DCM and washed with (3 × 5 mL) hexane, yielding the pure product as a yellow oil (41.6 mg, 24.9%). Observed analytical data correlated with previous reports.[24]

^{1}H NMR (400 MHz, $CDCl_3$) δ 9.54 – 9.28 (m, 2H, H_c), 8.41 (tt, J = 7.8, 1.4 Hz, 1H, H_e), 8.00 (t, J = 7.1, 1.4 Hz, 2H, H_d), 5.41 – 4.96 (m, 2H,H_g), 4.12 – 3.95 (m, 2H, H_f), 3.65 – 3.58 (m, 2H, H_h), 3.57 – 3.44 (m, 6H,H_b), 3.31 (s, 3H,H_a).

^{13}C NMR (101 MHz, $CDCl_3$) δ 146.00, 145.03, 127.81, 71.86, 70.41, 70.31, 70.13, 69.40, 61.39, 58.97.

MS (ESI+) *m/z* 226.1438 (expected $[M]^+$ $C_{12}H_{20}NO_3^+$ calc. 226.1438).

1-methyl imidazolium triethylene glycol monomethyl ether (Im3OMe)

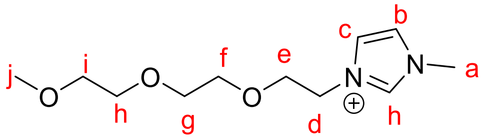

Br3OMe (165 mg, 0.73 mmol) and 1-methyl imidazole (59.9 mg, 0.73 mmol) were dissolved in 1 mL DMF and heated to 100 °C for 20 h. The solvent was removed *in vacuo* and dissolved in 2 mL ethyl acetate before extraction into water (3 × 5 mL), yielding the pure product as a yellow oil (72.2 mg, 43.2%). Observed analytical data correlated with previous reports.[25]

^{1}H NMR (400 MHz, $CDCl_3$) δ 10.14 (t, J = 1.7 Hz, 1H, H_h), 7.73 (t, J = 1.8 Hz, 1H, H_c), 7.33 (t, J = 1.8 Hz, 1H, H_b), 4.66 – 4.59 (m, 2H, H_d), 4.08 (s, 3H, H_a), 3.97 – 3.88 (m, 2H, H_e), 3.72 – 3.64 (m, 2H,H_f), 3.68 – 3.60 (m, 4H, Hg, H_h), 3.60 – 3.53 (m, 2H, H_i), 3.38 (s, 3H, H_j).

^{13}C NMR (101 MHz, $CDCl_3$) δ 167.36,123.83, 122.06, 71.87, 70.31, 70.22, 70.21, 68.98, 58.97, 49.75, 36.49.

MS (ESI+) *m/z* 229.1547 (expected $[M]^+$ $C_{11}H_{21}N_2O_3^+$ calc. 229.1547).

4-dimethylaminopyridinium triethylene glycol monomethyl ether (Ap3OMe)

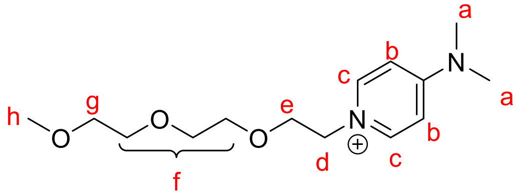

Br3OMe (165 mg, 0.73 mmol) and 4-dimethylaminopyridine (89.2 mg, 0.73 mmol) were dissolved in 1 mL DMF and heated to 100 °C for 20 h. The solvent was removed *in vacuo* and dissolved in 2 mL ethyl acetate and extracted into water (3 × 5 mL), yielding the pure product as a yellow oil (143 mg, 72.9%).

^{1}H NMR (400 MHz, $CDCl_3$) δ 8.66 (d, J = 7.8 Hz, 2H, H_c), 6.93 – 6.86 (d, J = 7.8 Hz, 2H, H_b), 4.69 – 4.63 (m, 2H, H_d), 3.97 – 3.90 (m, 2H, H_e), 3.69 – 3.58 (m, 6H, H_f), 3.57 – 3.53 (m, 2H, H_g), 3.39 (s, 3H, H_h), 3.27 (s, 6H, H_a).

^{13}C NMR (101 MHz, $CDCl_3$) δ 143.45, 107.66, 71.92, 70.39, 70.32, 70.25, 69.82, 59.00, 57.64, 40.32.

MS (ESI+) *m/z* 269.1865 (expected $[M]^+$ C14H25N2O3$^+$ calc. 269.1860).

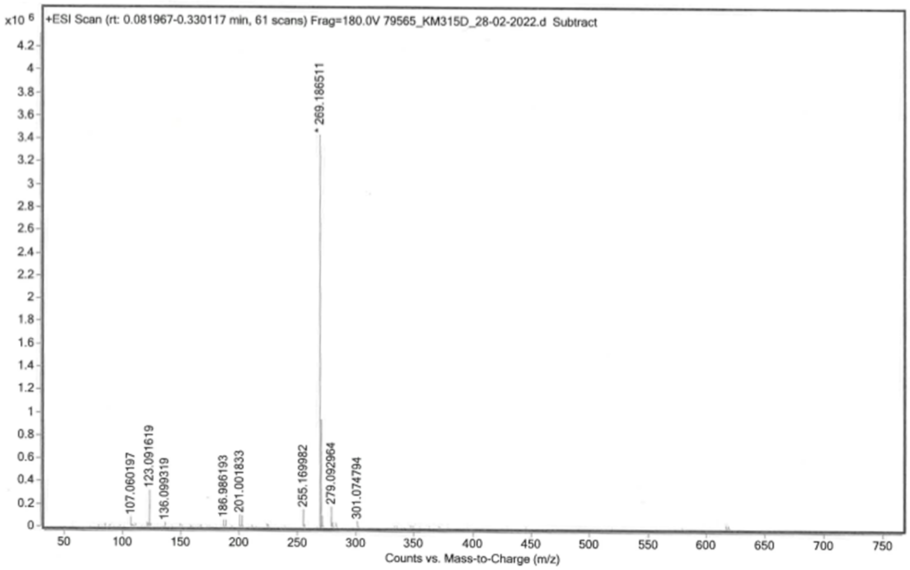

Bromopolyethylene glycol 750 monomethylether (Br16OMe)

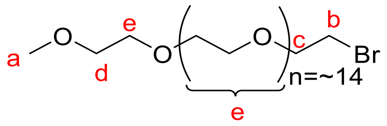

Synthesised adapted from **Br3OMe** above. Poly(ethylene glycol) methyl ether MW750 (c.16 ethylene glycol units, 5 g, 6.66 mmol) and carbon tetrabromide (678 mg, 8 mmol) were dissolved in 50 mL anhydrous DCM. The solution was cooled to 0 °C under an inert atmosphere. Separately, triphenylphosphine (3.3 g, 10 mmol) was dissolved in 5 mL anhydrous DCM under an inert atmosphere and added dropwise to the reaction mixture. The solution was stirred at 0 °C for 3 h and then at RT for 2 h. Solvent was removed *in vacuo* and the sample resuspended in diethyl ether. The resultant solid material was collected by filtration and purified by column chromatography (4 % MeOH in DCM) to yield the product as a white wax (2.02g, 37.3%).

^{1}H NMR (400 MHz, $CDCl_3$) δ 3.78 (t, J = 6.3 Hz, 2H, H_d), 3.62 (d, J = 4.9 Hz, ~50H, H_e), 3.54 – 3.50 (m, 2H, H_c), 3.44 (t, J = 6.3 Hz, 2H, H_b), 3.35 (s, 3H, H_a).

^{13}C NMR (101 MHz, $CDCl_3$) δ 132.13, 132.03, 131.92, 131.89, 128.54, 128.42, 71.92, 71.20, 70.65, 70.58, 70.55, 70.49, 59.01, 30.32.

MS (ESI+) *m/z* 801.3665 (expected [Br16OMe+]$^+$ $C_{33}H_{68}BrO_{16}^+$ calc. 801.3665), 845.3931 (expected [Br17OMe+]$^+$ $C_{35}H_{72}BrO_{17}^+$ calc. 845.3927). Br13OMe-Br23OMe observed in mixture

Pyridinium polyethylene glycol750 monomethyl ether (Py16OMe)

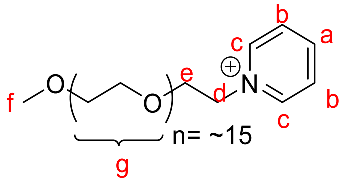

Br16OMe (670 mg, 0.83 mmol) and pyridine (65.5 µL, 0.83 mmol) were dissolved in 1 mL DMF and heated to 80 °C for 3 days. The solution was cooled to RT and the solution was removed *in vacuo.* The residue was suspended in 2 mL water and washed with (3 × 5 mL) chloroform. This gave the product as a white solid (205 mg, 30.1%).

^{1}H NMR (400 MHz, $CDCl_3$) δ 9.52 – 9.45 (m, 2H, H_c), 8.53 (tt, J = 7.8, 1.4 Hz, 1H, H_a), 8.14 – 8.06 (m, 2H, H_b), 5.21 – 5.14 (m, 2H, H_d), 4.09 – 4.02 (m, 2H, H_e), 3.71 – 3.47 (m, ~50H, H_g), 3.35 (s, 3H, H_f).

^{13}C NMR (101 MHz, $CDCl_3$) δ 146.03, 145.36, 127.94, 71.90, 70.49, 70.46, 70.42, 70.21, 69.63, 61.12, 59.04.

MS (ESI+) *m/z* 798.4862 (expected $[M]^+$ $C_{38}H_{72}NO_{16}^+$ calc. 798.4846). Py11OMe-Py23OMe observed

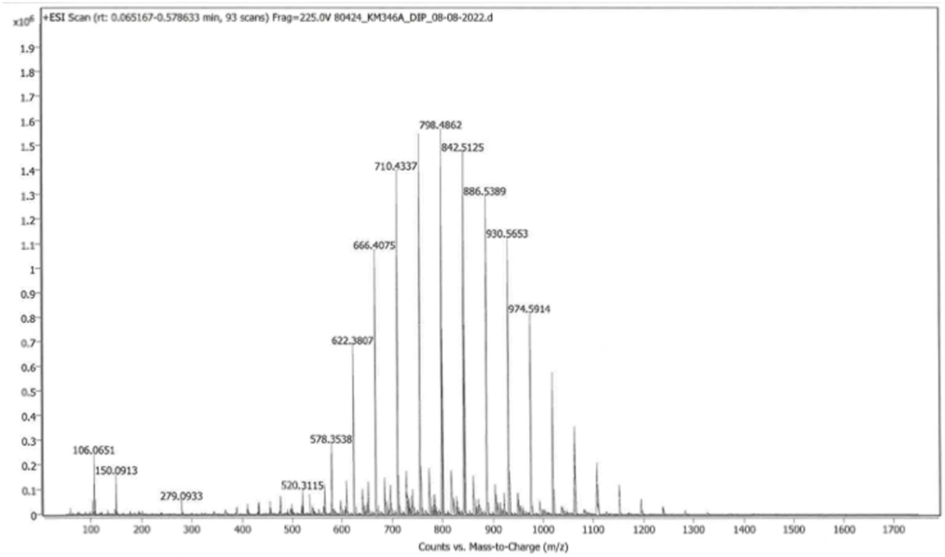

1-methyl imidazolium polyethylene glycol750 monomethyl ether (Im16OMe)

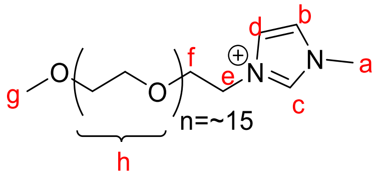

Br16OMe (670 mg, 0.83 mmol) and 1-methyl imidazole (61 µL, 0.83 mmol) were dissolved in 1 mL DMF and heated to 80 °C for 3 days. The solution was cooled to RT and the solution was removed *in vacuo.* The residue was suspended in 2 mL water and washed with (3 × 5 mL) chloroform. This yielded the product as a white solid (370 mg, 54.7%).

^{1}H NMR (400 MHz, $CDCl_3$) δ 10.18 (s, 1H, H_c), 7.70 (d, J = 1.7 Hz, 1H, H_d), 7.31 (d, J = 1.9 Hz, 1H, H_b), 4.59 – 4.53 (m, 2H, H_e), 3.97 (s, 3H, H_a), 3.86 – 3.81 (m, 2H, H_f), 3.58 -3.41 (m, ~50H, H_h), 3.31 (s, 3H, H_g).

^{13}C NMR (125 MHz, $CDCl_3$) δ 137.73, 123.74, 122.84, 71.92, 70.57, 70.52, 70.49, 70.47, 70.45, 70.34, 70.23, 70.20, 69.04, 59.03, 49.69, 36.50.

MS (ESI+) *m/z* 801.4975 (expected $[M]^+$ $C_{37}H_{73}N_2O_{16}^+$ calc. 801.4955).

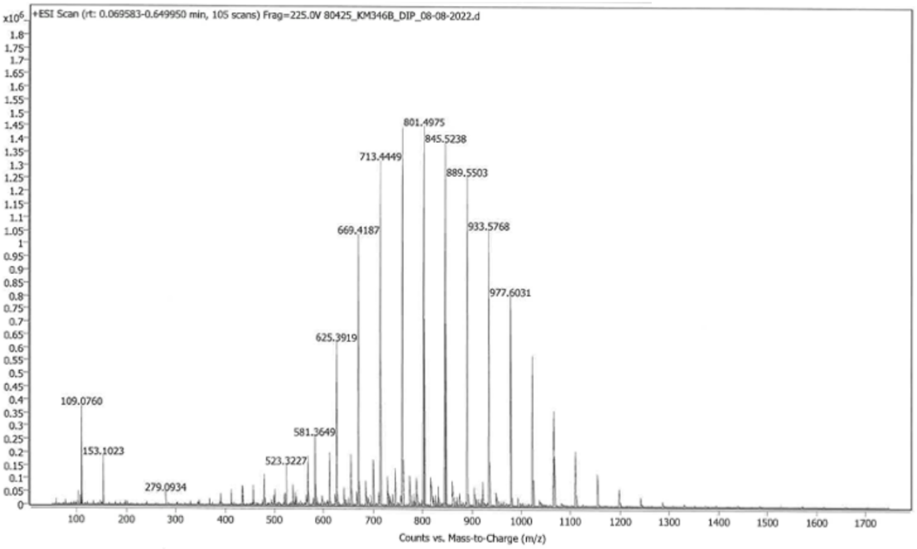

4-dimethylaminopyridinium polyethylene glycol750 monomethyl ether (Ap16OMe)

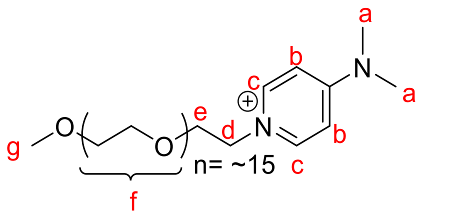

Br16OMe (670 mg, 0.83 mmol) and 4-dimethylaminopyridine (100 mg, 0.83 mmol) were dissolved in 1 mL DMF and heated to 80 °C for 3 days. The solution was cooled to RT and the solution was removed *in vacuo.* The residue was suspended in 2 mL water and washed with (3 × 5 mL) chloroform. This yielded the product as a white solid (470 mg, 66.2%).

^{1}H NMR (400 MHz, $CDCl_3$) δ 8.72 – 8.59 (m, 2H, H_c), 6.91 (d, J = 7.2 Hz, 2H, H_b), 4.71 – 4.59 (t, J = 4.6 Hz, 2H, H_d), 3.93 (t, J = 4.6 Hz, 2H, H_e), 3.70 – 3.53 (m, ~52H, H_f), 3.38 (s, 3H, H_g), 3.27 (s, 6H, H_a).

^{13}C NMR (101 MHz, $CDCl_3$) δ 143.50, 107.64, 71.93, 70.59, 70.54, 70.51, 70.48, 70.42, 70.29, 69.90, 59.04, 57.59, 40.35.

MS (ESI+) *m/z* 841.5287 (expected $[M]^+$ $C_{40}H_{77}N_2O_{16}^+$ calc. 841.5268).

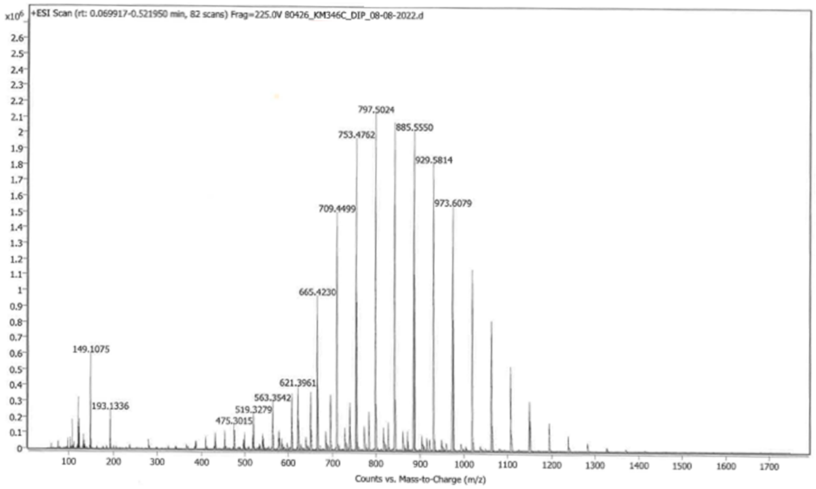

References

(1) Yu, W. W.; Qu, L.; Guo, W.; Peng, X. Experimental Determination of the Extinction Coefficient of CdTe, CdSe, and CdS Nanocrystals. *Chem Mater* **2003**, *15* (14), 2854–2860. https://doi.org/10.1021/cm034081k.

(2) Dong, C.; Ren, J. Measurements for Molar Extinction Coefficients of Aqueous Quantum Dots. *Analyst* **2010**, *135* (6), 1395–1399. https://doi.org/10.1039/c0an00063a.

(3) Gnidovec, A.; Božič, A.; Čopar, S. Dense Packings of Geodesic Hard Ellipses on a Sphere. *Soft Matter* **2022**, *18* (39), 7670–7678. https://doi.org/10.1039/d2sm00624c.

(4) Alnajjar, M. A.; Nau, W. M.; Hennig, A. A Reference Scale of Cucurbit[7]Uril Binding Affinities. *Org Biomol Chem* **2021**, *19* (39), 8521–8529. https://doi.org/10.1039/d1ob01304a.

(5) Tsortos, A.; Papadakis, G.; Mitsakakis, K.; Melzak, K. A.; Gizeli, E. Quantitative Determination of Size and Shape of Surface-Bound DNA Using an Acoustic Wave Sensor. *Biophys J* **2008**, *94* (7), 2706–2715. https://doi.org/10.1529/biophysj.107.119271.

(6) Barrow, S. J.; Kasera, S.; Rowland, M. J.; Barrio, J. del; Scherman, O. A. Cucurbituril-Based Molecular Recognition. *Chem Rev* **2015**, *115* (22), 12320–12406. https://doi.org/10.1021/acs.chemrev.5b00341.

(7) Senler, S.; Cheng, B.; Kaifer, A. E. Rotaxane Formation by Cucurbit[7]Uril in Water and DMSO Solutions. *Org. Lett.* **2014**, *16* (22), 5834–5837. https://doi.org/10.1021/ol502479k.

(8) Lončarić, D.; Movahedifar, F.; Štoček, J. R.; Dračínský, M.; Cvačka, J.; Guan, S.; Bythell, B. J.; Císařová, I.; Masson, E.; Kaleta, J. Solvent-Controlled Formation of Alkali and Alkali-Earth-Secured Cucurbituril/Guest Trimers. *Chem. Sci.* **2023**, *14* (35), 9258–9266. https://doi.org/10.1039/d3sc02032k.

(9) Miskolczy, Z.; Biczók, L.; Megyesi, M.; Jablonkai, I. Inclusion Complex Formation of Ionic Liquids and Other Cationic Organic Compounds with Cucurbit[7]Uril Studied by 4′,6-Diamidino-2-Phenylindole Fluorescent Probe. *J. Phys. Chem. B* **2009**, *113* (6), 1645–1651. https://doi.org/10.1021/jp8098329.

(10) Wyman, I. W.; Macartney, D. H. Cucurbit[7]Uril Host-Guest and Pseudorotaxane Complexes with α,ω-Bis(Pyridinium)Alkane Dications. *Org. Biomol. Chem.* **2009**, *7* (19), 4045–4051. https://doi.org/10.1039/b910322h.

(11) Ninomiya, S.; Adachi, S. Optical Properties of Cubic and Hexagonal CdSe. *J Appl Phys* **1995**, *78* (7), 4681–4689. https://doi.org/10.1063/1.359815.

(12) Tran, D. P.; Macdonald, T. J.; Wolfrum, B.; Stockmann, R.; Nann, T.; Offenhäusser, A.; Thierry, B. Photoresponsive Properties of Ultrathin Silicon Nanowires. *Applied Physics Letters* **2014**, *105* (23), 231116. https://doi.org/10.1063/1.4904089.

(13) Shen, H.; Wang, H.; Tang, Z.; Niu, J. Z.; Lou, S.; Du, Z.; Li, L. S. High Quality Synthesis of Monodisperse Zinc-Blende CdSe and CdSe/ZnS Nanocrystals with a Phosphine -Free Method. *CrystEngComm* **2009**, *11* (8), 1733–1738. https://doi.org/10.1039/b909063k.

(14) Peveler, W. J.; Roldan, A.; Hollingsworth, N.; Porter, M. J.; Parkin, I. P. Multichannel Detection and Differentiation of Explosives with a Quantum Dot Array. *ACS Nano* **2016**, *10* (1), 1139–1146. https://doi.org/10.1021/acsnano.5b06433.

(15) Peveler, W. J.; Jia, H.; Jeen, T.; Rees, K.; Macdonald, T. J.; Xia, Z.; Chio, W.-I. K.; Moorthy, S.; Parkin, I. P.; Carmalt, C. J.; Algar, W. R.; Lee, T.-C. Cucurbituril-Mediated Quantum Dot Aggregates Formed by Aqueous Self-Assembly for Sensing Applications. *Chem Commun* **2019**, *55* (38), 5495–5498. https://doi.org/10.1039/c9cc00410f.

(16) Jeen, T.; Algar, W. R. Mimicking Cell Surface Enhancement of Protease Activity on the Surface of a Quantum Dot Nanoparticle. *Bioconjug Chem* **2018**, *29* (11), 3783–3792. https://doi.org/10.1021/acs.bioconjchem.8b00647.

(17) Day, A.; Arnold, A. P.; Blanch, R. J.; Snushall, B. Controlling Factors in the Synthesis of Cucurbituril and Its Homologues. *J Org Chem* **2001**, *66* (24), 8094–8100. https://doi.org/10.1021/jo015897c.

(18) Kim, J.; Jung, I.-S.; Kim, S.-Y.; Lee, E.; Kang, J.-K.; Sakamoto, S.; Yamaguchi, K.; Kim, K. New Cucurbituril Homologues: Syntheses, Isolation, Characterization, and X-Ray Crystal Structures of Cucurbit[n]uril (N= 5, 7, and 8). *J Am Chem Soc* **2000**, *122* (3), 540–541. https://doi.org/10.1021/ja993376p.

(19) Gustafson, J. L.; Neklesa, T. K.; Cox, C. S.; Roth, A. G.; Buckley, D. L.; Tae, H. S.; Sundberg, T. B.; Stagg, D. B.; Hines, J.; McDonnell, D. P.; Norris, J. D.; Crews, C. M. Small-Molecule-Mediated Degradation of the Androgen Receptor through Hydrophobic Tagging. *Angewandte Chemie Int Ed* **2015**, *54* (33), 9659–9662. https://doi.org/10.1002/anie.201503720.

(20) Li, X.; Lü, Z.; Wang, C.; Li, K.; Xu, F.; Xu, P.; Niu, Y. Induction of Apoptosis in Cancer Cells by Glutathione Transferase Inhibitor Mediated Hydrophobic Tagging Molecules. *ACS Med Chem Lett* **2021**, *12* (5), 720–725. https://doi.org/10.1021/acsmedchemlett.0c00627.

(21) Zhang, X.; Wu, X.; Jiang, S.; Gao, J.; Yao, Z.; Deng, J.; Zhang, L.; Yu, Z. Photo-Accelerated “Click” Reaction between Diarylsydnones and Ring-Strained Alkynes for Bioorthogonal Ligation. *Chem Commun* **2019**, *55* (50), 7187–7190. https://doi.org/10.1039/c9cc02882j.

(22) Steinebach, C.; Sosič, I.; Lindner, S.; Bricelj, A.; Kohl, F.; Ng, Y. L. D.; Monschke, M.; Wagner, K. G.; Krönke, J.; Gütschow, M. A MedChem Toolbox for Cereblon-Directed PROTACs. *Medchemcomm* **2019**, *10* (6), 1037–1041. https://doi.org/10.1039/c9md00185a.

(23) Karimi, B.; Mansouri, F.; Vali, H. A Highly Water-Dispersible/Magnetically Separable Palladium Catalyst Based on a Fe 3 O 4 @SiO 2 Anchored TEG-Imidazolium Ionic Liquid for the Suzuki–Miyaura Coupling Reaction in Water. *Green Chem* **2014**, *16* (5), 2587–2596. https://doi.org/10.1039/c3gc42311e.

(24) Wang, J.; Zeng, S.; Bai, L.; Gao, H.; Zhang, X.; Zhang, S. Novel Ether-Functionalized Pyridinium Chloride Ionic Liquids for Efficient SO2 Capture. *Ind Eng Chem Res* **2014**, *53* (43), 16832–16839. https://doi.org/10.1021/ie5027265.

(25) Fu, S.; Liu, S. Preparation of Functionalized Imidazolium Salts under Microwave Irradiation. *Synthetic Commun* **2006**, *36* (14), 2059–2067. https://doi.org/10.1080/00397910600634464.
